# A Series of Metal–Organic Frameworks with 2,2′-Bipyridyl Derivatives: Synthesis vs. Structure Relationships, Adsorption, and Magnetic Studies

**DOI:** 10.3390/molecules28052139

**Published:** 2023-02-24

**Authors:** Vadim A. Dubskikh, Aleksei A. Kolosov, Anna A. Lysova, Denis G. Samsonenko, Alexander N. Lavrov, Konstantin A. Kovalenko, Danil N. Dybtsev, Vladimir P. Fedin

**Affiliations:** 1 Nikolaev Institute of Inorganic Chemistry, Siberian Branch of the Russian Academy of Sciences, Novosibirsk 630090, Russia; 2 Department of Natural Sciences, Novosibirsk State University, Novosibirsk 630090, Russia

**Keywords:** manganese(II), metal-organic framework, 2,2′-bithiophen-5,5′-dicarboxylic acid, gas adsorption, adsorption selectivity, magnetic properties

## Abstract

Five new metal–organic frameworks based on Mn(II) and 2,2′-bithiophen-5,5′-dicarboxylate (btdc^2–^) with various chelating N-donor ligands (2,2′-bipyridyl = bpy; 5,5′-dimethyl-2,2′-bipyridyl = 5,5′-dmbpy; 4,4′-dimethyl-2,2′-bipyridyl = 4,4′-dmbpy) [Mn_3_(btdc)_3_(bpy)_2_]·4DMF, **1**; [Mn_3_(btdc)_3_(5,5′-dmbpy)_2_]·5DMF, **2**; [Mn(btdc)(4,4;-dmbpy)], **3**; [Mn_2_(btdc)_2_(bpy)(dmf)]·0.5DMF, **4**; [Mn_2_(btdc)_2_(5,5′-dmbpy)(dmf)]·DMF, **5** (dmf, DMF = N,N-dimethylformamide) have been synthesized, and their crystal structure has been established using single-crystal X-ray diffraction analysis (XRD). The chemical and phase purities of Compounds **1**–**3** have been confirmed via powder X-ray diffraction, thermogravimetric, and chemical analyses as well as IR spectroscopy. The influence of the bulkiness of the chelating N-donor ligand on the dimensionality and structure of the coordination polymer has been analyzed, and the decrease in the framework dimensionality, as well as the secondary building unit’s nuclearity and connectivity, has been observed for bulkier ligands. For three-dimensional (3D) coordination polymer **1**, the textural and gas adsorption properties have been studied, revealing noticeable ideal adsorbed solution theory (IAST) CO_2_/N_2_ and CO_2_/CO selectivity factors (31.0 at 273 K and 19.1 at 298 K and 25.7 at 273 K and 17.0 at 298 K, respectively, for the equimolar composition and the total pressure of 1 bar). Moreover, significant adsorption selectivity for binary C_2_–C_1_ hydrocarbons mixtures (33.4 and 24.9 for C_2_H_6_/CH_4_, 24.8 and 17.7 for C_2_H_4_/CH_4_, 29.3 and 19.1 for C_2_H_2_/CH_4_ at 273 K and 298 K, respectively, for the equimolar composition and the total pressure of 1 bar) has been observed, making it possible to separate on **1** natural, shale, and associated petroleum gas into valuable individual components. The ability of Compound **1** to separate benzene and cyclohexane in a vapor phase has also been analyzed based on the adsorption isotherms of individual components measured at 298 K. The preferable adsorption of C_6_H_6_ over C_6_H_12_ by **1** at high vapor pressures (*V*_B_/*V*_CH_ = 1.36) can be explained by the existence of multiple van der Waals interactions between guest benzene molecules and the metal–organic host revealed by the XRD analysis of **1** immersed in pure benzene for several days (**1≅*2*C_6_H_6_**). Interestingly, at low vapor pressures, an inversed behavior of **1** with preferable adsorption of C_6_H_12_ over C_6_H_6_ (*K*_CH_/*K*_B_ = 6.33) was observed; this is a very rare phenomenon. Moreover, magnetic properties (the temperature-dependent molar magnetic susceptibility, χ_p_(*T*) and effective magnetic moments, μ_eff_(*T*), as well as the field-dependent magnetization, *M*(*H*)) have been studied for Compounds **1**–**3**, revealing paramagnetic behavior consistent with their crystal structure.

## 1. Introduction

Metal–organic frameworks (MOFs) are a class of inorganic materials consisting of metal ions or clusters connected by organic ligands into one- (1D), two- (2D), or three- (3D) dimensional structures with pores whose size is determined by the length and geometry of the organic ligand [1,2,3,4,5,6]. Currently, various ligands with two or more carboxylate functions are the most extensively used types of linkers for the MOF design. Polydentate carboxylate groups are known to assist an assemblage of metal cations into polynuclear clusters or complexes known as secondary building units (SBUs) to be connected into regular periodic networks. Auxiliary monodentate linkers, e.g., with pyridine functions, such as 4,4′-bipyridyl, further enrich the connectivity of SBUs and the complexity of the topology of MOFs, which, however, may have a negative impact on the porosity of the material [7,8,9,10]. In this regard, a reduction in the structural complexity might often be a promising strategy in the design of sufficiently porous structures. Additionally, a suppression of the connectivity of the SBUs is likely the only rational way to obtain low-dimensional coordination networks (1D or 2D), which are valuable targets for magnetic or electronic materials with highly anisotropic properties [11,12,13,14,15]. Chelate organic ligands are known to form stronger coordination complexes with metal cations ensuring higher competitively over the other type of ligands. Partial substitution of carboxylate linkers to a chelate pendant in the inner coordination sphere of metal cations inevitably reduces the connectivity of the SBUs. Additionally, a substitution of coordinated solvent molecules by a chelate ligand improves the general stability of the porous architecture of the MOF since the expected detachment of the poorly bind moieties may destabilize the coordination environment of the metal nodes. Moreover, electron-rich aromatic chelate molecules, such as 1,10-phenantroline or 2,2′-bipyridyl, feature excellent UV light absorbance, resulting in a remarkable luminescence or photocatalytic properties of the corresponding coordination compounds [16,17,18,19]. In short, the utilization of auxiliary chelate ligands is a powerful tool for a deliberate modulation of a MOF topology, an enhancement of the stability and porosity of the coordination framework, as well as a versatile functionalization of the products.

Despite obvious advantages, the systematic study of the effects of the chelating ligands on the structure and functional properties of the MOFs is somewhat underdeveloped [20,21,22,23,24,25,26]. In this account we investigate a synthesis of a series of five new MOFs prepared from Mn(II) cations; 2,2′-bithiophen-5,5′-dicarboxylate anions as ditopic linkers; and 2,2′-bipyridyl derivatives (2,2′-bipyridyl = bpy; 5,5′-dimethyl-2,2′-bipyridyl = 5,5′-dmbpy; 4,4′-dimethyl-2,2′-bipyridyl = 4,4′-dmbpy) as chelate ligands. An influence of the bipyridyl derivative on the structure of SBUs and topology of coordination networks was revealed. The adsorption and potential selective separation properties of one of the MOFs featuring 3D porous structure towards some industrially important gases or vapors have been systematically investigated. Moreover, various magnetic properties of the MOFs have been characterized and rationalized according to their crystal structures.

## 2. Results and Discussion

### 2.1. Synthesis and Structural Characterization

**Scheme 1:**
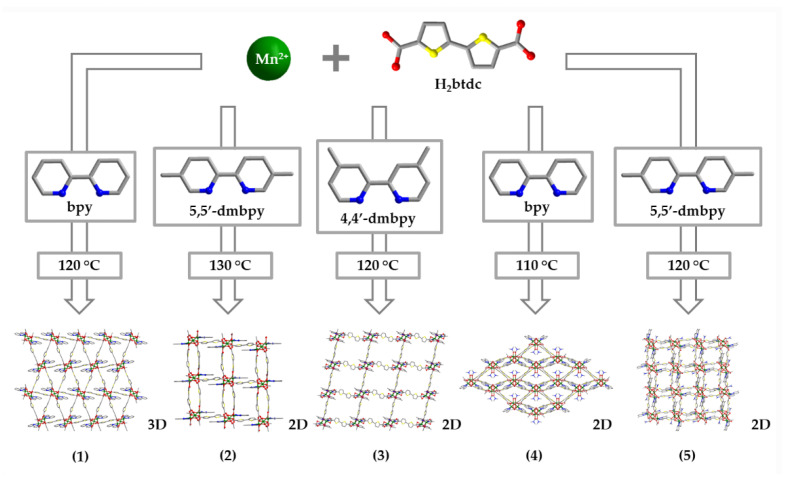
Schematic description of the synthesis conditions for **1**–**5**.

All compounds are formed under similar reaction conditions: manganese(II) perchlorate hexahydrate as a source of the metal ions, the equimolar ratio of the metal ions and H_2_btdc, DMF as a solvent, and high synthesis temperature (110–130 °C) (Scheme 1). The syntheses of Compounds **1**–**3**, which have been obtained as pure phases, are reproducible with comparable yields (46–61%). Compounds **4** and **5** were obtained as impurities to Compounds **1** and **2**, respectively, when trying to synthesize them under other conditions.

**Figure 1:**
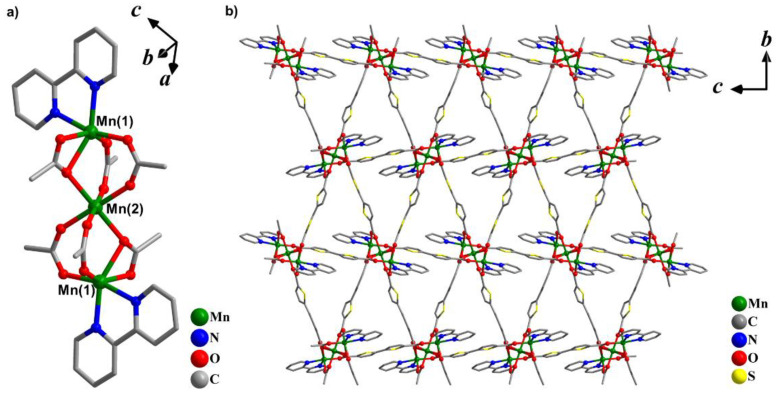
(**a**) Structure of {Mn_3_(μ-RCOO-κ^1^,κ^1^)_4_(μ-RCOO-κ^1^,κ^2^)_2_(bpy)_2_} building unit in **1**; (**b**) fragment of the crystal structure of **1** (projection on the *bc* plane).

The colorless plate crystals of [Mn_3_(btdc)_3_(bpy)_2_]·4DMF (**1**) are formed by heating the Mn(ClO_4_)_2_·6H_2_O, H_2_btdc and bpy mixture in DMF at 120 °C for two days. According to the X-ray crystallography data, Compound **1** has a 3D framework structure. An asymmetric unit contains two Mn(II) cations. Mn(1) cation is in a distorted octahedral coordination environment consisted from two oxygen atoms of two monodentate carboxylate groups, two oxygen atoms of a chelating bidentate carboxylate group, and two nitrogen atoms from a chelating bpy molecule. One of the rings of the bpy molecule is disordered over two half-occupied positions. Mn(2) adopts an octahedral geometry with six oxygen atoms of six different carboxylate groups. Mn(2) and two Mn(1) cations are interconnected via bridging COO groups to form a trinuclear secondary building unit (SBU) {Mn_3_(μ-RCOO-κ^1^,κ^1^)_4_(μ-RCOO-κ^1^,κ^2^)_2_(bpy)_2_} (Figure 1a). The Mn(1)–O bond lengths are in the range 2.065(2)–2.272(2) Å, the Mn(1)–N distances are in the range 2.201(2)–2.294(5) Å, and the Mn(2)–O distances lay in the range 2.1313(17)–2.1969(16) Å. Each trinuclear unit in **1** is linked to six others via six bridging btdc^2–^ anions, which results in a 3D framework (Figure 1b). The thiophene rings of all btdc^2–^ anions are in trans positions, and the angles between them are ~157° and ~180°. Compound **1** possesses 1D quadrilateral channels of the size of 5×4 Å running along the *c* axis. The channels are occupied by disordered guest DMF molecules. The guest composition is defined from the single crystal X-ray diffraction (XRD). The guest accessible void volume of **1** estimated by a PLATON software [27] is 34%.

**Figure 2:**
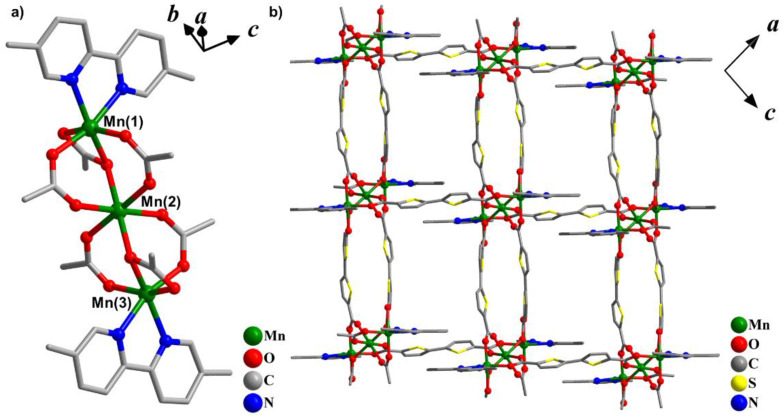
(**a**) Structure of {Mn_3_(μ-RCOO-κ^1^,κ^1^)_4_(μ-RCOO-κ^1^,κ^2^)_2_(5,5′-dmbpy)_2_} building unit in **2**; (**b**) fragment of the layer in Structure **2** (projection on the *ac* plane).

The colorless plate crystal of [Mn_3_(btdc)_3_(5,5′-dmbpy)_2_]·5DMF (**2**) is formed by heating the Mn(ClO_4_)_2_·6H_2_O, H_2_btdc and 5,5′-dmbpy mixture in DMF at 130 °C for two days. According to the X-ray crystallography data, Compound **2** contains similar trinuclear SBU (Figure 2a). However, the coordination environments of two terminal Mn(II) ions slightly differ in dihedral angles and bond lengths. An asymmetric unit of **2** contains three nonequivalent manganese ions. The Mn(1)–O bond lengths are in the range 2.1025(12)–2.3296(11) Å, the Mn(1)–N distances are 2.2233(14) Å and 2.2595(14) Å, the Mn(2)–O distances lay in the range 2.1423(11)–2.2057(11) Å, the Mn(3)–O bond lengths are in the range 2.0990(11)–2.3372(11) Å, and the Mn(3)–N distances are 2.2120(14) Å and 2.2635(14) Å. In contrast to Compound **1**, the thiophene rings of the btdc^2−^ anion are in the cis positions. At the moment, only one coordination polymer has been published where the btdc^2−^ ligand adopts the cis position [28]. Each SBU in **2** is linked to four others via two double and two single btdc^2−^ bridges, which results in a two-dimensional (2D) network with rectangular windows of 4×6 Å (Figure 2b). The layers are parallel to the *ac* plane and alternate along the *b* axis to form two-layered crystal packing. Partially disordered guest DMF molecules are located in the interlayer space and in the windows of the layers. The guest-accessible void volume of **2** estimated by a PLATON software [27] is 41%.

The trinuclear linear SBU are very common in the chemistry of MOFs resulting in 2D or 3D structures [29,30,31,32,33]. The terminal positions in the coordination environment of the terminal metal ions are commonly occupied by coordinated solvent molecules. In our case, we intentionally used an additional chelating N-donor ligand in order to move away from the [Mn_3_(btdc)_3_(dmf)_4_] compound, which is based on the trinuclear SBU with coordinated DMF molecules on both terminal manganese(II) ions formed typically in the system with Mn(II) and H_2_btdc in the DMF solvent [34].

**Figure 3:**
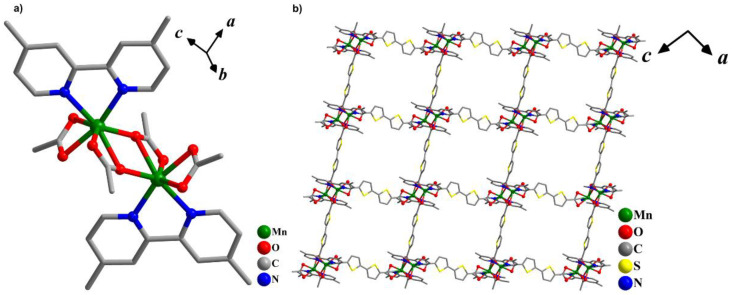
(**a**) Structure of {Mn_2_(μ-RCOO-κ^1^,κ^2^)_2_(4,4′-dmbpy)_2_(RCOO-κ^2^)_2_} building unit in **3**; (**b**) fragment of the layer in Structure **3** (projection on the *ac* plane).

The colorless plate crystal of [Mn(btdc)(4,4′-dmbpy)] (**3**) is obtained by heating the Mn(ClO_4_)_2_·6H_2_O, H_2_btdc and 4,4′-dmbpy mixture in DMF at 120 °C for two days. The much bulkier N-donor ligand results in the decrease of the nuclearity of SBU; Compound **3** is based on a binuclear {Mn_2_(RCOO)_4_} building block consisting of two equivalent manganese(II) ions. According to X-ray crystallography data, Mn(II) has distorted pentagonal bipyramidal coordination environment, which contains two oxygen atoms of one chelating carboxylate group, three oxygen atoms of two bridging carboxylate groups, and two nitrogen atoms of a chelating 4,4′-dmbpy molecule. Two Mn(II) cations are interconnected via two bridging COO groups to form binuclear unit {Mn_2_(μ-RCOO-κ^1^,κ^2^)_2_(4,4′-dmbpy)_2_(RCOO-κ^2^)_2_} (Figure 3a). The Mn–O bond lengths are in the range 2.1980(16)–2.3390(16) Å, and the Mn–N distances are 2.2331(19) Å and 2.2881(18) Å. The thiophene rings of all btdc^2–^ anions are in trans positions, with the dihedral angle of 180° between their planes. Each binuclear building unit in **3** is connected by four single btdc^2−^ bridges to four others acting as a four-connecting node. This leads to the formation of layers with rectangular windows of 5 × 11 Å. The layers are situated on (−2 4 −2) and (2 −4 2) crystallographic planes to form two-layered crystal packing. The layers are tightly packed without the free volume available for guest solvent molecules.

**Figure 4:**
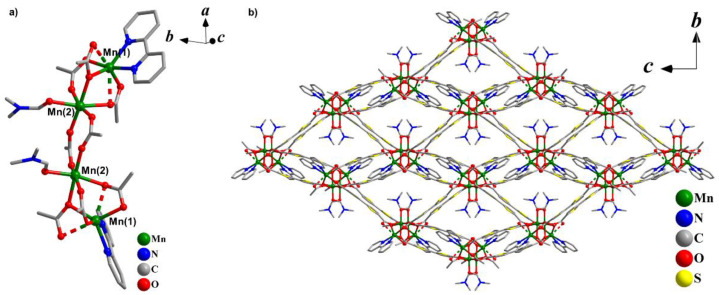
(**a**) Structure of {Mn_4_(μ-RCOO-κ^1^,κ^1^)_4_(μ-RCOO-κ^1^,κ^2^)_4_(bpy)_2_(dmf)_2_} building unit in **4**; (**b**) fragment of the layer in Structure **4** (projection on the *bc* plane). Only one of four positions of coordinated DMF molecules is shown.

At a lower temperature (110 °C), the colorless block crystals of [Mn_2_(btdc)_2_(bpy)(dmf)]·0.5DMF (**4**) are isolated as a byproduct during the synthesis of Compound **1** with the reagent ratio [Mn^2+^]:[btdc^2-^]:[bpy] = 2:2:1. According to X-ray crystallography data, an asymmetric unit of **4** contains two Mn(II) cations. The coordination environment of Mn(1) ion contains 5 oxygen atoms of 3 carboxylic groups and 2 nitrogen atoms of a bpy molecule. Two of three COO groups are coordinated in a bidentate manner with a short Mn–O bond and a longer Mn···O contact. Coordination number of Mn(1) could be described as 5 + 2. The Mn(1)–O bond lengths are in the range 2.113(2)–2.239(3) Å, Mn(1)···O contacts are 2.506(3) and 2.663(3) Å, and Mn(1)–N bond lengths are 2.228(3) Å and 2.234(3) Å. Mn(2) are in octahedral coordination environment, which consists of 5 oxygen atoms of 5 carboxylate groups, and one oxygen atom of a coordinated DMF molecule disordered over four positions. Mn(2)–O distances are in the range 2.086(3)–2.227(3) Å. Mn(1) and Mn(2) cations are interconnected via 3 bridging COO-groups to form a binuclear unit. Two binuclear units are interconnected via two bridging carboxylic groups to form a tetranuclear building unit {Mn_4_(μ-RCOO-κ^1^,κ^1^)_4_(μ-RCOO-κ^1^,κ^2^)_4_(bpy)_2_(dmf)_2_} (Figure 4a). The thiophene rings of a half btdc^2−^ anions are in the cis positions, whereas those of another half btdc^2−^ anions are in the trans positions. Each tetranuclear SBU is connected by four double btdc^2−^ bridges to four neighboring ones, which leads to the formation of wide layers (Figure 4b) parallel to the *bc* plane. The layers alternate along the *a* axis to form the one-layered crystal packing. Compound **4** has triangular channels of the size of 5 × 8 Å running along the *a* axis. The channels are occupied by disordered coordinated and guest DMF molecules. The guest-accessible void volume of **4** estimated using PLATON software [27] is only 7%.

**Figure 5:**
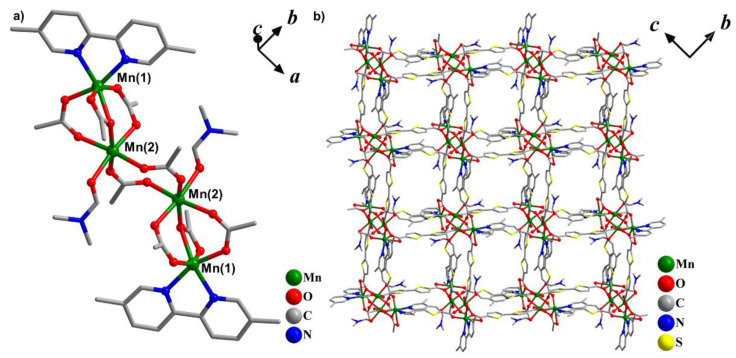
(**a**) Structure of {Mn_4_(μ-RCOO-κ^1^,κ^1^)_6_(μ-RCOO-κ^1^,κ^2^)_2_(4,4′-dmbpy)_2_(dmf)_2_} building unit in **5**; (**b**) fragment of the layer in Structure **5** (projection on the *bc* plane).

At a slightly lower temperature than for the synthesis of pure phase **2** (120 °C) the colorless block crystals of [Mn_2_(btdc)_2_(5,5′-dmbpy)(dmf)]·DMF (**5**) are isolated as a byproduct in the course of the synthesis of Compound **2** with the reagent ratio [Mn^2+^]:[btdc^2−^]:[5,5′-dmbpy] = 2:2:1. According to the X-ray crystallography data, Compound **5** is also based on a tetranuclear SBU, but its structure is different from that in Compound **4**. The asymmetric unit of **5** contains two Mn(II) cations. The Mn(1) cations are in a distorted octahedral coordination environment composed of four oxygen atoms of three carboxylic groups and two nitrogen atoms of a chelating 4,4′-dmbpy molecule. The octahedral coordination environment of Mn(2) consists of five oxygen atoms of five carboxylic groups and an oxygen atom of a coordinated DMF molecule. The Mn(1)–O bond lengths are in the range 2.0861(17)–2.3848(18) Å, the Mn(1)–N distances are 2.221(2) Å and 2.246(2) Å, and the Mn(2)–O distances lay in the range 2.1242(16)–2.2310(17) Å. By the use of the bridging carboxylate groups, the manganese atoms are interconnected in sequence Mn(1)–Mn(2)–Mn(2)–Mn(1) in the form of a linear tetranuclear building unit {Mn_4_(μ-RCOO-κ^1^,κ^1^)_6_(μ-RCOO-κ^1^,κ^2^)_2_(4,4′-dmbpy)_2_(dmf)_2_} (Figure 5a). The center of the tetranuclear unit is situated in the inversion center. The thiophene rings of all btdc^2−^ anions are in the *trans*-positions. Each tetranuclear SBU is connected by four double btdc^2−^ bridges to four neighboring ones resulting in the formation of wide layers with quadrilateral windows of 5 × 3 Å (Figure 5b). The layers alternate along [1 0 −1] direction to form two-layered crystal packing. There are channels running along the a axis, which are filled by the coordinated and guest DMF molecules. The guest-accessible void volume of **5** estimated using PLATON software [27] is 25%.

The majority of the synthetic conditions ([Mn^2+^]/[btdc^2-^] ratio, concentrations, temperature range, solvent, reaction time) for **1**–**5** are either identical or very similar, while [Mn^2+^]/[bpy] ratio as well as positions of the methyl substituents in the bpy molecule are the only variable parameters. Such experimental design reveals a number of interesting relationships between the reaction conditions and the structure of SBUs as well as the topology of the MOF products. Decreasing the [Mn^2+^]/[bpy] molar ratio in the reaction mixture from 2:1 in the synthesis of **4** and **5** to 1:2 in the synthesis of **1** and **2** consequently lowers the [Mn^2+^]/[bpy] composition in the chemical formulae of the products since [Mn^2+^]/[bpy] = 4:2 in **4** and **5** and 3:2 in **1** and **2**. Additionally, the structure of the carboxylate SBUs in those compounds is changed accordingly. The effect of the chemical functionalization of the bpy core could be seen via comparison of **1** vs. **2** and **4** vs. **5**. In those cases, the methyl substituents in bpy ligands do not seem to influence the chemical composition or local features of the crystal structure (i.e., building blocks, SBU). To a certain extent, such a result could be expected since the structure of the SBU is mainly determined by a coordination arrangement of the ligands around the metal cations; however, the methyl groups in 5,5′-dmbpy neither have an impact on the geometry of the bpy molecule nor impose a steric hindrance for the bpy coordination. On the other hand, the methyl pendants attached to the bpy ligands decorate a periphery of the carboxylate SBUs and may develop some limitations to the packing of those large units as well as the topology of the coordination network. As a result, the same trinuclear SBU {Mn_3_(RCOO)_6_(bpy)_2_} in Compound **1** acts as a six-connecting node to form a 3D framework, while in **2**, its connectivity is reduced to 4, forming a 2D layered net. Additionally, the relative porosity of **1** (34%) and **4** (7%) containing bare bpy ligands turns out to be lower than that of **2** (41%) and **5** (25%) containing 5,5′-dmbpy, respectively. We can speculate that the pendant methyl groups on the surface of the SBUs create certain sterical constraints, which hinder a denser packing of these units and eventually lead to an increase in pore volumes of the final MOF products. Quite strikingly, Compound **3**, containing 4,4′-dmbpy ligand, features completely different chemical formula and structure of the SBU. The electron-donor group, such as methyl, in *para*-position is known to increase the nucleophility of the N donor atom of the pyridine ring and, eventually, the strength of the coordination interactions. This seems to be a deciding factor as the ratio [bpy]/[Mn^2+^] = 1:1 in **3** is the highest among the title series **1–5**, which clearly suggests stronger coordination ability of the 4,4′-dmbpy ligand compared to that of 5,5′-dmbpy or nonsubstituted bpy molecules.

### 2.2. IR spectroscopy, Thermal and Textural Properties

Compounds **1**–**3** were obtained as pure phases, as confirmed using the powder X-ray diffraction method (Figures S1–S3), chemical analyses, and optical images (Figure S4); hence, their functional properties were further characterized.

The IR spectra of **1**–**3** contain bands at 770 cm^−1^ (**1**), 764 and 805 cm^−1^ (**2**), and 773 cm^−1^ (**3**), which can be related to the nonplanar deformation vibrations of C–H bonds in the thiophene fragment (Figures S5–S7). It is surprising that the spectrum of Compound **2** contains two bands corresponding to this vibration. This may be due to the cis position of the thiophene rings of the btdc^2–^ anion in this compound. The characteristic bands of the carboxylate groups at 1377 and 1436 cm^−1^ (**1**), at 1389 and 1439 cm^−1^ (**2**), and at 1378 and 1426 cm^−1^ (**3**) are related to the symmetric stretching vibrations. The strong bands of the stretching asymmetric vibrations of the C=O bond in the carboxylate groups are observed at 1515 and 1594 cm^–1^ (**1**), at 1519 and 1564 cm^–1^ (**2**), and at 1522 and 1595 cm^–1^ (**3**). The bands at 1665 cm^−1^ (**1**) and 1612 cm^−1^ (**2**) can be referred to C=O bonds in DMF molecules, whereas the low-intensity bands in the range from 2850 cm^−1^ to 3091 cm^−1^ correspond to the valence vibrations of the C–H bonds in the CH_3_ fragment of the DMF molecule (**1**), the DMF or 5,5′-dmbpy molecules (**2**), and the 4,4′-dmbpy molecule (**3**). The broad bands at 3352 cm^−1^ (**1**), 3415 cm^−1^ (**2**), 3432 cm^−1^ (**3**) correspond to the airborne water on the surface of the crystals.

The thermogravimetric (TG) analysis of Compound **1** shows a continuous loss of mass in a wide temperature range (up to ~220 °C) of ca. 19%, which is associated with removal of the guest solvent molecules (calculated: 19% for 4 DMF) followed by a broad flat region up to ca. 310 °C, where the MOF starts to degrade quickly (Figure S8). Compound **2** demonstrates similar behavior: mass loss of ~17% up to ~175 °C associated with the solvent removal (calculated: 18% for 4 DMF) and degradation of the MOF starting at ~325 °C (Figure S9). For Compound **3**, since—according to the XRD and chemical analyses—it does not have any guest solvent molecules, only one step on the TG curve is observed to be related to the degradation of the framework at ~370 °C and higher (Figure S10).

**Figure 6:**
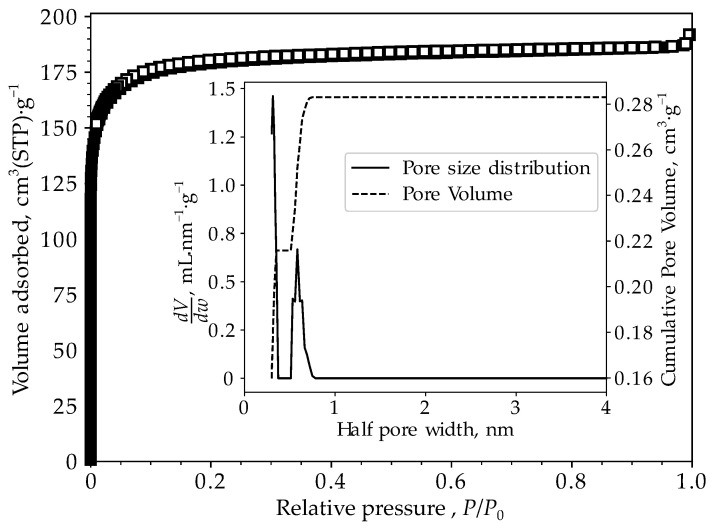
Nitrogen adsorption (filled squares) and desorption (open squares) isotherms at 77 K for Compound **1**. The inset shows the pore size distribution.

The permanent porosity of **1**, which has a 3D structure, was confirmed via the measurements of a N_2_ gas adsorption isotherm at 77 K. The as-synthesized crystals of **1** were activated by the solvent exchange (CH_2_Cl_2_) followed by a dynamic vacuum treatment at 180 °C for 6 h directly in a gas adsorption analyzer. The nitrogen adsorption-desorption isotherm plot at 77 K is represented in Figure 6 and belongs to the Ia isotherm type according to the official IUPAC classification [35], which is typical for microporous compounds with narrow slit pores. The measured pore volume of 0.288 cm^3^·g^–1^ (at p/p_0_ = 0.95, Table S3) matches the expected value (0.308 mL·g^–1^) estimated from the PLATON pore volume calculations, which confirms the structural integrity of **1** as well as the completeness of the framework activation. The PXRD patterns of 1 after activation and adsorption in comparison to the as-synthesized sample (Figure S16) also confirm its phase stability upon activation and adsorption. The calculated surface areas are 806 m^2^·g^–1^ (Langmuir model), 707 m^2^·g^–1^ (BET model), and 918 m^2^·g^–1^ (DFT model). The pore size distribution plot of **1**, calculated using the DFT model (Figure 6, inset), shows the presence of narrow pores with diameters less than ~1 nm, which is in a good agreement with the XRD structural data.

### 2.3. Gas Adsorption Studies

**Figure 7:**
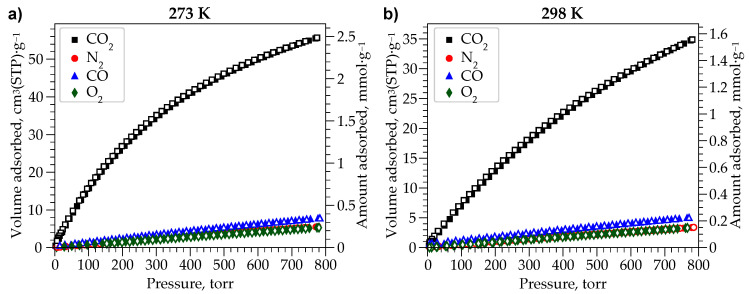
CO_2_, CO, N_2_, and O_2_ gas adsorption isotherms for Compound **1**: (**a**) at 273 K; (**b**) at 298 K.

**Figure 8:**
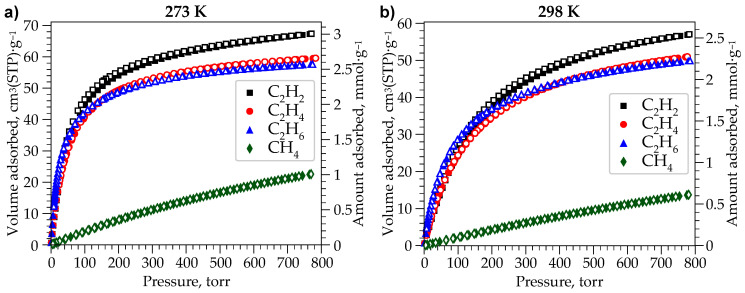
CH_4_, C_2_H_2_, C_2_H_4_, and C_2_H_6_ gas adsorption isotherms for Compound **1**: (**a**) at 273 K; (**b**) at 298 K.

The surface of the porous channels in **1** is lined by sulfur heteroatoms of the btdc^2–^ ligands. Such functional groups are known to enhance the adsorption uptake and selectivity of the corresponding MOFs towards small gas molecules, particularly CO_2_ [36,37]. The carbon dioxide is the most infamous anthropogenic pollutant causing global climate changes. Its acidic nature causes a chemical corrosion of valuable equipment unless the CO_2_ content in the gas mixture is reduced to a safe level before further processing. CO_2_ sequestration using adsorption technologies is one of the most efficient solutions for the purification of many industrially relevant gas mixtures, such as CO_2_/N_2_ (the main flue gas components), CO_2_/CH_4_ (the main components of biogas), and CO_2_/CO (production of steel). In this regard, a thorough investigation of the adsorption properties of **1** towards CO_2_ as well as other gases was performed within the current work. The adsorption–desorption isotherm data for “inorganic” (CO_2_, CO, N_2_, O_2_) and “organic” (CH_4_, C_2_H_2_, C_2_H_4_ and C_2_H_6_) gases at T = 273 K and 298 K were systematically measured. The corresponding isotherms are shown in Figure 7 and Figure 8; the gas uptakes in different units are summarized in Table 1.

The measured CO_2_, CH_4_, and C_2_ adsorption uptakes are comparable to those reported for other MOFs with modest porosity and specific surface area [38,39,40]. The calculated isosteric heats of adsorption at zero coverage Q_st_(0) for “inert” inorganic gases are typically low (12.1 kJ·mol^−1^ for CO, 13.6 kJ·mol^−1^ for O_2_, and 15.0 kJ·mol^−1^ for N_2_), whereas the adsorption heat for a polar CO_2_ molecule is somewhat higher, 25.3 kJ·mol^–1^ (Table S6, Figure S12). The adsorption heat for light hydrocarbons steadily increases from methane (19.4 kJ·mol^–1^) through acetylene (27.9 kJ·mol^–1^) and ethylene (28.2 kJ·mol^–1^) to ethane (30.8 kJ·mol^–1^) consistently with the increase in the number of atoms in the molecule capable of the interactions with the microporous surface. The isosteric heats of the gas adsorption for **1** are generally low confirming weak intermolecular interactions during the physical adsorption. From the practical point of view, such low adsorption heat values are considered an advantage since they reduce the energy costs in a desorption cycle.

The adsorption selectivity factors for the separation of binary gas mixtures were evaluated using three different methods: (i) as a ratio of the amount adsorbed; (ii) as a ratio of the corresponding Henry constants; and (iii) using ideal adsorbed solution theory (IAST) [41] calculations, which allowed an estimation of the selectivity factors for the different gas mixture compositions and total pressures. The results are summarized in Table 2 and in Figure S14. Among the “inorganic gases”, carbon dioxide adsorption is rather substantial (54.9 cm^3^·g^−1^ at 273 K and 34.0 cm^3^·g^−1^ at 298 K), suggesting a significant CO_2_/N_2_ adsorption selectivity, which was confirmed experimentally by the corresponding IAST adsorption selectivity factors (31.0 at 273 K and 19.1 at 298 K for the equimolar gas mixture at 1 bar). The calculated adsorption selectivity values are quite remarkable for a porous compound with no specific CO_2_ adsorption sites, such as coordinatively unsaturated metal sites (CUSs) or amine groups. Moreover, a low CO_2_ isosteric adsorption heat Q_st_(0) = 25.3 kJ·mol^–1^ minimizes parasite energy loss during the regeneration of the porous material in a practical gas separation process. Other than CO_2_/N_2_, promising IAST adsorption selectivity values at 298 K were also obtained for CO_2_/CO (17.0) and CO_2_/CH_4_ (3.6), which are comparable to other high-performing porous MOFs [42,43,44].

The adsorption uptakes for C_2_-hydrocarbons are very similar, suggesting no adsorption selectivity in their binary mixtures. At the same time, remarkable C_2_H_6_/CH_4_, C_2_H_4_/CH_4_, and C_2_H_2_/CH_4_ adsorption selectivity factors were calculated: 33.4, 24.8, and 29.3 at 273 K and 24.6, 17.7, and 19.1 at 298 K, respectively (1 bar, equimolar gas mixtures). By the combination C_2_H_6_/CH_4_ adsorption selectivity and the C_2_H_6_ adsorption capacity Compound **1** can be included in the top ten MOFs [45,46,47,48,49,50,51,52], and C_2_H_4_/CH_4_ and C_2_H_2_/CH_4_ adsorption selectivity factors are also high enough to consider **1** as a promising material for the adsorption separation of natural, shale and associated petroleum gas into individual components valuable for chemical industry. Moreover, the values of C_2_H_6_/CH_4_ adsorption selectivity become even higher as the content of C_2_H_6_ in the mixture shifts closer to the actual ethane composition in the natural gas, typically 5–10% (Table 2). Great CO_2_/N_2_, CO_2_/CO, and C_2_H_6_/CH_4_ adsorption selectivity potential and one of the lowest adsorption heats place the porous material **1** among the most promising materials for the practical sequestration of CO_2_ as well as efficient separation of mixtures of light hydrocarbons, which are highly demanding tasks for many environmental, industrial, and safety applications.

### 2.4. Vapor Phase Adsorption of Benzene and Cyclohexane

**Figure 9:**
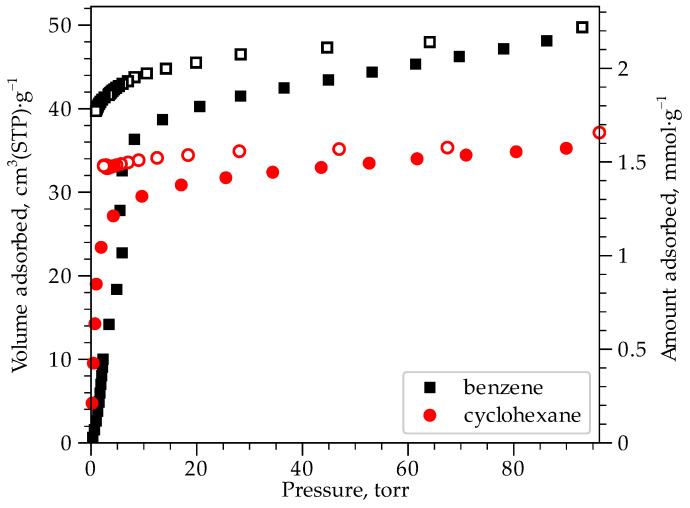
C_6_H_6_ and C_6_H_12_ vapor adsorption (filled symbols) and desorption (open symbols) isotherms for Compound **1** at 298 K.

We have also studied adsorption of benzene (C_6_H_6_) and cyclohexane (C_6_H_12_) vapors at T = 298 K on the activated **1**; the corresponding adsorption isotherms are presented in Figure 9. The adsorption uptake at saturation is 50.2 cm^3^·g^–1^ (2.24 mmol·g^–1^, or 14.9 wt.%) for C_6_H_6_ and 36.8 cm^3^·g^–1^ (1.64 mmol·g^–1^, or 12.1 wt.%) for C_6_H_12_. It is interesting that despite the fact that at high vapor pressures there is an obvious preference for benzene adsorption compared to cyclohexane (V_B_/V_CH_ = 1.36 at the saturation pressure), at low pressures cyclohexane adsorbs on **1** better than benzene, which is confirmed by the ratio of the Henry constants (K_B_/K_CH_ = 0.16, or K_CH_/K_B_ = 6.33, Figure S15), and this is a very rare phenomenon observed previously only in few works [53,54,55].

**Figure 10:**
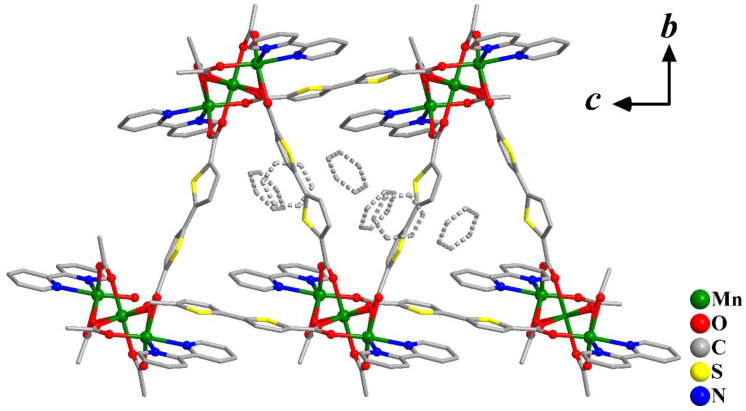
Fragment of the crystal structure of **1≅*2*C_6_H_6_**. The disordered positions of the guest benzene molecules are shown by a dashed line.

Preferable adsorption of benzene at high vapor pressures can be explained on the basis of the single crystal X-ray diffraction analysis of a **1** crystal immersed in pure benzene for several days (100% of pore saturation). According to X-ray crystallography data, benzene molecules are disordered over three positions with s.o.f. 0.365(2)/0.289(2)/0.346(2) (Figure 10). The total amount is two benzene molecules per formula unit of Framework **1**. A thorough analysis of the structure shows the existence of the relatively short host–guest interaction: C-H(benzene)···C(bpy) = 3.170–3.810 Å for the interactions of the first benzene molecule and a pyridyl ring, C-H(benzene)···C(btdc^2−^) = 2.996–3.275 Å for the interactions of the first benzene molecule and a thiophene ring of one btdc^2−^ ligand, and C-H(benzene)···C(btdc^2−^) = 3.197–3.910 Å for the interactions of the second benzene molecule and a thiophene ring of another btdc^2−^ ligand. Therefore, numerous van der Waals interactions are responsible for a preferable adsorption of benzene over cyclohexane on the activated **1** at 100% pore saturation.

### 2.5. Magnetic Properties of Compounds ***1***–***3***

**Figure 11:**
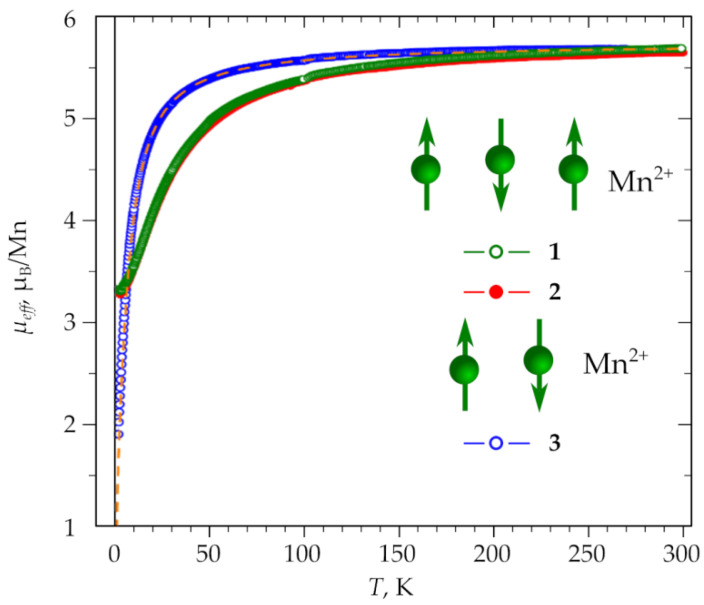
Temperature dependences of the effective magnetic moments per one Mn ion, μ_eff_, measured for **1**–**3**. Inset illustrates the magnetic ground states of the trinuclear (**1** and **2**) and binuclear (**3**) SBUs. The orange dashed line shows a fit to the data of the Mn^2+^-Mn^2+^ dimer model [56] with the intradimer exchange interaction J/*k*_B_ = 2.0 K.

Temperature dependences of the molar magnetic susceptibility χ were measured for **1**–**3** in the range 1.77–330 K at magnetic fields *H* up to 10 kOe under zero-field-cooled and field-cooled conditions (Figures S17a–S19a). All the compounds studied kept a paramagnetic state down to the lowest accessible temperature without any anomaly that could be attributed to long-range magnetic ordering or any sign of magnetothermal irreversibility associated with spin freezing. Data analysis has shown, however, that the paramagnetic part of the magnetic susceptibility, χ_p_(*T*), obtained by subtracting the diamagnetic contribution does not follow the conventional Curie–Weiss dependence χ_p_(*T*) = *N*_a_·μ_eff_^2^/(3·*k*_B_·(*T* − θ)) (Figures S17b–S19b). Instead, the χ_p_(*T*) curves demonstrate almost perfect canonical behavior of trinuclear (**1** and **2**) and binuclear (**3**) compounds with an antiferromagnetic (AF) coupling of the magnetic moments within magnetic clusters [56] and negligible interaction between them. This is not surprising given that the crystal structures of Compounds **1**, **2**, and **3** are indeed built of trinuclear and binuclear SBUs, respectively. As can be seen in Figure 11 (see also Figures S17b–S19b), the effective magnetic moments, μ_eff_, calculated for one manganese ion gradually decrease upon cooling, tending to saturate at a constant value of 3.25–3.31 μ_B_ in the case of Compounds **1** and **2**—built of trinuclear SBUs—or tending to zero in the case of binuclear Compound **3**. In the former case, the low-temperature values of μ_eff_ differ from the high-temperature ones of 5.65–5.69 μ_B_ by eactly √3 times, implying that the magnetic response of a trinuclear SBU is reduced at low temperatures by 3 times (proportionally to μ_eff_^2^), that is, to a response of a single ion. This is quite expected given that the AF interaction in a linear trimer should fix the relative orientation of magnetic moments in the ground state so that the resulting moment is equal to that of a single ion (inset in Figure 11). In turn, the binuclear Compound **3** demonstrates the effective magnetic moment tending to zero at low temperatures owing to the singlet ground state in dimers with AF exchange interaction between ions (inset in Figure 11).

In the high-temperature region, the effective magnetic moments of all Compounds **1–3** reach similar values of 5.65–5.69 μ_B_ per manganese ion (Figure 11), which are very close to the μ_eff_ ≈ 5.92 μ_B_ expected for isolated Mn^2+^ ions (S = 5/2, L = 0), especially if the AF interaction within dimers and trimers still slightly affecting the magnetic susceptibility at high *T* is taken into account. Given that Mn^2+^ ions have no orbital moments (L = 0), the effects associated with the contribution of orbital moments and with zero-field splitting that usually affect the magnetic susceptibility behavior are expected to be rather weak in the compounds studied. This justifies the above description where the temperature dependences of μ_eff_ were attributed solely to the AF interactions within trinuclear (**1** and **2**) and binuclear (**3**) SBUs.

An additional confirmation for the description based on AF dimers and trimers comes from the magnetic field dependences of the magnetization, *M*(*H*), measured for **1–3** at *T* = 1.77 K (Figures S17c–S19c). The *M*(*H*) curves for **1** and **2** can be well fitted—both the shape and magnitude—by a conventional expression based on the Brillouin function with S = 5/2, as expected for the ground state of AF Mn^2+^ trimers. In the case of **3**, the *M*(*H*) dependence is close to linear, and the magnetization stays below 1 μ_B_ per Mn^2+^ dimer at *H* = 10 kOe, in agreement with the singlet ground state of dimers.

To evaluate the magnitude of the AF exchange interaction in **3**, we have fitted the magnetic susceptibility data with the model of Mn^2+^-Mn^2+^ dimers [56]; a rather good fit has been obtained for J/*k*_B_ = 2.0 K (orange dashed line in Figure 11). Though this value appears small, this intradimer interaction is sufficient to govern the magnetic behavior of **3** and to cause its magnetic susceptibility to saturate (or pass through a maximum) at *T* < 2 K (Figure S19a). Fitting of the trimer’s magnetic behavior is a more difficult and less reliable procedure going beyond the scope of this work. Here, we simply point out that the AF exchange interactions in Compounds **1** and **2** have the same strength, which is quite expected due to the similar structure of both compounds based on trinuclear building blocks, and that this interaction is noticeably stronger than in the binuclear Compound **3**.

In the low-temperature region where Mn^2+^ trimers acquire their ground state resembling that of a single ion, one can use a conventional Curie–Weiss fitting to evaluate the strength of the interaction between the trimers. Such analysis for **1** and **2** has shown that the temperature dependence of the reversed magnetic susceptibility at low *T* goes exactly to the origin (Figures S17b and S18b), and the Weiss constant θ equals zero within the experimental accuracy (|θ| < 0.1 K), pointing to the negligible interaction between trinuclear SBUs located rather far from each other in the crystal structure.

## 3. Conclusions

Five new metal–organic frameworks based on Mn(II), 2,2′-bithiophen-5,5′-dicarboxylate (btdc^2–^) and chelating N-donor ligands ([Mn_3_(btdc)_3_(bpy)_2_]·4DMF (**1**), [Mn_3_(btdc)_3_(5,5′-dmbpy)_2_]·5DMF (**2**), [Mn(btdc)(4,4′-dmbpy)] (**3**), [Mn_2_(btdc)_2_(bpy)(dmf)]·0.5DMF (**4**), [Mn_2_(btdc)_2_(4,4′dmbpy)(dmf)]·DMF (**5**) (bpy = 2,2′-bipyridyl, 5,5′-dmbpy = 5,5′-dimethyl-2,2′-bipyridyl, 4,4′-dmbpy = 4,4′-dimethyl-2,2′-bipyridyl, dmf, DMF = N,N-dimethylformamide) were synthesized and characterized using a number of physical–chemical methods, such as powder X-ray diffraction, TG analysis, and IR spectroscopy. The influence of the bulkiness of the chelating N-donor ligand on the dimensionality and structure of the coordination polymer has been analyzed, and the decrease in the framework dimensionality as well as the secondary building unit’s nuclearity and connectivity has been observed. For the 3D coordination polymer **1**, the textural and gas adsorption properties have been studied, revealing noticeable ideal adsorbed solution theory (IAST) CO_2_/N_2_ and CO_2_/CO selectivity factors as well as a significant adsorption selectivity for binary C_2_-C_1_ hydrocarbons mixtures, which makes it possible to separate natural, shale, and associated petroleum gas into valuable individual components. The ability of Compound **1** to separate benzene and cyclohexane in a vapor phase has been also analyzed. The preferable adsorption of benzene by **1** has been explained by multiple van der Waals interactions between guest benzene molecules and the metal–organic host, as it was revealed by the XRD analysis of **1** immersed in pure benzene for several days (**1≅*2*C_6_H_6_**). It is interesting that at low vapor pressures, an inversed behavior of **1** with preferable adsorption of cyclohexane over benzene was observed. This is a very rare phenomenon. Moreover, magnetic properties (the temperature-dependent molar magnetic susceptibility, χ_p_(*T*), and effective magnetic moments, μ_eff_(*T*) as well as the field-dependent magnetization, *M*(*H*)) have been studied for Compounds **1**–**3**, revealing paramagnetic behavior consistent with their crystal structure.

## Figures and Tables

**Table 1:** Gas uptakes on **1** at 1 bar.

Gas	273 K			298 K		
	cm^3^·g^−1^	mmol·g^−1^	wt.%	cm^3^·g^−1^	mmol·g^−1^	wt.%
CO_2_	54.9	2.45	9.7	34.0	1.52	6.3
CO	7.6	0.34	0.9	4.9	0.22	0.6
N_2_	5.4	0.24	0.7	3.2	0.14	0.4
O_2_	5.1	0.23	0.7	3.2	0.14	0.5
CH_4_	22.2	0.99	1.6	13.3	0.59	0.9
C_2_H_2_	67.1	2.99	7.2	56.5	2.52	6.2
C_2_H_4_	59.3	2.65	6.9	50.6	2.26	6.0
C_2_H_6_	57.5	2.56	7.2	49.5	2.21	6.2

**Table 2:** Adsorption selectivity factors for separation of binary gas mixtures evaluated via different approaches.

Gas Mixture	273 K			298 K		
	V_1_/V_2_	K_H1_/K_H2_	IAST ^a^	V_1_/V_2_	K_H1_/K_H2_	IAST ^a^
CO_2_/N_2_	10.2	23.8	31.0	10.6	16.2	19.1
CO_2_/CO	7.2	8.1	25.7	6.9	5.0	17.0
CO_2_/CH_4_	2.5	4.2	4.8	2.6	3.4	3.6
C_2_H_6_/CH_4_	2.6	44.9	33.4 37.7 ^b^	3.7	29.5	24.6 27.2 ^b^
C_2_H_4_/CH_4_	2.7	24.6	24.8	3.8	17.8	17.7
C_2_H_2_/CH_4_	3.0	21.1	29.3	4.2	15.4	19.1

^a^ for the equimolar gas mixture composition at the total pressure of 1 bar; ^b^ C_2_H_6_:CH_4_ = 1:9 (*v*/*v*).

## Data Availability

The data presented in this study are available in Supplementary Materials. CCDC 2227366–2227371 contains the supplementary crystallographic data for this paper. These data can be obtained free of charge from The Cambridge Crystallographic Data Center at https://www.ccdc.cam.ac.uk/structures/ (accessed on 25 January 2023).

## Supplementary Materials

The following supporting information can be downloaded at: https://www.mdpi.com/article/10.3390/molecules28052139/s1, Table S1: Crystal data and structure refinement for **1**–**3**; Table S2: Crystal data and structure refinement for **4**, **5**, and **1≅2C_6_H_6_**; Figure S1: PXRD patterns of **1** (experimental—red, calculated from the single crystal X-ray diffraction data—black); Figure S2: PXRD patterns of **2** (experimental—red, calculated from the single crystal X-ray diffraction data—black); Figure S3: PXRD patterns of **3** (experimental—red, calculated from the single crystal X-ray diffraction data—black); Figure S4. Optical images of the crystals: (a) pure phase **1**; (b) pure phase **2**; (c) pure phase **3**; (d) mixture of Compounds **1** and **4**; (e) mixture of Compounds **2** and **5**; Figure S5: IR spectrum of **1**; Figure S6: IR spectrum of **2**; Figure S7: IR spectrum of **3**; Figure S8: TG curve of **1**; Figure S9: TG curve of **2**; Figure S10: TG curve of **3**; Table S3: The textural parameters of the porous structure of **1***;* Table S4: Virial coefficients *A_i_* and *B_j_* for gas adsorption isotherms at 273 K and 298 K on 1; Figure S11: Fits of isotherms by virial equation; Table S5: Henry constants for gas adsorption on 1 in mmol·g^−1^·bar^−1^ at 273 K and 298 K obtained by virial approach; Table S6: Zero coverage heats of adsorption in kJ/mol; Figure S12: Isosteric heats of gas adsorption on 1 calculated by a virial approach; Table S7: Fitted parameters for the adsorption isotherms on 1 at 273 K and 298 K and the corresponding Henry constants for comparison obtained by fitting and calculations performed using a virial approach, in brackets the deviations from a virial Henry constant are given; Figure S13: Fits of isotherms by an appropriate model; Figure S14: The prediction of an adsorption equilibrium by IAST (solid lines) and dependence of selectivity factors on a gas phase composition (dashed lines) as well as their pressure dependence for binary gas mixtures: (a) CO_2_/N_2_; (b) CO_2_/CO; (c) CO_2_/CH_4_; (d) C_2_H_6_/CH_4_; (e) C_2_H_4_/CH_4_; (f) C_2_H_2_/CH_4_; Figure S15: Fit of the initial (linear) parts of vapor adsorption isotherms by the Henry law, [k] = mmol·g^–1^·torr^–1^; Figure S16: PXRD patterns of **1**: as-synthesized (red), activated (blue), and after adsorption (purple); Figure S17: (a) Temperature dependences of the magnetic susceptibility *χ* of **1** measured at magnetic fields *H* = 1; 10 kOe. (b) Temperature dependences of the effective magnetic moment μ_eff_ and the reversed magnetic susceptibility 1/χ*_p_* for **1**. (c) Magnetic field dependence of the magnetization *M* measured for **1** at *T* = 1.77 K; Figure S18: (a) Temperature dependences of the magnetic susceptibility χ of **2** measured at magnetic fields *H* = 1; 10 kOe. (b) Temperature dependences of the effective magnetic moment μ_eff_ and the reversed magnetic susceptibility 1/χ*_p_* for **2**. (c) Magnetic field dependence of the magnetization *M* measured for **2** at *T* = 1.77 K; Figure S19: (a) Temperature dependences of the magnetic susceptibility χ of **3** measured at magnetic fields *H* = 1; 10 kOe. (b) Temperature dependences of the effective magnetic moment μ_eff_ and the reversed magnetic susceptibility 1/*χ_p_* for **3**. (c) Magnetic field dependence of the magnetization *M* measured for **3** at *T* = 1.77 K. References [57,58,59,60,61,62,63] are cited in the Supplementary Materials.
