# Supplementary material for: A Series of Metal–Organic Frameworks with 2,2′-Bipyridyl Derivatives: Synthesis vs. Structure Relationships, Adsorption, and Magnetic Studies
Source: Molecules. 2023 Feb 24;28(5):2139. doi: 10.3390/molecules28052139 (PMC10004071; doi:10.3390/molecules28052139)

checkCIF/PLATON report

Structure factors have been supplied for datablock(s) ssm1c

THIS REPORT IS FOR GUIDANCE ONLY. IF USED AS PART OF A REVIEW PROCEDURE FOR PUBLICATION, IT SHOULD NOT REPLACE THE EXPERTISE OF AN EXPERIENCED CRYSTALLOGRAPHIC REFEREE.

No syntax errors found. CIF dictionary Interpreting this report

Datablock: ssm1c

```
Bond precision:   C-C = 0.0038 A          Wavelength=0.71073

Cell:             a=10.9298(4)     b=25.5452(11)       c=13.0284(5)
                  alpha=90         beta=111.044(4)     gamma=90
Temperature:      150 K

                 Calculated                     Reported
Volume           3395.0(2)                      3395.0(2)
Space group      P 21/c                         P 21/c
Hall group       -P 2ybc                        -P 2ybc
                 C50 H28 Mn3 N4 O12 S6,
Moiety formula   3(C1.50 H3.50 N0.50 O0.50), ?
                 2(C1.50 H3.5
Sum formula      C62 H56 Mn3 N8 O16 S6          C62 H56 Mn3 N8 O16 S6
Mr               1526.33                        1526.32
Dx,g cm-3        1.493                          1.493
Z                2                              2
Mu (mm-1)        0.806                          0.806
F000             1566.0                         1566.0
F000'            1570.12
h,k,lmax         13,31,15                       13,31,15
Nref             6448                           6446
Tmin,Tmax        0.816,0.961                    0.874,1.000
Tmin'            0.696

Correction method= # Reported T Limits: Tmin=0.874 Tmax=1.000
AbsCorr = MULTI-SCAN

Data completeness= 1.000           Theta(max)= 25.680
```

R(reflections)= 0.0395(5490) wR2(reflections)= 0.1160(6446)

S = 1.041 Npar= 582

The following ALERTS were generated. Each ALERT has the format
test-name_ALERT_alert-type_alert-level.
Click on the hyperlinks for more details of the test.

Alert level C

PLAT906_ALERT_3_C Large K Value in the Analysis of Variance ...... 2.218 Check

---

Alert level G

```
PLAT002_ALERT_2_G Number of Distance or Angle Restraints on AtSite         34 Note
PLAT003_ALERT_2_G Number of Uiso or Uij Restrained non-H Atoms ...         33 Report
PLAT004_ALERT_5_G Polymeric Structure Found with Maximum Dimension          3 Info
PLAT174_ALERT_4_G The CIF-Embedded .res File Contains FLAT Records          2 Report
PLAT176_ALERT_4_G The CIF-Embedded .res File Contains SADI Records         10 Report
PLAT178_ALERT_4_G The CIF-Embedded .res File Contains SIMU Records          2 Report
PLAT232_ALERT_2_G Hirshfeld Test Diff (M-X)  Mn1      --N1       .        5.7 s.u.
PLAT300_ALERT_4_G Atom Site Occupancy of N2        Constrained at        0.5 Check
PLAT300_ALERT_4_G Atom Site Occupancy of N3        Constrained at        0.5 Check
PLAT300_ALERT_4_G Atom Site Occupancy of C106      Constrained at        0.5 Check
PLAT300_ALERT_4_G Atom Site Occupancy of C107      Constrained at        0.5 Check
PLAT300_ALERT_4_G Atom Site Occupancy of C108      Constrained at        0.5 Check
PLAT300_ALERT_4_G Atom Site Occupancy of C109      Constrained at        0.5 Check
PLAT300_ALERT_4_G Atom Site Occupancy of C110      Constrained at        0.5 Check
PLAT300_ALERT_4_G Atom Site Occupancy of C111      Constrained at        0.5 Check
PLAT300_ALERT_4_G Atom Site Occupancy of C112      Constrained at        0.5 Check
PLAT300_ALERT_4_G Atom Site Occupancy of C113      Constrained at        0.5 Check
PLAT300_ALERT_4_G Atom Site Occupancy of C114      Constrained at        0.5 Check
PLAT300_ALERT_4_G Atom Site Occupancy of C115      Constrained at        0.5 Check
PLAT300_ALERT_4_G Atom Site Occupancy of H107      Constrained at        0.5 Check
PLAT300_ALERT_4_G Atom Site Occupancy of H108      Constrained at        0.5 Check
PLAT300_ALERT_4_G Atom Site Occupancy of H109      Constrained at        0.5 Check
PLAT300_ALERT_4_G Atom Site Occupancy of H110      Constrained at        0.5 Check
PLAT300_ALERT_4_G Atom Site Occupancy of H112      Constrained at        0.5 Check
PLAT300_ALERT_4_G Atom Site Occupancy of H113      Constrained at        0.5 Check
PLAT300_ALERT_4_G Atom Site Occupancy of H114      Constrained at        0.5 Check
PLAT300_ALERT_4_G Atom Site Occupancy of H115      Constrained at        0.5 Check
PLAT300_ALERT_4_G Atom Site Occupancy of O1A       Constrained at        0.5 Check
PLAT300_ALERT_4_G Atom Site Occupancy of N1A       Constrained at        0.5 Check
PLAT300_ALERT_4_G Atom Site Occupancy of C11A      Constrained at        0.5 Check
PLAT300_ALERT_4_G Atom Site Occupancy of C12A      Constrained at        0.5 Check
PLAT300_ALERT_4_G Atom Site Occupancy of C13A      Constrained at        0.5 Check
PLAT300_ALERT_4_G Atom Site Occupancy of H11A      Constrained at        0.5 Check
PLAT300_ALERT_4_G Atom Site Occupancy of H12A      Constrained at        0.5 Check
PLAT300_ALERT_4_G Atom Site Occupancy of H12B      Constrained at        0.5 Check
PLAT300_ALERT_4_G Atom Site Occupancy of H12C      Constrained at        0.5 Check
PLAT300_ALERT_4_G Atom Site Occupancy of H13A      Constrained at        0.5 Check
PLAT300_ALERT_4_G Atom Site Occupancy of H13B      Constrained at        0.5 Check
PLAT300_ALERT_4_G Atom Site Occupancy of H13C      Constrained at        0.5 Check
PLAT300_ALERT_4_G Atom Site Occupancy of O1D       Constrained at        0.5 Check
PLAT300_ALERT_4_G Atom Site Occupancy of N1D       Constrained at        0.5 Check
```

```
PLAT300_ALERT_4_G Atom Site Occupancy of C11D       Constrained at        0.5 Check
PLAT300_ALERT_4_G Atom Site Occupancy of C12D       Constrained at        0.5 Check
PLAT300_ALERT_4_G Atom Site Occupancy of C13D       Constrained at        0.5 Check
PLAT300_ALERT_4_G Atom Site Occupancy of H11D       Constrained at        0.5 Check
PLAT300_ALERT_4_G Atom Site Occupancy of H12D       Constrained at        0.5 Check
PLAT300_ALERT_4_G Atom Site Occupancy of H12E       Constrained at        0.5 Check
PLAT300_ALERT_4_G Atom Site Occupancy of H12F       Constrained at        0.5 Check
PLAT300_ALERT_4_G Atom Site Occupancy of H13D       Constrained at        0.5 Check
PLAT300_ALERT_4_G Atom Site Occupancy of H13E       Constrained at        0.5 Check
PLAT300_ALERT_4_G Atom Site Occupancy of H13F       Constrained at        0.5 Check
PLAT300_ALERT_4_G Atom Site Occupancy of O2A        Constrained at        0.5 Check
PLAT300_ALERT_4_G Atom Site Occupancy of N2A        Constrained at        0.5 Check
PLAT300_ALERT_4_G Atom Site Occupancy of C21A       Constrained at        0.5 Check
PLAT300_ALERT_4_G Atom Site Occupancy of C22A       Constrained at        0.5 Check
PLAT300_ALERT_4_G Atom Site Occupancy of C23A       Constrained at        0.5 Check
PLAT300_ALERT_4_G Atom Site Occupancy of H21A       Constrained at        0.5 Check
PLAT300_ALERT_4_G Atom Site Occupancy of H22D       Constrained at        0.5 Check
PLAT300_ALERT_4_G Atom Site Occupancy of H22E       Constrained at        0.5 Check
PLAT300_ALERT_4_G Atom Site Occupancy of H22F       Constrained at        0.5 Check
PLAT300_ALERT_4_G Atom Site Occupancy of H23D       Constrained at        0.5 Check
PLAT300_ALERT_4_G Atom Site Occupancy of H23E       Constrained at        0.5 Check
PLAT300_ALERT_4_G Atom Site Occupancy of H23F       Constrained at        0.5 Check
PLAT300_ALERT_4_G Atom Site Occupancy of O2D        Constrained at        0.5 Check
PLAT300_ALERT_4_G Atom Site Occupancy of N2D        Constrained at        0.5 Check
PLAT300_ALERT_4_G Atom Site Occupancy of C21D       Constrained at        0.5 Check
PLAT300_ALERT_4_G Atom Site Occupancy of C22D       Constrained at        0.5 Check
PLAT300_ALERT_4_G Atom Site Occupancy of C23D       Constrained at        0.5 Check
PLAT300_ALERT_4_G Atom Site Occupancy of H21D       Constrained at        0.5 Check
PLAT300_ALERT_4_G Atom Site Occupancy of H22A       Constrained at        0.5 Check
PLAT300_ALERT_4_G Atom Site Occupancy of H22B       Constrained at        0.5 Check
PLAT300_ALERT_4_G Atom Site Occupancy of H22C       Constrained at        0.5 Check
PLAT300_ALERT_4_G Atom Site Occupancy of H23A       Constrained at        0.5 Check
PLAT300_ALERT_4_G Atom Site Occupancy of H23B       Constrained at        0.5 Check
PLAT300_ALERT_4_G Atom Site Occupancy of H23C       Constrained at        0.5 Check
PLAT301_ALERT_3_G Main Residue  Disorder .............(Resd  1  )         16% Note
PLAT302_ALERT_4_G Anion/Solvent/Minor-Residue Disorder (Resd  2  )       100% Note
PLAT302_ALERT_4_G Anion/Solvent/Minor-Residue Disorder (Resd  3  )       100% Note
PLAT302_ALERT_4_G Anion/Solvent/Minor-Residue Disorder (Resd  4  )       100% Note
PLAT302_ALERT_4_G Anion/Solvent/Minor-Residue Disorder (Resd  5  )       100% Note
PLAT410_ALERT_2_G Short Intra H...H Contact  H104     ..H107     .       2.11 Ang.
                                                    x,y,z  =      1_555 Check
PLAT794_ALERT_5_G Tentative Bond Valency for Mn2       (II)      .       2.14 Info
PLAT811_ALERT_5_G No ADDSYM Analysis: Too Many Excluded Atoms ....          ! Info
PLAT860_ALERT_3_G Number of Least-Squares Restraints ............         757 Note
PLAT910_ALERT_3_G Missing # of FCF Reflection(s) Below Theta(Min).          1 Note
PLAT933_ALERT_2_G Number of HKL-OMIT Records in Embedded .res File          1 Note
PLAT941_ALERT_3_G Average HKL Measurement Multiplicity ..........         2.6 Low
PLAT961_ALERT_5_G Dataset Contains no Negative Intensities .......     Please Check
PLAT978_ALERT_2_G Number C-C Bonds with Positive Residual Density.          7 Info
```

---

0 **ALERT level A** = Most likely a serious problem - resolve or explain
0 **ALERT level B** = A potentially serious problem, consider carefully
1 **ALERT level C** = Check. Ensure it is not caused by an omission or oversight
89 **ALERT level G** = General information/check it is not something unexpected

0 ALERT type 1 CIF construction/syntax error, inconsistent or missing data

```
 6 ALERT type 2 Indicator that the structure model may be wrong or deficient
 5 ALERT type 3 Indicator that the structure quality may be low
75 ALERT type 4 Improvement, methodology, query or suggestion
 4 ALERT type 5 Informative message, check
```

It is advisable to attempt to resolve as many as possible of the alerts in all categories. Often the minor alerts point to easily fixed oversights, errors and omissions in your CIF or refinement strategy, so attention to these fine details can be worthwhile. In order to resolve some of the more serious problems it may be necessary to carry out additional measurements or structure refinements. However, the purpose of your study may justify the reported deviations and the more serious of these should normally be commented upon in the discussion or experimental section of a paper or in the "special_details" fields of the CIF. checkCIF was carefully designed to identify outliers and unusual parameters, but every test has its limitations and alerts that are not important in a particular case may appear. Conversely, the absence of alerts does not guarantee there are no aspects of the results needing attention. It is up to the individual to critically assess their own results and, if necessary, seek expert advice.

Publication of your CIF in IUCr journals

A basic structural check has been run on your CIF. These basic checks will be run on all CIFs submitted for publication in IUCr journals (*Acta Crystallographica*, *Journal of Applied Crystallography*, *Journal of Synchrotron Radiation*); however, if you intend to submit to *Acta Crystallographica Section C* or *E* or *IUCrData*, you should make sure that full publication checks are run on the final version of your CIF prior to submission.

Publication of your CIF in other journals

Please refer to the *Notes for Authors* of the relevant journal for any special instructions relating to CIF submission.

PLATON version of 18/05/2022; check.def file version of 17/05/2022

Datablock ssm1c - ellipsoid plot

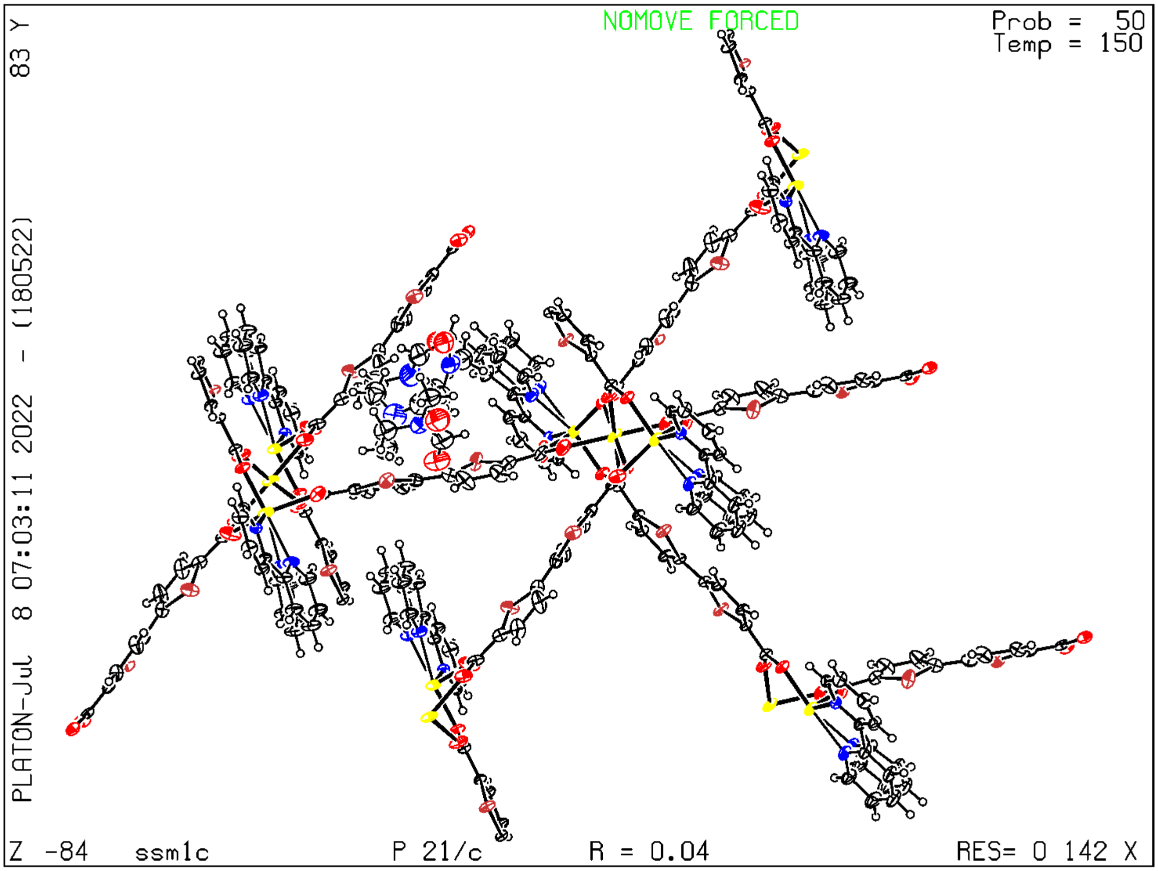

Supplement: Supplementary file 1 [file molecules-28-02139-s001.zip › 1-checkcif.pdf]
