# Supplementary material for: A Series of Metal–Organic Frameworks with 2,2′-Bipyridyl Derivatives: Synthesis vs. Structure Relationships, Adsorption, and Magnetic Studies
Source: Molecules. 2023 Feb 24;28(5):2139. doi: 10.3390/molecules28052139 (PMC10004071; doi:10.3390/molecules28052139)

checkCIF/PLATON report

Structure factors have been supplied for datablock(s) SSM-7C

THIS REPORT IS FOR GUIDANCE ONLY. IF USED AS PART OF A REVIEW PROCEDURE FOR PUBLICATION, IT SHOULD NOT REPLACE THE EXPERTISE OF AN EXPERIENCED CRYSTALLOGRAPHIC REFEREE.

No syntax errors found.   CIF dictionary   Interpreting this report

Datablock: SSM-7C

Bond precision: C-C = 0.0023 A   Wavelength=0.71073

Cell:	a=16.4304(5)	b=25.1354(9)	c=18.4058(5)
	alpha=90	beta=94.705(3)	gamma=90
Temperature:	150 K		

	Calculated	Reported
Volume	7575.7(4)	7575.7(4)
Space group	P 21/n	P 21/n
Hall group	-P 2yn	-P 2yn
Moiety formula	C54 H36 Mn3 N4 O12 S6, 5(C3 H7 N O)	?
Sum formula	C69 H71 Mn3 N9 O17 S6	C69 H71 Mn3 N9 O17 S6
Mr	1655.53	1655.52
Dx,g cm-3	1.452	1.452
Z	4	4
Mu (mm-1)	0.730	0.730
F000	3420.0	3420.0
F000’	3428.34	
h,k,lmax	22,34,25	21,33,24
Nref	20250	16855
Tmin,Tmax	0.832,0.930	0.875,1.000
Tmin’	0.609	

Correction method= # Reported T Limits: Tmin=0.875 Tmax=1.000
AbsCorr = MULTI-SCAN

Data completeness= 0.832   Theta(max)= 29.057

R(reflections)= 0.0323(13294)   wR2(reflections)= 0.0840(16855)

S = 1.046   Npar= 1052

The following ALERTS were generated. Each ALERT has the format
test-name_ALERT_alert-type_alert-level.
Click on the hyperlinks for more details of the test.

Alert level C

```
PLAT220_ALERT_2_C NonSolvent   Resd 1  C   Ueq(max)/Ueq(min) Range        3.4 Ratio
PLAT244_ALERT_4_C Low    'Solvent' Ueq as Compared to Neighbors of        N3D Check
PLAT244_ALERT_4_C Low    'Solvent' Ueq as Compared to Neighbors of        N1D Check
PLAT244_ALERT_4_C Low    'Solvent' Ueq as Compared to Neighbors of        N2D Check
PLAT906_ALERT_3_C Large K Value in the Analysis of Variance ......      3.392 Check
PLAT910_ALERT_3_C Missing # of FCF Reflection(s) Below Theta(Min).          8 Note
```

Alert level G

```
PLAT002_ALERT_2_G Number of Distance or Angle Restraints on AtSite         10 Note
PLAT003_ALERT_2_G Number of Uiso or Uij Restrained non-H Atoms ...         33 Report
PLAT004_ALERT_5_G Polymeric Structure Found with Maximum Dimension          2 Info
PLAT063_ALERT_4_G Crystal Size Possibly too Large for Beam Size ..       0.68 mm
PLAT176_ALERT_4_G The CIF-Embedded .res File Contains SADI Records          4 Report
PLAT178_ALERT_4_G The CIF-Embedded .res File Contains SIMU Records          4 Report
PLAT302_ALERT_4_G Anion/Solvent/Minor-Residue Disorder (Resd  2  )        80% Note
PLAT302_ALERT_4_G Anion/Solvent/Minor-Residue Disorder (Resd  3  )        80% Note
PLAT302_ALERT_4_G Anion/Solvent/Minor-Residue Disorder (Resd  6  )       100% Note
PLAT302_ALERT_4_G Anion/Solvent/Minor-Residue Disorder (Resd  7  )       100% Note
PLAT794_ALERT_5_G Tentative Bond Valency for Mn1       (II)      .       2.07 Info
PLAT794_ALERT_5_G Tentative Bond Valency for Mn2       (II)      .       2.10 Info
PLAT794_ALERT_5_G Tentative Bond Valency for Mn3       (II)      .       2.09 Info
PLAT860_ALERT_3_G Number of Least-Squares Restraints .............        301 Note
PLAT912_ALERT_4_G Missing # of FCF Reflections Above STh/L=  0.600       3301 Note
PLAT941_ALERT_3_G Average HKL Measurement Multiplicity ...........        2.4 Low
PLAT961_ALERT_5_G Dataset Contains no Negative Intensities .......     Please Check
PLAT978_ALERT_2_G Number C-C Bonds with Positive Residual Density.         10 Info
```

---

0 **ALERT level A** = Most likely a serious problem - resolve or explain
0 **ALERT level B** = A potentially serious problem, consider carefully
6 **ALERT level C** = Check. Ensure it is not caused by an omission or oversight
18 **ALERT level G** = General information/check it is not something unexpected

0 ALERT type 1 CIF construction/syntax error, inconsistent or missing data
4 ALERT type 2 Indicator that the structure model may be wrong or deficient
4 ALERT type 3 Indicator that the structure quality may be low
11 ALERT type 4 Improvement, methodology, query or suggestion
5 ALERT type 5 Informative message, check

It is advisable to attempt to resolve as many as possible of the alerts in all categories. Often the minor alerts point to easily fixed oversights, errors and omissions in your CIF or refinement strategy, so attention to these fine details can be worthwhile. In order to resolve some of the more serious problems it may be necessary to carry out additional measurements or structure refinements. However, the purpose of your study may justify the reported deviations and the more serious of these should normally be commented upon in the discussion or experimental section of a paper or in the "special_details" fields of the CIF. checkCIF was carefully designed to identify outliers and unusual parameters, but every test has its limitations and alerts that are not important in a particular case may appear. Conversely, the absence of alerts does not guarantee there are no aspects of the results needing attention. It is up to the individual to critically assess their own results and, if necessary, seek expert advice.

Publication of your CIF in IUCr journals

A basic structural check has been run on your CIF. These basic checks will be run on all CIFs submitted for publication in IUCr journals (*Acta Crystallographica*, *Journal of Applied Crystallography*, *Journal of Synchrotron Radiation*); however, if you intend to submit to *Acta Crystallographica Section C* or *E* or *IUCrData*, you should make sure that full publication checks are run on the final version of your CIF prior to submission.

Publication of your CIF in other journals

Please refer to the *Notes for Authors* of the relevant journal for any special instructions relating to CIF submission.

PLATON version of 22/03/2021; check.def file version of 19/03/2021

Datablock SSM-7C - ellipsoid plot

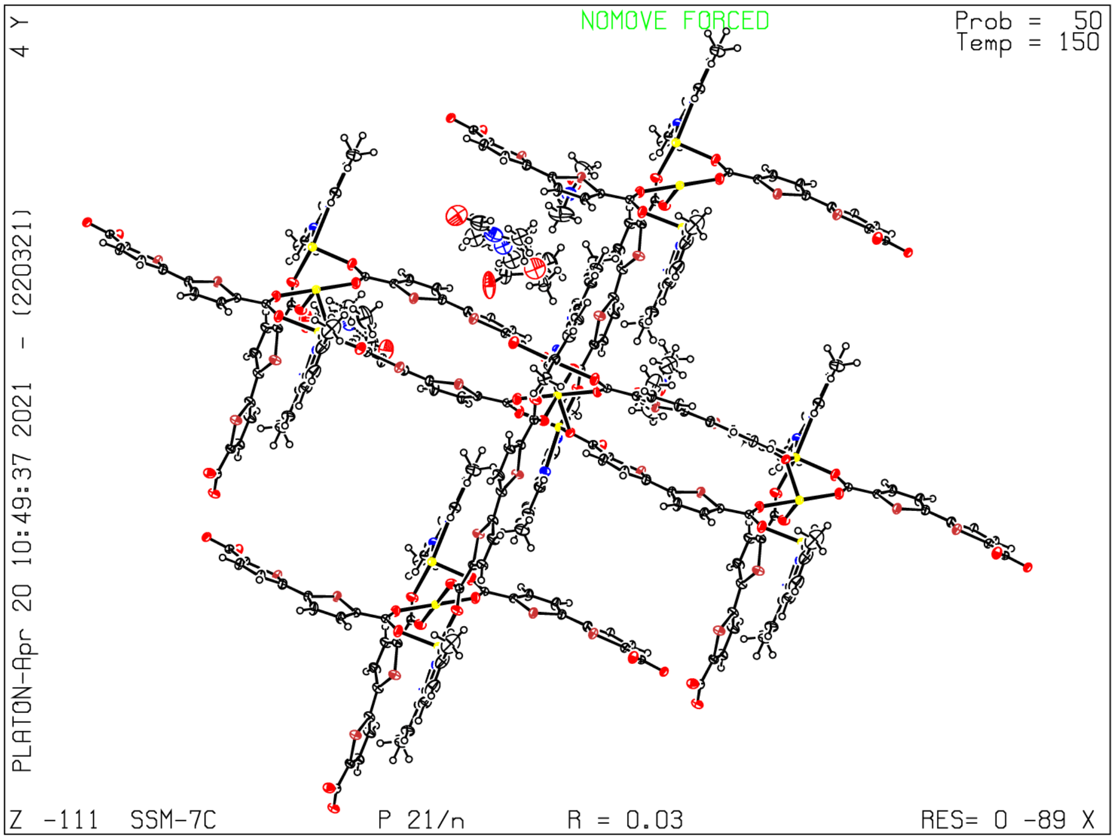

Supplement: Supplementary file 1 [file molecules-28-02139-s001.zip › 2-checkcif.pdf]
