# Supplementary material for: A Series of Metal–Organic Frameworks with 2,2′-Bipyridyl Derivatives: Synthesis vs. Structure Relationships, Adsorption, and Magnetic Studies
Source: Molecules. 2023 Feb 24;28(5):2139. doi: 10.3390/molecules28052139 (PMC10004071; doi:10.3390/molecules28052139)

checkCIF/PLATON report

Structure factors have been supplied for datablock(s) SSM-6C

THIS REPORT IS FOR GUIDANCE ONLY. IF USED AS PART OF A REVIEW PROCEDURE FOR PUBLICATION, IT SHOULD NOT REPLACE THE EXPERTISE OF AN EXPERIENCED CRYSTALLOGRAPHIC REFEREE.

No syntax errors found.   CIF dictionary   Interpreting this report

Datablock: SSM-6C

```
Bond precision:  C-C = 0.0040 A          Wavelength=0.71073

Cell:           a=10.5007(5)       b=10.5738(6)     c=11.0290(5)
                alpha=102.277(4)   beta=91.709(4)   gamma=117.108(5)
Temperature:    140 K

                Calculated                 Reported
Volume          1053.76(11)                1053.76(10)
Space group     P -1                       P -1
Hall group      -P 1                       -P 1
Moiety formula  C22 H16 Mn N2 O4 S2        ?
Sum formula     C22 H16 Mn N2 O4 S2        C22 H16 Mn N2 O4 S2
Mr              491.43                     491.43
Dx,g cm-3       1.549                      1.549
Z               2                          2
Mu (mm-1)       0.857                      0.857
F000            502.0                      502.0
F000'           503.35
h,k,lmax        14,14,15                   13,14,13
Nref            5621                       4607
Tmin,Tmax       0.879,0.942                0.982,1.000
Tmin'           0.879

Correction method= # Reported T Limits: Tmin=0.982 Tmax=1.000
AbsCorr = MULTI-SCAN

Data completeness= 0.820          Theta(max)= 29.041

R(reflections)= 0.0384( 3827)     wR2(reflections)= 0.0936( 4607)

S = 1.046                  Npar= 282
```

The following ALERTS were generated. Each ALERT has the format
test-name_ALERT_alert-type_alert-level.
Click on the hyperlinks for more details of the test.

Alert level C

```
PLAT094_ALERT_2_C Ratio of Maximum / Minimum Residual Density ....       3.27 Report
PLAT906_ALERT_3_C Large K Value in the Analysis of Variance ......      2.592 Check
```

Alert level G

```
PLAT004_ALERT_5_G Polymeric Structure Found with Maximum Dimension          2 Info
PLAT232_ALERT_2_G Hirshfeld Test Diff (M-X)  Mn1      --O22      .        9.2 s.u.
PLAT232_ALERT_2_G Hirshfeld Test Diff (M-X)  Mn1      --O11_b    .        8.9 s.u.
PLAT232_ALERT_2_G Hirshfeld Test Diff (M-X)  Mn1      --O12_b    .        6.9 s.u.
PLAT794_ALERT_5_G Tentative Bond Valency for Mn1       (II)      .       1.99 Info
PLAT910_ALERT_3_G Missing # of FCF Reflection(s) Below Theta(Min).          1 Note
PLAT912_ALERT_4_G Missing # of FCF Reflections Above STh/L=  0.600        909 Note
PLAT941_ALERT_3_G Average HKL Measurement Multiplicity ...........        1.7 Low
PLAT952_ALERT_5_G Calculated (ThMax) and CIF-Reported Lmax Differ           2 Units
PLAT958_ALERT_1_G Calculated (ThMax) and Actual (FCF) Lmax Differ           2 Units
PLAT961_ALERT_5_G Dataset Contains no Negative Intensities .......     Please Check
PLAT978_ALERT_2_G Number C-C Bonds with Positive Residual Density.          2 Info
PLAT992_ALERT_5_G Repd & Actual _reflns_number_gt Values Differ by          2 Check
```

---

0 **ALERT level A** = Most likely a serious problem - resolve or explain
0 **ALERT level B** = A potentially serious problem, consider carefully
2 **ALERT level C** = Check. Ensure it is not caused by an omission or oversight
13 **ALERT level G** = General information/check it is not something unexpected

1 ALERT type 1 CIF construction/syntax error, inconsistent or missing data
5 ALERT type 2 Indicator that the structure model may be wrong or deficient
3 ALERT type 3 Indicator that the structure quality may be low
1 ALERT type 4 Improvement, methodology, query or suggestion
5 ALERT type 5 Informative message, check

It is advisable to attempt to resolve as many as possible of the alerts in all categories. Often the minor alerts point to easily fixed oversights, errors and omissions in your CIF or refinement strategy, so attention to these fine details can be worthwhile. In order to resolve some of the more serious problems it may be necessary to carry out additional measurements or structure refinements. However, the purpose of your study may justify the reported deviations and the more serious of these should normally be commented upon in the discussion or experimental section of a paper or in the "special_details" fields of the CIF. checkCIF was carefully designed to identify outliers and unusual parameters, but every test has its limitations and alerts that are not important in a particular case may appear. Conversely, the absence of alerts does not guarantee there are no aspects of the results needing attention. It is up to the individual to critically assess their own results and, if necessary, seek expert advice.

Publication of your CIF in IUCr journals

A basic structural check has been run on your CIF. These basic checks will be run on all CIFs submitted for publication in IUCr journals (*Acta Crystallographica*, *Journal of Applied Crystallography*, *Journal of Synchrotron Radiation*); however, if you intend to submit to *Acta Crystallographica Section C* or *E* or *IUCrData*, you should make sure that full publication checks are run on the final version of your CIF prior to submission.

Publication of your CIF in other journals

Please refer to the *Notes for Authors* of the relevant journal for any special instructions relating to CIF submission.

PLATON version of 22/03/2021; check.def file version of 19/03/2021

Datablock SSM-6C - ellipsoid plot

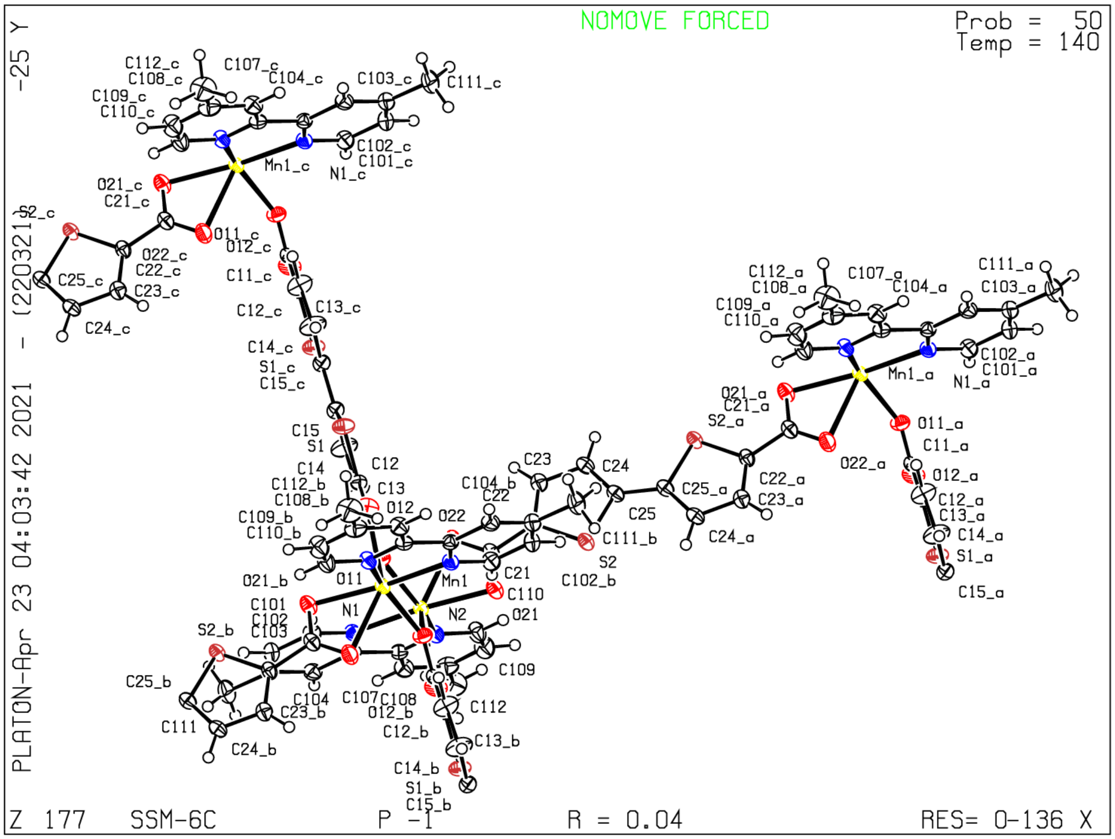

Supplement: Supplementary file 1 [file molecules-28-02139-s001.zip › 3-checkcif.pdf]
