# Supplementary material for: A Series of Metal–Organic Frameworks with 2,2′-Bipyridyl Derivatives: Synthesis vs. Structure Relationships, Adsorption, and Magnetic Studies
Source: Molecules. 2023 Feb 24;28(5):2139. doi: 10.3390/molecules28052139 (PMC10004071; doi:10.3390/molecules28052139)

checkCIF/PLATON report

Structure factors have been supplied for datablock(s) ssm-1ab

THIS REPORT IS FOR GUIDANCE ONLY. IF USED AS PART OF A REVIEW PROCEDURE FOR PUBLICATION, IT SHOULD NOT REPLACE THE EXPERTISE OF AN EXPERIENCED CRYSTALLOGRAPHIC REFEREE.

No syntax errors found. CIF dictionary Interpreting this report

Datablock: ssm-1ab

Bond precision:	C-C = 0.0056 A		Wavelength=0.79313
Cell:	a=16.206(3)	b=11.501(4)	c=22.561(3)
	alpha=90	beta=107.876(6)	gamma=90
Temperature:	100 K		

	Calculated	Reported
Volume	4002.0(17)	4002.0(17)
Space group	P 2/c	P 2/c
Hall group	-P 2yc	-P 2yc
Moiety formula	4(C33 H23 Mn2 N3 O9 S4), 5(C0.75 H1.75 N0.25 O0.25)	?
Sum formula	C138 H106 Mn8 N14 O38 S16	C34.50 H26.50 Mn2 N3.50 O9.50 S4
Mr	3520.85	880.21
Dx,g cm-3	1.461	1.461
Z	1	4
Mu (mm-1)	1.203	1.203
F000	1792.0	1792.0
F000'	1798.20	
h,k,lmax	19,13,27	18,13,27
Nref	7328	7148
Tmin,Tmax	0.917,0.942	0.845,1.000
Tmin'	0.887	

Correction method= # Reported T Limits: Tmin=0.845 Tmax=1.000
AbsCorr = MULTI-SCAN

Data completeness= 0.975 Theta(max)= 28.539

R(reflections)= 0.0516(6346)

wR2(reflections)= 0.1533(7148)

S = 1.028 Npar= 689

The following ALERTS were generated. Each ALERT has the format
test-name_ALERT_alert-type_alert-level.
Click on the hyperlinks for more details of the test.

Alert level C

```
PLAT029_ALERT_3_C _diffrn_measured_fraction_theta_full value Low .      0.976 Why?
PLAT220_ALERT_2_C NonSolvent   Resd 1  C   Ueq(max)/Ueq(min) Range        3.7 Ratio
PLAT220_ALERT_2_C NonSolvent   Resd 1  N   Ueq(max)/Ueq(min) Range        3.3 Ratio
PLAT241_ALERT_2_C High   'MainMol' Ueq as Compared to Neighbors of        C24 Check
PLAT242_ALERT_2_C Low    'MainMol' Ueq as Compared to Neighbors of        Mn2 Check
PLAT906_ALERT_3_C Large K Value in the Analysis of Variance ......      2.295 Check
PLAT911_ALERT_3_C Missing FCF Refl Between Thmin & STh/L=    0.600        151 Report
PLAT913_ALERT_3_C Missing # of Very Strong Reflections in FCF ....          9 Note
```

Alert level G

```
ABSMU01_ALERT_1_G  Calculation of _exptl_absorpt_correction_mu
                not performed for this radiation type.
PLAT002_ALERT_2_G Number of Distance or Angle Restraints on AtSite         31 Note
PLAT003_ALERT_2_G Number of Uiso or Uij Restrained non-H Atoms ...         35 Report
PLAT004_ALERT_5_G Polymeric Structure Found with Maximum Dimension          2 Info
PLAT045_ALERT_1_G Calculated and Reported Z Differ by a Factor ...       0.25 Check
PLAT083_ALERT_2_G SHELXL Second Parameter in WGHT  Unusually Large       6.43 Why ?
PLAT092_ALERT_4_G Check: Wavelength Given is not Cu,Ga,Mo,Ag,In Ka    0.79313 Ang.
PLAT172_ALERT_4_G The CIF-Embedded .res File Contains DFIX Records          4 Report
PLAT174_ALERT_4_G The CIF-Embedded .res File Contains FLAT Records          6 Report
PLAT176_ALERT_4_G The CIF-Embedded .res File Contains SADI Records          4 Report
PLAT178_ALERT_4_G The CIF-Embedded .res File Contains SIMU Records          4 Report
PLAT186_ALERT_4_G The CIF-Embedded .res File Contains ISOR Records          1 Report
PLAT300_ALERT_4_G Atom Site Occupancy of O1D       Constrained at        0.25 Check
PLAT300_ALERT_4_G Atom Site Occupancy of O2D       Constrained at        0.25 Check
PLAT300_ALERT_4_G Atom Site Occupancy of O3D       Constrained at        0.25 Check
PLAT300_ALERT_4_G Atom Site Occupancy of O4D       Constrained at        0.25 Check
PLAT300_ALERT_4_G Atom Site Occupancy of N1D       Constrained at        0.25 Check
PLAT300_ALERT_4_G Atom Site Occupancy of N2D       Constrained at        0.25 Check
PLAT300_ALERT_4_G Atom Site Occupancy of N3D       Constrained at        0.25 Check
PLAT300_ALERT_4_G Atom Site Occupancy of N4D       Constrained at        0.25 Check
PLAT300_ALERT_4_G Atom Site Occupancy of C11D      Constrained at        0.25 Check
PLAT300_ALERT_4_G Atom Site Occupancy of C12D      Constrained at        0.25 Check
PLAT300_ALERT_4_G Atom Site Occupancy of C13D      Constrained at        0.25 Check
PLAT300_ALERT_4_G Atom Site Occupancy of C21D      Constrained at        0.25 Check
PLAT300_ALERT_4_G Atom Site Occupancy of C22D      Constrained at        0.25 Check
PLAT300_ALERT_4_G Atom Site Occupancy of C23D      Constrained at        0.25 Check
PLAT300_ALERT_4_G Atom Site Occupancy of C31D      Constrained at        0.25 Check
PLAT300_ALERT_4_G Atom Site Occupancy of C32D      Constrained at        0.25 Check
PLAT300_ALERT_4_G Atom Site Occupancy of C33D      Constrained at        0.25 Check
PLAT300_ALERT_4_G Atom Site Occupancy of C41D      Constrained at        0.25 Check
PLAT300_ALERT_4_G Atom Site Occupancy of C42D      Constrained at        0.25 Check
PLAT300_ALERT_4_G Atom Site Occupancy of C43D      Constrained at        0.25 Check
PLAT300_ALERT_4_G Atom Site Occupancy of H11D      Constrained at        0.25 Check
```

```
PLAT300_ALERT_4_G Atom Site Occupancy of H12A       Constrained at       0.25 Check
PLAT300_ALERT_4_G Atom Site Occupancy of H12B       Constrained at       0.25 Check
PLAT300_ALERT_4_G Atom Site Occupancy of H12C       Constrained at       0.25 Check
PLAT300_ALERT_4_G Atom Site Occupancy of H13A       Constrained at       0.25 Check
PLAT300_ALERT_4_G Atom Site Occupancy of H13B       Constrained at       0.25 Check
PLAT300_ALERT_4_G Atom Site Occupancy of H13C       Constrained at       0.25 Check
PLAT300_ALERT_4_G Atom Site Occupancy of H21D       Constrained at       0.25 Check
PLAT300_ALERT_4_G Atom Site Occupancy of H22A       Constrained at       0.25 Check
PLAT300_ALERT_4_G Atom Site Occupancy of H22B       Constrained at       0.25 Check
PLAT300_ALERT_4_G Atom Site Occupancy of H22C       Constrained at       0.25 Check
PLAT300_ALERT_4_G Atom Site Occupancy of H23A       Constrained at       0.25 Check
PLAT300_ALERT_4_G Atom Site Occupancy of H23B       Constrained at       0.25 Check
PLAT300_ALERT_4_G Atom Site Occupancy of H23C       Constrained at       0.25 Check
PLAT300_ALERT_4_G Atom Site Occupancy of H31D       Constrained at       0.25 Check
PLAT300_ALERT_4_G Atom Site Occupancy of H32A       Constrained at       0.25 Check
PLAT300_ALERT_4_G Atom Site Occupancy of H32B       Constrained at       0.25 Check
PLAT300_ALERT_4_G Atom Site Occupancy of H32C       Constrained at       0.25 Check
PLAT300_ALERT_4_G Atom Site Occupancy of H33A       Constrained at       0.25 Check
PLAT300_ALERT_4_G Atom Site Occupancy of H33B       Constrained at       0.25 Check
PLAT300_ALERT_4_G Atom Site Occupancy of H33C       Constrained at       0.25 Check
PLAT300_ALERT_4_G Atom Site Occupancy of H41D       Constrained at       0.25 Check
PLAT300_ALERT_4_G Atom Site Occupancy of H42A       Constrained at       0.25 Check
PLAT300_ALERT_4_G Atom Site Occupancy of H42B       Constrained at       0.25 Check
PLAT300_ALERT_4_G Atom Site Occupancy of H42C       Constrained at       0.25 Check
PLAT300_ALERT_4_G Atom Site Occupancy of H43A       Constrained at       0.25 Check
PLAT300_ALERT_4_G Atom Site Occupancy of H43B       Constrained at       0.25 Check
PLAT300_ALERT_4_G Atom Site Occupancy of H43C       Constrained at       0.25 Check
PLAT300_ALERT_4_G Atom Site Occupancy of O5D        Constrained at       0.25 Check
PLAT300_ALERT_4_G Atom Site Occupancy of N5D        Constrained at       0.25 Check
PLAT300_ALERT_4_G Atom Site Occupancy of C51D       Constrained at       0.25 Check
PLAT300_ALERT_4_G Atom Site Occupancy of C52D       Constrained at       0.25 Check
PLAT300_ALERT_4_G Atom Site Occupancy of C53D       Constrained at       0.25 Check
PLAT300_ALERT_4_G Atom Site Occupancy of H51D       Constrained at       0.25 Check
PLAT300_ALERT_4_G Atom Site Occupancy of H52A       Constrained at       0.25 Check
PLAT300_ALERT_4_G Atom Site Occupancy of H52B       Constrained at       0.25 Check
PLAT300_ALERT_4_G Atom Site Occupancy of H52C       Constrained at       0.25 Check
PLAT300_ALERT_4_G Atom Site Occupancy of H53A       Constrained at       0.25 Check
PLAT300_ALERT_4_G Atom Site Occupancy of H53B       Constrained at       0.25 Check
PLAT300_ALERT_4_G Atom Site Occupancy of H53C       Constrained at       0.25 Check
PLAT300_ALERT_4_G Atom Site Occupancy of O6D        Constrained at       0.25 Check
PLAT300_ALERT_4_G Atom Site Occupancy of N6D        Constrained at       0.25 Check
PLAT300_ALERT_4_G Atom Site Occupancy of C61D       Constrained at       0.25 Check
PLAT300_ALERT_4_G Atom Site Occupancy of C62D       Constrained at       0.25 Check
PLAT300_ALERT_4_G Atom Site Occupancy of C63D       Constrained at       0.25 Check
PLAT300_ALERT_4_G Atom Site Occupancy of H61D       Constrained at       0.25 Check
PLAT300_ALERT_4_G Atom Site Occupancy of H62A       Constrained at       0.25 Check
PLAT300_ALERT_4_G Atom Site Occupancy of H62B       Constrained at       0.25 Check
PLAT300_ALERT_4_G Atom Site Occupancy of H62C       Constrained at       0.25 Check
PLAT300_ALERT_4_G Atom Site Occupancy of H63A       Constrained at       0.25 Check
PLAT300_ALERT_4_G Atom Site Occupancy of H63B       Constrained at       0.25 Check
PLAT300_ALERT_4_G Atom Site Occupancy of H63C       Constrained at       0.25 Check
PLAT301_ALERT_3_G Main Residue  Disorder .............(Resd  1  )        10% Note
PLAT302_ALERT_4_G Anion/Solvent/Minor-Residue Disorder (Resd  2  )       100% Note
PLAT302_ALERT_4_G Anion/Solvent/Minor-Residue Disorder (Resd  3  )       100% Note
PLAT411_ALERT_2_G Short Inter H...H Contact  H24      ..H61D     .       1.96 Ang.
                                               1-x,1-y,1-z  =      3_666 Check
PLAT789_ALERT_4_G Atoms with Negative _atom_site_disorder_group  #         24 Check
```

```
PLAT794_ALERT_5_G Tentative Bond Valency for Mn1       (II)      .       2.01 Info
PLAT860_ALERT_3_G Number of Least-Squares Restraints ............        546 Note
PLAT933_ALERT_2_G Number of OMIT Records in Embedded .res File ...        15 Note
PLAT961_ALERT_5_G Dataset Contains no Negative Intensities .......    Please Check
PLAT978_ALERT_2_G Number C-C Bonds with Positive Residual Density.         1 Info
PLAT984_ALERT_1_G The Mn-f'=    0.3578 Deviates from the B&C-Value     0.3558 Check
PLAT984_ALERT_1_G The  S-f'=    0.1503 Deviates from the B&C-Value     0.1482 Check
PLAT985_ALERT_1_G The Mn-f"=    0.8993 Deviates from the B&C-Value     0.8956 Check
PLAT985_ALERT_1_G The  S-f"=    0.1565 Deviates from the B&C-Value     0.1550 Check
```

```
   0 ALERT level A = Most likely a serious problem - resolve or explain
   0 ALERT level B = A potentially serious problem, consider carefully
   8 ALERT level C = Check. Ensure it is not caused by an omission or oversight
  98 ALERT level G = General information/check it is not something unexpected

   6 ALERT type 1 CIF construction/syntax error, inconsistent or missing data
  10 ALERT type 2 Indicator that the structure model may be wrong or deficient
   6 ALERT type 3 Indicator that the structure quality may be low
  81 ALERT type 4 Improvement, methodology, query or suggestion
   3 ALERT type 5 Informative message, check
```

It is advisable to attempt to resolve as many as possible of the alerts in all categories. Often the minor alerts point to easily fixed oversights, errors and omissions in your CIF or refinement strategy, so attention to these fine details can be worthwhile. In order to resolve some of the more serious problems it may be necessary to carry out additional measurements or structure refinements. However, the purpose of your study may justify the reported deviations and the more serious of these should normally be commented upon in the discussion or experimental section of a paper or in the "special_details" fields of the CIF. checkCIF was carefully designed to identify outliers and unusual parameters, but every test has its limitations and alerts that are not important in a particular case may appear. Conversely, the absence of alerts does not guarantee there are no aspects of the results needing attention. It is up to the individual to critically assess their own results and, if necessary, seek expert advice.

Publication of your CIF in IUCr journals

A basic structural check has been run on your CIF. These basic checks will be run on all CIFs submitted for publication in IUCr journals (*Acta Crystallographica*, *Journal of Applied Crystallography*, *Journal of Synchrotron Radiation*); however, if you intend to submit to *Acta Crystallographica Section C* or *E* or *IUCrData*, you should make sure that full publication checks are run on the final version of your CIF prior to submission.

Publication of your CIF in other journals

Please refer to the *Notes for Authors* of the relevant journal for any special instructions relating to CIF submission.

PLATON version of 13/07/2021; check.def file version of 13/07/2021

Datablock ssm-1ab - ellipsoid plot

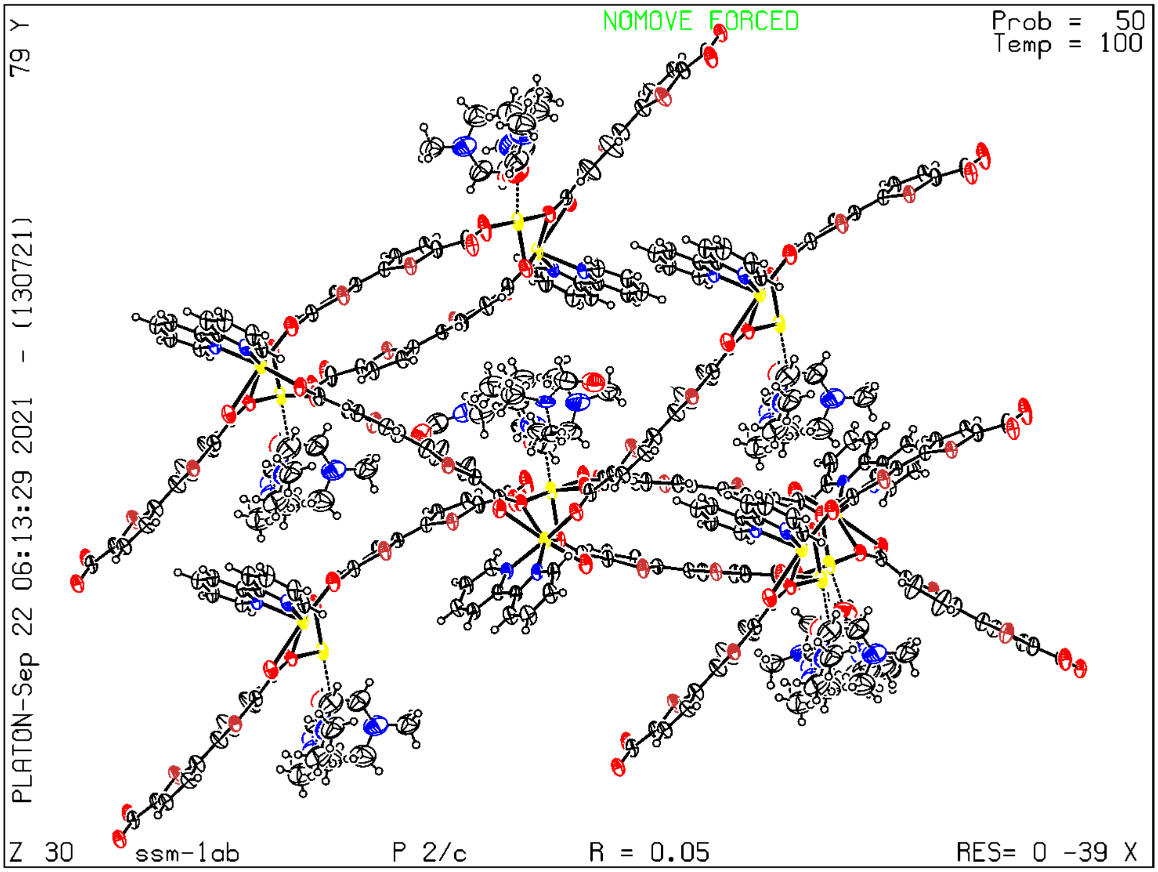

Supplement: Supplementary file 1 [file molecules-28-02139-s001.zip › 4-checkcif.pdf]
