# Supplementary material for: A Series of Metal–Organic Frameworks with 2,2′-Bipyridyl Derivatives: Synthesis vs. Structure Relationships, Adsorption, and Magnetic Studies
Source: Molecules. 2023 Feb 24;28(5):2139. doi: 10.3390/molecules28052139 (PMC10004071; doi:10.3390/molecules28052139)

checkCIF/PLATON report

Structure factors have been supplied for datablock(s) ssm7a

THIS REPORT IS FOR GUIDANCE ONLY. IF USED AS PART OF A REVIEW PROCEDURE FOR PUBLICATION, IT SHOULD NOT REPLACE THE EXPERTISE OF AN EXPERIENCED CRYSTALLOGRAPHIC REFEREE.

No syntax errors found. CIF dictionary Interpreting this report

Datablock: ssm7a

```
Bond precision:  C-C = 0.0039 A          Wavelength=0.71073

Cell:           a=14.9591(5)    b=17.0850(6)       c=17.8936(7)
                alpha=90        beta=104.678(4)    gamma=90
Temperature:    150 K
```

	Calculated	Reported
Volume	4423.9(3)	4423.9(3)
Space group	P 21/n	P 21/n
Hall group	-P 2yn	-P 2yn
Moiety formula	C35 H27 Mn2 N3 O9 S4, 1.5(C0.75 H1.75 N0.25 O0.25), C0.75 H1.75	?
Sum formula	C38 H34 Mn2 N4 O10 S4	C38 H34 Mn2 N4 O10 S4
Mr	944.81	944.81
Dx,g cm-3	1.419	1.419
Z	4	4
Mu (mm-1)	0.816	0.816
F000	1936.0	1936.0
F000’	1941.43	
h,k,lmax	20,23,24	20,23,23
Nref	11948	9914
Tmin,Tmax	0.802,0.885	0.987,1.000
Tmin’	0.802	

```
Correction method= # Reported T Limits: Tmin=0.987 Tmax=1.000
AbsCorr = MULTI-SCAN

Data completeness= 0.830          Theta(max)= 29.163

R(reflections)= 0.0409( 8392)     wR2(reflections)= 0.1245( 9914)

S = 1.047                   Npar= 623
```

The following ALERTS were generated. Each ALERT has the format
test-name_ALERT_alert-type_alert-level.
Click on the hyperlinks for more details of the test.

Alert level C

```
PLAT220_ALERT_2_C NonSolvent   Resd 1  C   Ueq(max)/Ueq(min) Range        3.9 Ratio
PLAT222_ALERT_3_C NonSolvent Resd 1  H   Uiso(max)/Uiso(min) Range        4.5 Ratio
PLAT906_ALERT_3_C Large K Value in the Analysis of Variance ......      2.533 Check
PLAT911_ALERT_3_C Missing FCF Refl Between Thmin & STh/L=    0.600          8 Report
```

Alert level G

```
PLAT002_ALERT_2_G Number of Distance or Angle Restraints on AtSite         15 Note
PLAT003_ALERT_2_G Number of Uiso or Uij Restrained non-H Atoms ...         15 Report
PLAT004_ALERT_5_G Polymeric Structure Found with Maximum Dimension          2 Info
PLAT176_ALERT_4_G The CIF-Embedded .res File Contains SADI Records          6 Report
PLAT178_ALERT_4_G The CIF-Embedded .res File Contains SIMU Records          1 Report
PLAT300_ALERT_4_G Atom Site Occupancy of O2D        Constrained at        0.5 Check
PLAT300_ALERT_4_G Atom Site Occupancy of N2D        Constrained at        0.5 Check
PLAT300_ALERT_4_G Atom Site Occupancy of C21D       Constrained at        0.5 Check
PLAT300_ALERT_4_G Atom Site Occupancy of C22D       Constrained at        0.5 Check
PLAT300_ALERT_4_G Atom Site Occupancy of C23D       Constrained at        0.5 Check
PLAT300_ALERT_4_G Atom Site Occupancy of H21D       Constrained at        0.5 Check
PLAT300_ALERT_4_G Atom Site Occupancy of H22A       Constrained at        0.5 Check
PLAT300_ALERT_4_G Atom Site Occupancy of H22B       Constrained at        0.5 Check
PLAT300_ALERT_4_G Atom Site Occupancy of H22C       Constrained at        0.5 Check
PLAT300_ALERT_4_G Atom Site Occupancy of H23A       Constrained at        0.5 Check
PLAT300_ALERT_4_G Atom Site Occupancy of H23B       Constrained at        0.5 Check
PLAT300_ALERT_4_G Atom Site Occupancy of H23C       Constrained at        0.5 Check
PLAT300_ALERT_4_G Atom Site Occupancy of O3D        Constrained at       0.25 Check
PLAT300_ALERT_4_G Atom Site Occupancy of N3D        Constrained at       0.25 Check
PLAT300_ALERT_4_G Atom Site Occupancy of C31D       Constrained at       0.25 Check
PLAT300_ALERT_4_G Atom Site Occupancy of C32D       Constrained at       0.25 Check
PLAT300_ALERT_4_G Atom Site Occupancy of C33D       Constrained at       0.25 Check
PLAT300_ALERT_4_G Atom Site Occupancy of H31D       Constrained at       0.25 Check
PLAT300_ALERT_4_G Atom Site Occupancy of H32A       Constrained at       0.25 Check
PLAT300_ALERT_4_G Atom Site Occupancy of H32B       Constrained at       0.25 Check
PLAT300_ALERT_4_G Atom Site Occupancy of H32C       Constrained at       0.25 Check
PLAT300_ALERT_4_G Atom Site Occupancy of H33A       Constrained at       0.25 Check
PLAT300_ALERT_4_G Atom Site Occupancy of H33B       Constrained at       0.25 Check
PLAT300_ALERT_4_G Atom Site Occupancy of H33C       Constrained at       0.25 Check
PLAT300_ALERT_4_G Atom Site Occupancy of O4D        Constrained at       0.25 Check
PLAT300_ALERT_4_G Atom Site Occupancy of N4D        Constrained at       0.25 Check
PLAT300_ALERT_4_G Atom Site Occupancy of C41D       Constrained at       0.25 Check
PLAT300_ALERT_4_G Atom Site Occupancy of C42D       Constrained at       0.25 Check
PLAT300_ALERT_4_G Atom Site Occupancy of C43D       Constrained at       0.25 Check
PLAT300_ALERT_4_G Atom Site Occupancy of H41D       Constrained at       0.25 Check
PLAT300_ALERT_4_G Atom Site Occupancy of H42A       Constrained at       0.25 Check
PLAT300_ALERT_4_G Atom Site Occupancy of H42B       Constrained at       0.25 Check
PLAT300_ALERT_4_G Atom Site Occupancy of H42C       Constrained at       0.25 Check
PLAT300_ALERT_4_G Atom Site Occupancy of H43A       Constrained at       0.25 Check
PLAT300_ALERT_4_G Atom Site Occupancy of H43B       Constrained at       0.25 Check
PLAT300_ALERT_4_G Atom Site Occupancy of H43C       Constrained at       0.25 Check
PLAT302_ALERT_4_G Anion/Solvent/Minor-Residue Disorder (Resd  2  )       100% Note
PLAT302_ALERT_4_G Anion/Solvent/Minor-Residue Disorder (Resd  3  )       100% Note
PLAT302_ALERT_4_G Anion/Solvent/Minor-Residue Disorder (Resd  4  )       100% Note
PLAT720_ALERT_4_G Number of Unusual/Non-Standard Labels ..........          6 Note
PLAT789_ALERT_4_G Atoms with Negative _atom_site_disorder_group  #         24 Check
PLAT794_ALERT_5_G Tentative Bond Valency for Mn1       (II)      .       2.05 Info
PLAT794_ALERT_5_G Tentative Bond Valency for Mn2       (II)      .       2.18 Info
```

```
PLAT860_ALERT_3_G Number of Least-Squares Restraints .............         384 Note
PLAT910_ALERT_3_G Missing # of FCF Reflection(s) Below Theta(Min).           2 Note
PLAT912_ALERT_4_G Missing # of FCF Reflections Above STh/L=  0.600        1994 Note
PLAT933_ALERT_2_G Number of OMIT Records in Embedded .res File ...           8 Note
PLAT941_ALERT_3_G Average HKL Measurement Multiplicity ...........         2.4 Low
PLAT961_ALERT_5_G Dataset Contains no Negative Intensities .......     Please Check
PLAT978_ALERT_2_G Number C-C Bonds with Positive Residual Density.           1 Info
```

```
   0 ALERT level A = Most likely a serious problem - resolve or explain
   0 ALERT level B = A potentially serious problem, consider carefully
   4 ALERT level C = Check. Ensure it is not caused by an omission or oversight
  55 ALERT level G = General information/check it is not something unexpected

   0 ALERT type 1 CIF construction/syntax error, inconsistent or missing data
   5 ALERT type 2 Indicator that the structure model may be wrong or deficient
   6 ALERT type 3 Indicator that the structure quality may be low
  44 ALERT type 4 Improvement, methodology, query or suggestion
   4 ALERT type 5 Informative message, check
```

It is advisable to attempt to resolve as many as possible of the alerts in all categories. Often the minor alerts point to easily fixed oversights, errors and omissions in your CIF or refinement strategy, so attention to these fine details can be worthwhile. In order to resolve some of the more serious problems it may be necessary to carry out additional measurements or structure refinements. However, the purpose of your study may justify the reported deviations and the more serious of these should normally be commented upon in the discussion or experimental section of a paper or in the "special_details" fields of the CIF. checkCIF was carefully designed to identify outliers and unusual parameters, but every test has its limitations and alerts that are not important in a particular case may appear. Conversely, the absence of alerts does not guarantee there are no aspects of the results needing attention. It is up to the individual to critically assess their own results and, if necessary, seek expert advice.

Publication of your CIF in IUCr journals

A basic structural check has been run on your CIF. These basic checks will be run on all CIFs submitted for publication in IUCr journals (*Acta Crystallographica*, *Journal of Applied Crystallography*, *Journal of Synchrotron Radiation*); however, if you intend to submit to *Acta Crystallographica Section C* or *E* or *IUCrData*, you should make sure that full publication checks are run on the final version of your CIF prior to submission.

Publication of your CIF in other journals

Please refer to the *Notes for Authors* of the relevant journal for any special instructions relating to CIF submission.

PLATON version of 22/03/2021; check.def file version of 19/03/2021

Datablock ssm7a - ellipsoid plot

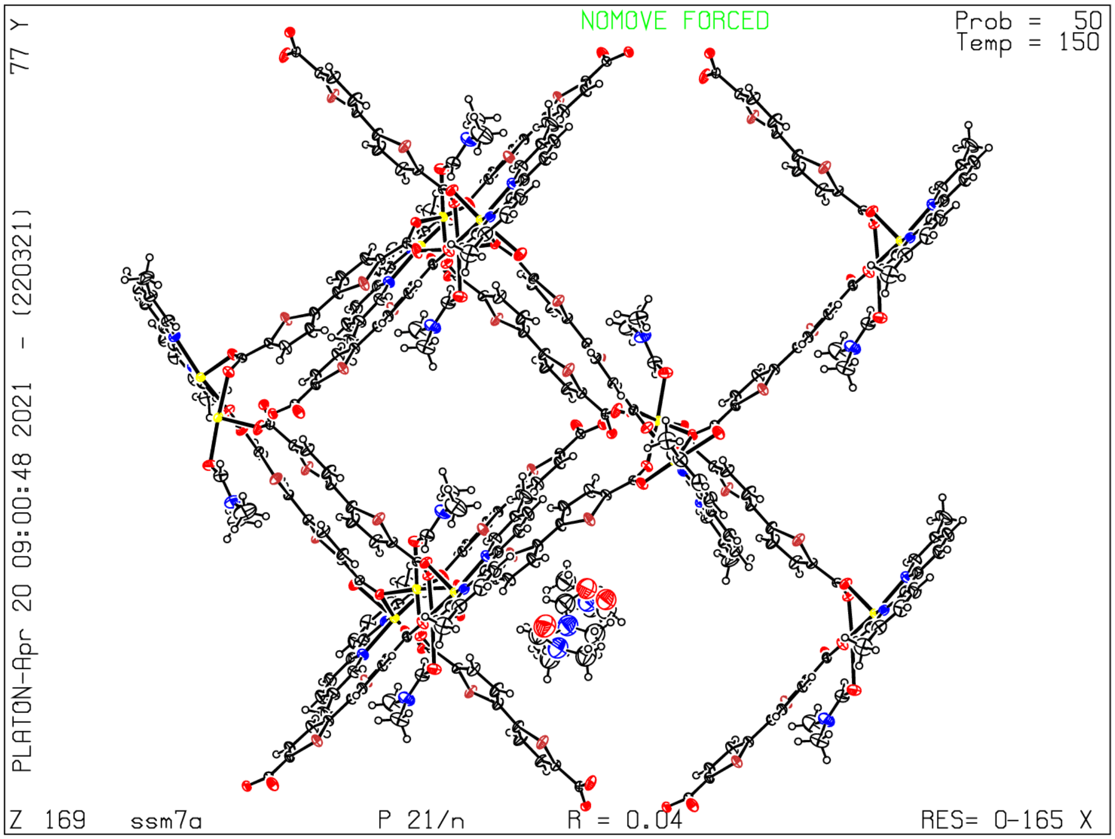

Supplement: Supplementary file 1 [file molecules-28-02139-s001.zip › 5-checkcif.pdf]
