# Supplementary material for: A Series of Metal–Organic Frameworks with 2,2′-Bipyridyl Derivatives: Synthesis vs. Structure Relationships, Adsorption, and Magnetic Studies
Source: Molecules. 2023 Feb 24;28(5):2139. doi: 10.3390/molecules28052139 (PMC10004071; doi:10.3390/molecules28052139)

checkCIF/PLATON report

Structure factors have been supplied for datablock(s) agm1

THIS REPORT IS FOR GUIDANCE ONLY. IF USED AS PART OF A REVIEW PROCEDURE FOR PUBLICATION, IT SHOULD NOT REPLACE THE EXPERTISE OF AN EXPERIENCED CRYSTALLOGRAPHIC REFEREE.

No syntax errors found. CIF dictionary Interpreting this report

Datablock: agm1

Bond precision: C-C = 0.0037 A Wavelength=0.74500

Cell:	a=10.880(5)	b=25.477(1)	c=12.919(4)
	alpha=90	beta=109.29(2)	gamma=90
Temperature:	100 K		

	Calculated	Reported
Volume	3380.0(19)	3379.9(19)
Space group	P 21/c	P 21/c
Hall group	-P 2ybc	-P 2ybc
Moiety formula	C50 H28 Mn3 N4 O12 S6, 2(C6 H6)	?
Sum formula	C62 H40 Mn3 N4 O12 S6	C62 H40 Mn3 N4 O12 S6
Mr	1390.16	1390.16
Dx,g cm-3	1.366	1.366
Z	2	2
Mu (mm-1)	0.899	0.899
F000	1414.0	1414.0
F000’	1418.29	
h,k,lmax	15,35,17	15,32,17
Nref	9423	8877
Tmin,Tmax	0.914,0.939	0.876,1.000
Tmin’	0.914	

Correction method= # Reported T Limits: Tmin=0.876 Tmax=1.000
AbsCorr = MULTI-SCAN

Data completeness= 0.942 Theta(max)= 31.102

R(reflections)= 0.0502(7395) wR2(reflections)= 0.1530(8877)

S = 1.061 Npar= 505

The following ALERTS were generated. Each ALERT has the format
test-name_ALERT_alert-type_alert-level.
Click on the hyperlinks for more details of the test.

Alert level B

PLAT029_ALERT_3_B _diffrn_measured_fraction_theta_full value Low . 0.957 Why?

Alert level C

PLAT250_ALERT_2_C Large U3/U1 Ratio for Average U(i,j) Tensor .... 2.2 Note
PLAT906_ALERT_3_C Large K Value in the Analysis of Variance ...... 2.659 Check
PLAT911_ALERT_3_C Missing FCF Refl Between Thmin & STh/L= 0.600 260 Report
PLAT913_ALERT_3_C Missing # of Very Strong Reflections in FCF .... 8 Note
PLAT918_ALERT_3_C Reflection(s) with I(obs) much Smaller I(calc) . 2 Check

Alert level G

ABSMU01_ALERT_1_G Calculation of _exptl_absorpt_correction_mu
not performed for this radiation type.
PLAT002_ALERT_2_G Number of Distance or Angle Restraints on AtSite 18 Note
PLAT003_ALERT_2_G Number of Uiso or Uij Restrained non-H Atoms ... 18 Report
PLAT004_ALERT_5_G Polymeric Structure Found with Maximum Dimension 3 Info
PLAT092_ALERT_4_G Check: Wavelength Given is not Cu,Ga,Mo,Ag,In Ka 0.74500 Ang.
PLAT172_ALERT_4_G The CIF-Embedded .res File Contains DFIX Records 1 Report
PLAT174_ALERT_4_G The CIF-Embedded .res File Contains FLAT Records 3 Report
PLAT176_ALERT_4_G The CIF-Embedded .res File Contains SADI Records 3 Report
PLAT178_ALERT_4_G The CIF-Embedded .res File Contains SIMU Records 1 Report
PLAT302_ALERT_4_G Anion/Solvent/Minor-Residue Disorder (Resd 2) 100% Note
PLAT302_ALERT_4_G Anion/Solvent/Minor-Residue Disorder (Resd 3) 100% Note
PLAT302_ALERT_4_G Anion/Solvent/Minor-Residue Disorder (Resd 4) 100% Note
PLAT794_ALERT_5_G Tentative Bond Valency for Mn2 (II) . 2.14 Info
PLAT860_ALERT_3_G Number of Least-Squares Restraints .... 721 Note
PLAT910_ALERT_3_G Missing # of FCF Reflection(s) Below Theta(Min). 2 Note
PLAT912_ALERT_4_G Missing # of FCF Reflections Above STh/L= 0.600 195 Note
PLAT933_ALERT_2_G Number of OMIT Records in Embedded .res File ... 4 Note
PLAT941_ALERT_3_G Average HKL Measurement Multiplicity .... 3.8 Low
PLAT951_ALERT_5_G Calculated (ThMax) and CIF-Reported Kmax Differ 3 Units
PLAT957_ALERT_1_G Calculated (ThMax) and Actual (FCF) Kmax Differ 3 Units
PLAT961_ALERT_5_G Dataset Contains no Negative Intensities .... Please Check
PLAT978_ALERT_2_G Number C-C Bonds with Positive Residual Density. 9 Info
PLAT984_ALERT_1_G The Mn-f'= 0.3487 Deviates from the B&C-Value 0.3468 Check
PLAT984_ALERT_1_G The S-f'= 0.1364 Deviates from the B&C-Value 0.1346 Check
PLAT985_ALERT_1_G The Mn-f"= 0.8030 Deviates from the B&C-Value 0.7995 Check
PLAT985_ALERT_1_G The S-f"= 0.1381 Deviates from the B&C-Value 0.1368 Check

0 **ALERT level A** = Most likely a serious problem - resolve or explain
1 **ALERT level B** = A potentially serious problem, consider carefully
5 **ALERT level C** = Check. Ensure it is not caused by an omission or oversight
26 **ALERT level G** = General information/check it is not something unexpected

6 ALERT type 1 CIF construction/syntax error, inconsistent or missing data

```
 5 ALERT type 2 Indicator that the structure model may be wrong or deficient
 8 ALERT type 3 Indicator that the structure quality may be low
 9 ALERT type 4 Improvement, methodology, query or suggestion
 4 ALERT type 5 Informative message, check
```

It is advisable to attempt to resolve as many as possible of the alerts in all categories. Often the minor alerts point to easily fixed oversights, errors and omissions in your CIF or refinement strategy, so attention to these fine details can be worthwhile. In order to resolve some of the more serious problems it may be necessary to carry out additional measurements or structure refinements. However, the purpose of your study may justify the reported deviations and the more serious of these should normally be commented upon in the discussion or experimental section of a paper or in the "special_details" fields of the CIF. checkCIF was carefully designed to identify outliers and unusual parameters, but every test has its limitations and alerts that are not important in a particular case may appear. Conversely, the absence of alerts does not guarantee there are no aspects of the results needing attention. It is up to the individual to critically assess their own results and, if necessary, seek expert advice.

Publication of your CIF in IUCr journals

A basic structural check has been run on your CIF. These basic checks will be run on all CIFs submitted for publication in IUCr journals (*Acta Crystallographica*, *Journal of Applied Crystallography*, *Journal of Synchrotron Radiation*); however, if you intend to submit to *Acta Crystallographica Section C* or *E* or *IUCrData*, you should make sure that full publication checks are run on the final version of your CIF prior to submission.

Publication of your CIF in other journals

Please refer to the *Notes for Authors* of the relevant journal for any special instructions relating to CIF submission.

PLATON version of 13/07/2021; check.def file version of 13/07/2021

Datablock agm1 - ellipsoid plot

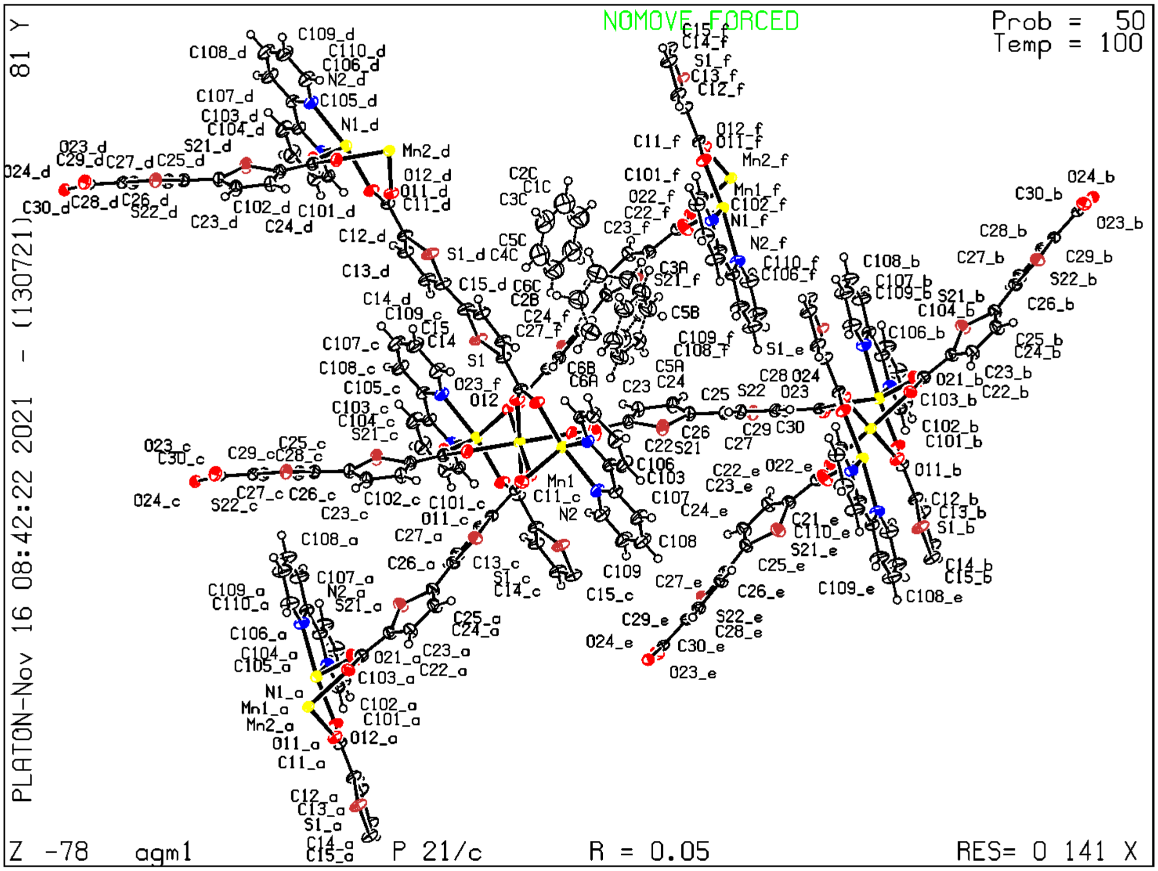

Supplement: Supplementary file 1 [file molecules-28-02139-s001.zip › 6-checkcif.pdf]
