# Supplementary material for: A Series of Metal–Organic Frameworks with 2,2′-Bipyridyl Derivatives: Synthesis vs. Structure Relationships, Adsorption, and Magnetic Studies
Source: Molecules. 2023 Feb 24;28(5):2139. doi: 10.3390/molecules28052139 (PMC10004071; doi:10.3390/molecules28052139)
Supplement: Supplementary file 1 [file molecules-28-02139-s001.zip › Supplementary Materials tables and figures.pdf]

A Series of Metal-Organic Frameworks with 2,2′-Bipyridyl Derivatives: Synthesis vs. Structure Relationships, Adsorption and Magnetic Studies

Vadim A. Dubskikh [1], Aleksei A. Kolosov [1,2], Anna A. Lysova [1,*], Denis G. Samsonenko [1], Alexander N. Lavrov [1], Konstantin A. Kovalenko [1], Danil N. Dybtsev [1,*] and Vladimir P. Fedin [1]

[1] Nikolaev Institute of Inorganic Chemistry, Siberian Branch of the Russian Academy of Sciences, Novosibirsk 630090, Russia
[2] Department of Natural Sciences, Novosibirsk State University, Novosibirsk 630090, Russia
* Correspondence: lysova@niic.nsc.ru (A.A.L.); dan@niic.nsc.ru (D.N.D.)

Materials and Methods

Instruments and methods

Infrared spectra of solid samples as KBr pellets were recorded using an IR-Fourier spectrometer Scimitar FTS 2000 (4000-400 cm^{-1}). The **elemental analyses** were obtained on an analyzer «Vario Micro-Cube». **The thermogravimetric analyses** were carried out in He atmosphere on NETZSCH TG 209 F1 thermoanalyzer with the heating rate of 10 deg/min. The **powder X-ray diffraction** data were obtained on a «Shimadzu XRD 7000S» powder diffractometer (Cu-Kα irradiation, λ = 1.54178 Å). **Surface area and porous structure** were analyzed using the nitrogen adsorption technique on a Quantochrome's Autosorb iQ gas sorption analyzer at 77 K. Prior to the isotherm measurements the as-synthesized crystals of the compound **1** were placed in CH_2Cl_2 for five days. Each day the crystals were separated from the solvent by decantation, and a new portion of CH_2Cl_2 was added. After 5 days the crystals were separated from the solvent, placed into the gas-measurement cell and, finally, activated under a dynamic vacuum at 180 °C for 6 h. The nitrogen adsorption−desorption isotherms were measured within the range of relative pressures from 10^{-6} to 0.995. The specific surface area was calculated from the data obtained using the conventional BET and DFT models. **Gas adorption experiments at 273 and 298 K** were carried out volumetrically on Quantochrome's Autosorb iQ equiped with thermostat TERMEX Cryo-VT-12 to adjust temperature with 0.1 K accuracy. Adsoprtion−desorption isotherms were measured within the range of pressures of 1 to 800 torr. The database of the National Institute of Standards and Technology availavble at http://webbook.nist.gov/chemistry/fluid/ was used as a source of $p-V-T$ relations at experimental pressures and temperatures. **Vapor adorption experiments at 298 K** were carried out volumetrically on Quantochrome's Autosorb iQ equiped with thermostat TERMEX Cryo-VT-12 to adjust temperature with 0.1 K accuracy. **Magnetization** measurements were carried out using a Quantum Design MPMS-XL SQUID magnetometer in the temperature range 1.77–330 K at magnetic fields up to 10 kOe. Temperature dependences of the magnetization, *M(T)*, were measured on heating the sample after it was cooled either in zero magnetic field or in a given magnetic field as well as upon cooling the sample. In order to determine the paramagnetic component of the molar magnetic susceptibility, $\chi_P(T)$, the temperature-independent diamagnetic contribution, χ_d, and a possible magnetization of ferromagnetic micro-impurities, $\chi_{FM}(T)$, were evaluated and subtracted from the measured values of the total molar susceptibility $\chi=M/H$. While χ_d was calculated using the Pascal's additive scheme, $\chi_{FM}(T)$, if any, was determined from the measured isothermal *M(H)* dependencies and the *M(T)* data taken at different magnetic fields. To determine the effective magnetic moment of manganese Mn(II) ions, μ_{eff}, the paramagnetic susceptibility $\chi_P(T)$ was analysed using the Curie-Weiss dependence $\chi_P(T) = N_a \cdot \mu_{eff}^2/3 \cdot k_B \cdot (T - \theta)$, where N_A and k_B are the Avogadro and Boltzmann numbers, respectively.

Single-Crystal X-ray Diffraction

Diffraction data for single crystals of compounds **1–3** and **5** were obtained on an automated Agilent Xcalibur diffractometer equipped with an area CCD AtlasS2 detector (MoKα, graphite monochromator, ω-scans with a step of 0.25º). Integration, absorption correction, and determination of unit cell parameters were performed using the CrysAlisPro program package [57]. Diffraction data for single-crystals **4** and **1⊇2C$_6$H$_6$** were collected on the 'Belok/XSA' beamline (φ-scans with a step of 1.0°) of the National Research Center 'Kurchatov Institute' (Moscow, Russian Federation) using a Rayonix SX165 CCD detector [58, 59]. The data were indexed, integrated and scaled, and an absorption correction was applied using the XDS program package [60]. The structures were solved by the dual space algorithm (SHELXT [61]) and refined

by the full-matrix least squares technique (SHELXL [62]) in the anisotropic approximation (except hydrogen atoms). Positions of hydrogen atoms of organic ligands were calculated geometrically and refined in the riding model. The crystallographic data and details of the structure refinements are summarized in Table S1 and S2. CCDC 2227366–2227371 contains the supplementary crystallographic data for this paper. These data can be obtained free of charge from The Cambridge Crystallographic Data Center at https://www.ccdc.cam.ac.uk/structures/.

Synthesis of coordination polymers

The ligand synthesis was carried out according to the known methodology [63]. The starting substances were used as commercially available reagents without further purification.

Synthesis of $[Mn_3(btdc)_3(bpy)_2]\cdot 4DMF$ (**1**). Manganese(II) perchlorate hexahydrate (36.2 mg, 0.1 mmol), 2,2′-bithiophen-5,5′-dicarboxylic acid (H_2btdc, 25.4 mg, 0.1 mmol), 2,2′-bipyridyl (bpy, 31.2 mg, 0.2 mmol) and 5 ml of N,N-dimethylformamide (DMF) were placed in a glass vial with a screw cap. The reaction mixture was sonicated for 20 minutes, and then heated at 120 °C for 2 days. The resulting crystals were washed with DMF (3×5 ml) and dried in air. Yield 0.0277 g (55 %). Crystal Data for $C_{62}H_{56}N_8O_{16}S_6Mn_3$ (M = 1526.33 g/mol): monoclinic, space group $P\ 2_1/c$ (no. 14), a = 10.9298(4) Å, b = 25.5452(11) Å, c = 13.0284(5) Å, β = 111.044(4)°, V = 3395.0(2) Å^3, Z = 2, T = 150 K, μ(0.71073 Å) = 0.806 mm^{-1}, D_{calc} = 1.493 g·cm^{-3}, 16983 reflections measured (3.72° ≤ 2θ ≤ 51.36°), 6446 unique (R_{int} = 0.0233) which were used in all calculations. The final R_1 was 0.0395 ($I > 2\sigma(I)$), wR_2 was 0.1160 (all data) and GoF = 1.041. Anal. Calc. for $[Mn_3(btdc)_3(bpy)_2]\cdot 4DMF$ = $C_{62}H_{56}N_8O_{16}S_6Mn_3$(%): C 48.8, H 3.7, N 7.3, S 12.6 %. Found: C 49.0, H 3.6, N 7.0, S 12.9 %. IR data (cm^{-1}): 422(m), 650(m), 737(m), 770(s), 794(m), 818(m), 885(w), 1018(w), 1060(w), 1117(w), 1170(w), 1377(s), 1436(s), 1472(m), 1515(s), 1594(s), 1665(m), 2851(w), 2929(w), 3091(w), 3352(w, broad).

Synthesis of $[Mn_3(btdc)_3(5,5'\text{-}dmbpy)_2]\cdot 5DMF$ (**2**). Manganese(II) perchlorate hexahydrate (36.6 mg, 0.1 mmol), 2,2′-bithiophen-5,5′-dicarboxylic acid (H_2btdc, 25.4 mg, 0.1 mmol), 5,5′-dimethyl-2,2′-bipyridyl (5,5′-dmbpy, 36.8 mg, 0.2 mmol) and 5 ml of N,N-dimethylformamide (DMF) were placed in a glass vial with a screw cap. The reaction mixture was sonicated for 20 minutes, and then heated at 130 °C for 2 days. The resulting crystals were washed with DMF (3×5 ml) and dried in air. Yield 0.0337 g (61 %). Crystal Data for $C_{69}H_{71}N_9O_{17}S_6Mn_3$ (M = 1655.52 g/mol): monoclinic, space group $P\ 2_1/n$ (no. 14), a = 16.4304(5) Å, b = 25.1354(5) Å, c = 18.4058(5) Å, β = 94.705(3)°, V = 7575.7(4) Å^3, Z = 4, T = 150 K, μ(0.71073 Å) = 0.730 mm^{-1}, D_{calc} = 1.452 g·cm^{-3}, 40975 reflections measured (4.08° ≤ 2θ ≤ 58.12°), 16855 unique (R_{int} = 0.0194) which were used in all calculations. The final R_1 was 0.0323 ($I > 2\sigma(I)$), wR_2 was 0.0840 (all data) and GoF = 1.046. Anal. Calc. for $[Mn_3(btdc)_3(4,4'\text{-}dmbpy)_2]\cdot 5DMF$ = $C_{66}H_{62}N_8O_{16}S_6Mn_3$(%): C 50.1, H 4.1, N 7.1, S 12.2 %. Found: C 49.8, H 3.9, N 6.8, S 12.0 %. IR data (cm^{-1}): 443(w), 517(w), 538(w), 650(w), 764(m), 805(m), 831(m), 882(w), 890(w), 1010(m), 1042(m), 1200(w), 1237(m), 1289(m), 1389(s), 1439(s), 1519(s), 1564(s), 1612(s), 2917(w), 3072(w), 3415(w, broad).

Synthesis of $[Mn(btdc)(4,4'\text{-}dmbpy)]$ (**3**). Manganese(II) perchlorate e hexahydrate (36.2 mg, 0.1 mmol), 2,2′-bithiophen-5,5′-dicarboxylic acid (H_2btdc, 25.4 mg, 0.1 mmol), 4,4′-dimethyl-2,2′-bipyridyl (4,4′-dmbpy, 18.4 mg, 0.1 mmol) and 5 ml of N,N-dimethylformamide (DMF) were placed in a glass vial with a screw cap. The reaction mixture was sonicated for 20 minutes, and then heated at 120 °C for 2 days. The resulting crystals were washed with DMF (3×5 ml) and dried in air. Yield 0.0225 g (46 %). Crystal Data for $C_{22}H_{16}N_2O_4S_2Mn$ (M = 491.43 g/mol): triclinic, space group P-1 (no. 2), a = 10.5007(5) Å, b = 10.5738(6) Å, c = 11.0290(5) Å, α = 102.277(4)°, β = 91.709(4)°, γ = 117.108(5)°, V = 1053.76(10) Å^3, Z = 2, T = 140 K, μ(0.71073 Å) = 0.857 mm^{-1}, D_{calc} = 1.549 g·cm^{-3}, 7812 reflections measured (4.40° ≤ 2θ ≤ 58.08°), 4607 unique (R_{int} = 0.0179) which were used in all calculations. The final R_1 was 0.0384 ($I > 2\sigma(I)$), wR_2 was 0.0936 (all data) and GoF = 1.046. Anal. Calc. for $[Mn(btdc)(5,5'\text{-}dmbpy)]$ = $C_{22}H_{16}N_2O_4S_2Mn$(%): C 53.8, H 3.3, N 5.7, S 13.0 %. Found: C 53.8, H 3.4, N 5.8, S 13.2 %. IR data (cm^{-1}): 428(m), 535(w), 654(w), 735(w), 773(s), 797(m), 812(m), 935(w), 1042 (m), 1087(w), 1114(w), 1162(w), 1245(m), 1330(s), 1378(s), 1426(s), 1477(m), 1522(s), 1595(s), 1679(s), 2850(w), 2924(w), 3074(w), 3432(w, broad).

Synthesis of $[Mn_2(btdc)_2(bpy)(dmf)]\cdot 0.5DMF$ (**4**). Manganese(II) perchlorate hexahydrate (36.2 mg, 0.1 mmol), 2,2′-bithiophen-5,5′-dicarboxylic acid (H_2btdc, 25.4 mg, 0.1 mmol), 2,2′-bipyridyl (bpy, 7.8 mg, 0.05 mmol) and 5 ml of N,N-dimethylformamide (DMF) were placed in a glass vial with a screw cap. The reaction mixture was sonicated for 20 minutes, and then heated at 110 °C for 2 days. Compound **4** was polluted by crystals of **1**, and we failed to isolate it as a pure phase. Crystal Data for $C_{34.5}H_{26.5}N_{3.5}O_{9.5}S_4Mn_2$ (M = 880.21 g/mol): monoclinic, space group $P\ 2/c$ (no. 13), a = 16.206(3) Å, b = 11.501(4) Å, c = 22.561(3) Å, β = 107.876(6)°, V = 4002.0(17) Å^3, Z = 4, T = 100 K, μ(0.79313 Å) = 1.203 mm^{-1}, D_{calc} = 1.461 g·cm^{-3}, 65746

reflections measured (1.94° ≤ 2θ ≤ 57.08°), 7148 unique (R_{int} = 0.0376) which were used in all calculations. The final R_1 was 0.0516 ($I > 2\sigma(I)$), wR_2 was 0.1533 (all data) and GoF = 1.028.

Synthesis of [Mn_2(btdc)$_2$(5,5′-dmbpy)(dmf)]·DMF (**5**). Manganese(II) perchlorate hexahydrate (36.2 mg, 0.1 mmol), 2,2′-bithiophen-5,5′-dicarboxylic acid (H_2btdc, 25.4 mg, 0.1 mmol), 5,5′-dimethyl-2,2′-bipyridyl (5,5′-dmbpy, 9.2 mg, 0.05 mmol) and 5 ml of N,N-dimethylformamide (DMF) were placed in a glass vial with a screw cap. The reaction mixture was sonicated for 20 minutes, and then heated at 120 °C for 2 days. Compound **5** was polluted by crystals of **2**, and we failed to isolate it as a pure phase. Crystal Data for $C_{38}H_{34}N_4O_{10}S_4Mn_2$ (M = 944.81 g/mol): monoclinic, space group $P\,2_1/n$ (no. 14), a = 14.9591(5) Å, b = 17.0850(6) Å, c = 17.8936(7) Å, β = 104678(4)°, V = 4423.9(3) Å^3, Z = 4, T = 150 K, μ(0.71073 Å) = 0.816 mm^{-1}, D_{calc} = 1.419 g·cm^{-3}, 23328 reflections measured (3.68° ≤ 2θ ≤ 58.32°), 9914 unique (R_{int} = 0.0184) which were used in all calculations. The final R_1 was 0.0409 ($I > 2\sigma(I)$), wR_2 was 0.124 (all data) and GoF = 1.047.

Synthesis of [Mn_3(btdc)$_3$(bpy)$_2$]·$2C_6H_6$ (**1≃2C₆H₆**). As synthesized compound **1** was places in a closed vial containing 5 ml of benzene for 5 days. Then the crystals were characterized by the single crystal X-ray diffraction. Crystal Data for $C_{62}H_{40}N_4O_{12}S_6Mn_3$ (M = 1390.16 g/mol): monoclinic, space group $P\,2_1/c$ (no. 14), a = 10.880(5) Å, b = 25.477(10) Å, c = 12.919(4) Å, β = 109.29(2)°, V = 3379.9(19) Å^3, Z = 2, T = 100 K, μ(0.74500 Å) = 0.899 mm^{-1}, D_{calc} = 1.366 g·cm^{-3}, 33656 reflections measured (4.16° ≤ 2θ ≤ 62.20°), 8877 unique (R_{int} = 0.0303) which were used in all calculations. The final R_1 was 0.0502 ($I > 2\sigma(I)$), wR_2 was 0.1530 (all data) and GoF = 1.061.

Table S1. Crystal data and structure refinement for **1–3**.

Parameter	**1**	**2**	**3**
Empirical formula	$C_{62}H_{56}N_8O_{16}S_6Mn_3$	$C_{69}H_{71}N_9O_{17}S_6Mn_3$	$C_{22}H_{16}N_2O_4S_2Mn$
M, g/mol	1526.33	1655.52	491.43
T, K	150	150	140
λ, Å	0.71073	0.71073	0.71073
Crystal system	*Monoclinic*	*Monoclinic*	*Triclinic*
Space group	$P2_1/c$	$P2_1/n$	$P-1$
a, Å	10.9298(4)	16.4304(5)	10.5007(5)
b, Å	25.5452(11)	25.1354(9)	10.5738(6)
c, Å	13.0284(5)	18.4058(5)	11.0290(5)
α, deg.	90	90	102.277(4)
β, deg.	111.044(4)	94.705(3)	91.709(4)
γ, deg.	90	90	117.108(5)
V, Å^3	3395.0(2)	7575.7(4)	1053.76(10)
Z	2	4	2
D(calc.), g/cm^3	1.493	1.452	1.549
μ, mm^{-1}	0.806	0.730	0.857
F(000)	1566	3420	502
Crystal size, mm	0.45 × 0.21 × 0.05	0.68 × 0.21 × 0.10	0.15 × 0.14 × 0.07
θ range for data collection, deg.	1.86–25.68	2.04–29.06	2.20–29.04
Index range	$-12 \le h \le 13$, $-31 \le k \le 31$, $-15 \le l \le 12$	$-21 \le h \le 20$, $-33 \le k \le 31$, $-18 \le l \le 24$	$-13 \le h \le 12$, $-14 \le k \le 12$, $-11 \le l \le 13$
Reflections collected / independent	16986 / 6446	40975 / 16855	7812 / 4607
R_{int}	0.0233	0.0194	0.0179
Reflections with $I > 2\sigma(I)$	5490	13294	3827
Goodness-of-fit on F^2	1.041	1.046	1.046
Final R indices [$I > 2\sigma(I)$]	$R_1 = 0.0395$, $wR_2 = 0.1109$	$R_1 = 0.0323$, $wR_2 = 0.0787$	$R_1 = 0.0384$, $wR_2 = 0.0884$
R indices (all data)	$R_1 = 0.0482$, $wR_2 = 0.1160$	$R_1 = 0.0467$, $wR_2 = 0.0840$	$R_1 = 0.0506$, $wR_2 = 0.0936$
Largest diff. peak / hole, e/Å^3	0.591 / −0.560	0.348 / −0.274	1.141 / −0.349

Table S2. Crystal data and structure refinement for **4**, **5** and **1≅2C$_6$H$_6$**.

Parameter	**4**	**5**	**1≅2C$_6$H$_6$**
Empirical formula	$C_{34.5}H_{26.5}N_{3.5}O_{9.5}S_4Mn_2$	$C_{38}H_{34}N_4O_{10}S_4Mn_2$	$C_{62}H_{40}N_4O_{12}S_6Mn_3$
M, g/mol	880.21	944.81	1390.16
T, K	100	150	100
λ, Å	0.79313	0.71073	0.74500
Crystal system	*Monoclinic*	*Monoclinic*	*Monoclinic*
Space group	$P2/c$	$P2_1/n$	$P2_1/c$
a, Å	16.206(3)	14.9591(5)	10.880(5)
b, Å	11.501(4)	17.0850(6)	25.477(10)
c, Å	22.561(3)	17.8936(7)	12.919(4)
α, deg.	90	90	90
β, deg.	107.876(6)	104.678(4)	109.29(2)
γ, deg.	90	90	90
V, Å^3	4002.0(17)	4423.9(3)	3379.9(19)
Z	4	4	2
D(calc.), g/cm^3	1.461	1.419	1.366
μ, mm^{-1}	1.203	0.816	0.899
F(000)	1792	1936	1414
Crystal size, mm	0.10 × 0.06 × 0.05	0.27 × 0.23 × 0.15	0.10 × 0.10 × 0.07
θ range for data collection, deg.	1.47–28.54	1.84–29.16	2.08–31.10
Index range	$-18 \le h \le 18$, $-13 \le k \le 13$, $-27 \le l \le 27$	$-20 \le h \le 16$, $-22 \le k \le 23$, $-14 \le l \le 23$	$-15 \le h \le 15$, $-32 \le k \le 30$, $-17 \le l \le 16$
Reflections collected / independent	65746 / 7148	23328 / 9914	33656 / 8877
R_{int}	0.0376	0.0184	0.0303
Reflections with $I > 2\sigma(I)$	6346	8392	7395
Goodness-of-fit on F^2	1.028	1.047	1.061
Final *R* indices [$I > 2\sigma(I)$]	$R_1 = 0.0516$, $wR_2 = 0.1493$	$R_1 = 0.0409$, $wR_2 = 0.1194$	$R_1 = 0.0502$, $wR_2 = 0.1459$
R indices (all data)	$R_1 = 0.0568$, $wR_2 = 0.1533$	$R_1 = 0.0506$, $wR_2 = 0.1245$	$R_1 = 0.0606$, $wR_2 = 0.1530$
Largest diff. peak / hole, *e*/Å^3	1.393 / −0.838	1.204 / −0.980	1.075 / −1.103

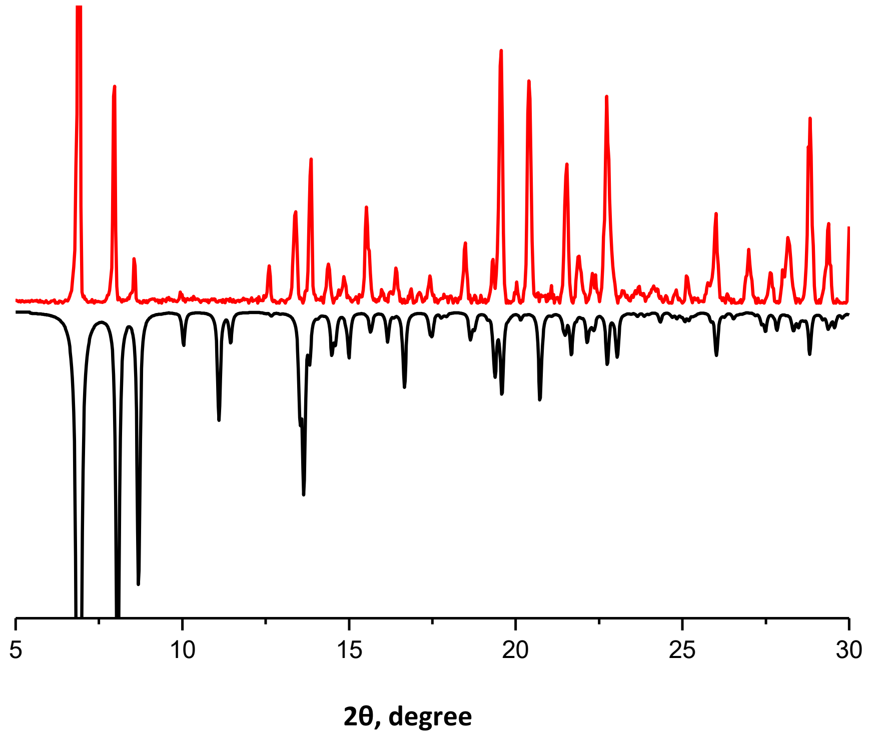

Figure S1. PXRD patterns of **1** (experimental – red, calculated from the single crystal X-ray diffraction data – black).

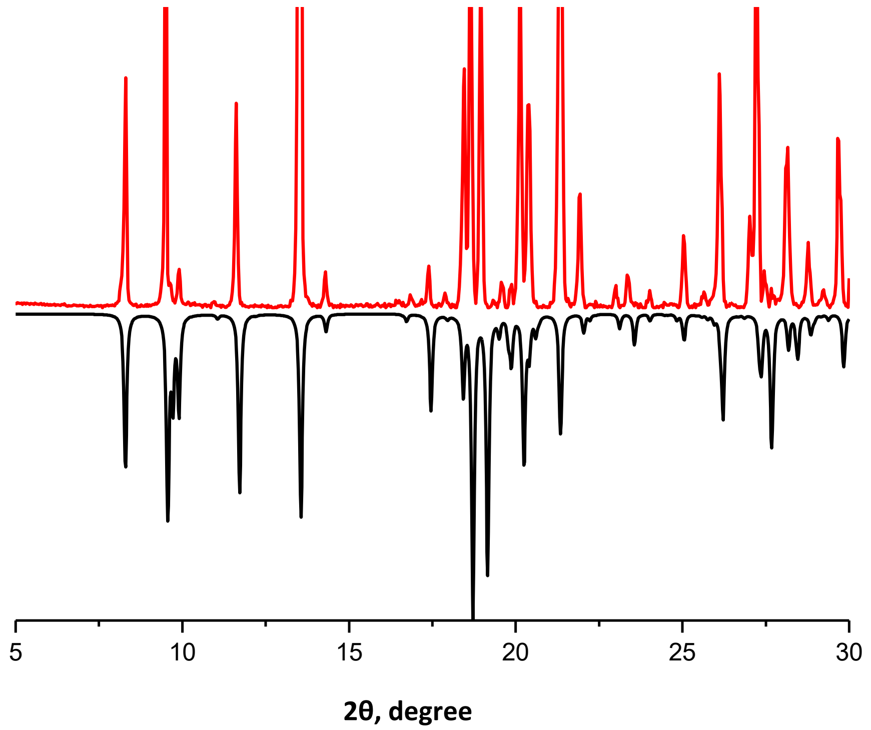

Figure S2. PXRD patterns of **2** (experimental – red, calculated from the single crystal X-ray diffraction data – black).

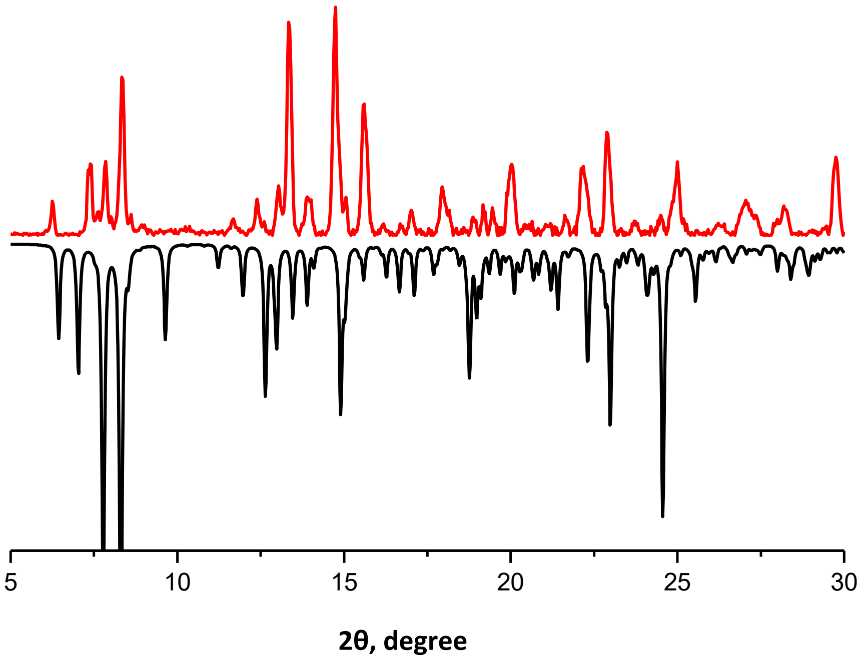

Figure S3. PXRD patterns of **3** (experimental – red, calculated from the single crystal X-ray diffraction data – black).

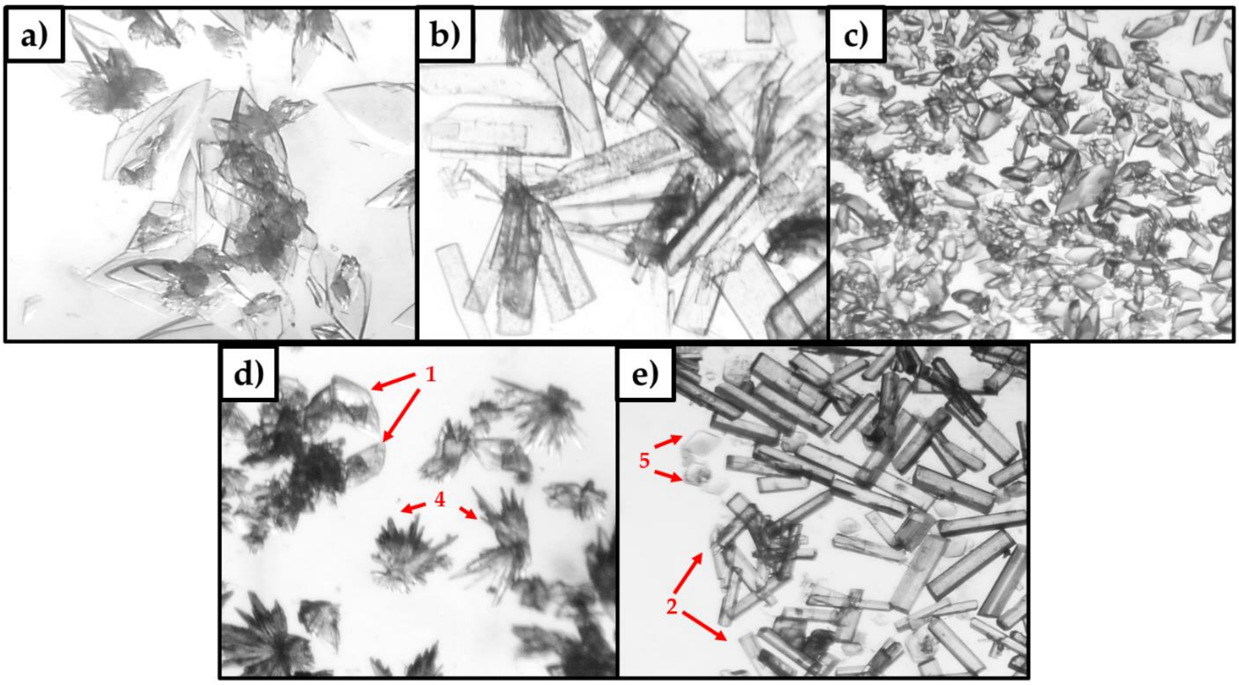

Figure S4. Optical images of the crystals: a) pure phase **1**; b) pure phase **2**; c) pure phase **3**; d) mixture of compounds **1** and **4**; e) mixture of compounds **2** and **5**.

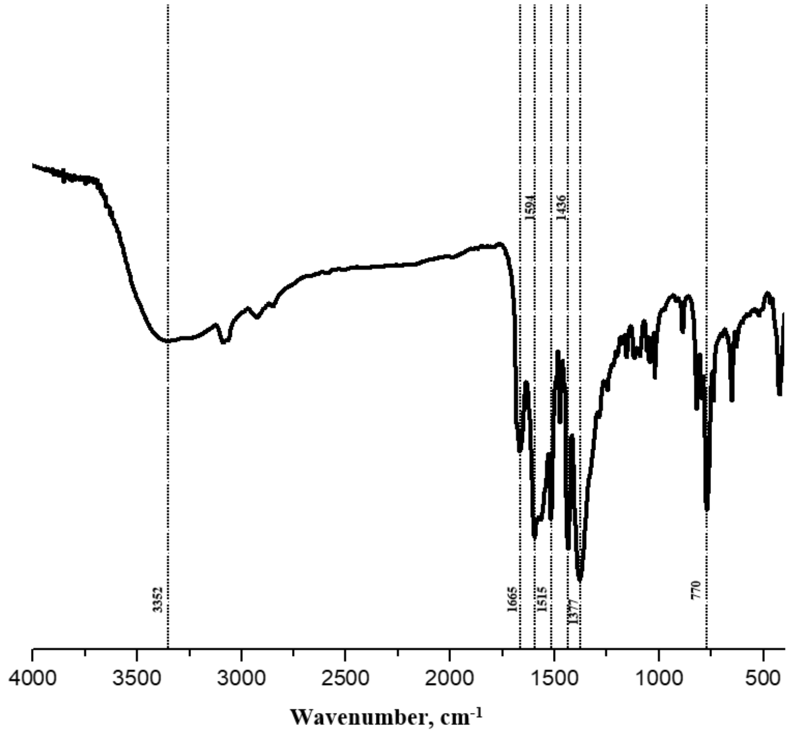

Figure S5. IR spectrum of **1**.

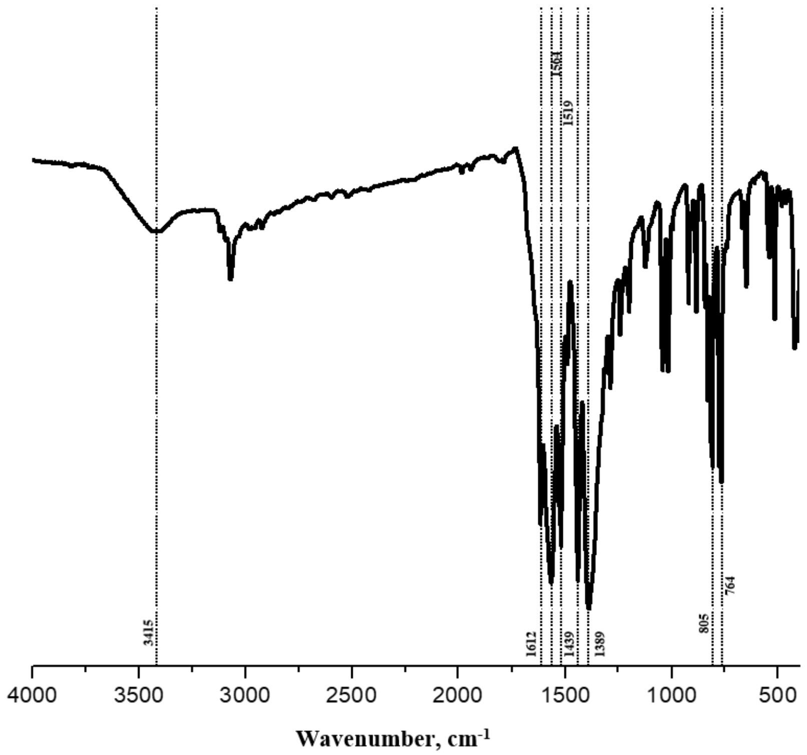

Figure S6. IR spectrum of **2**.

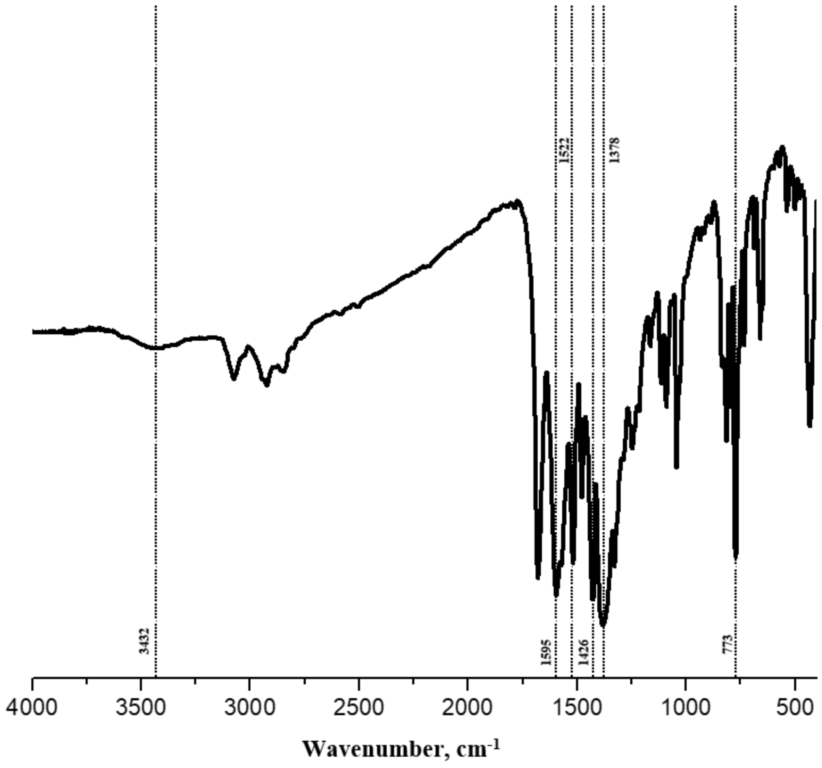

Figure S7. IR spectrum of **3**.

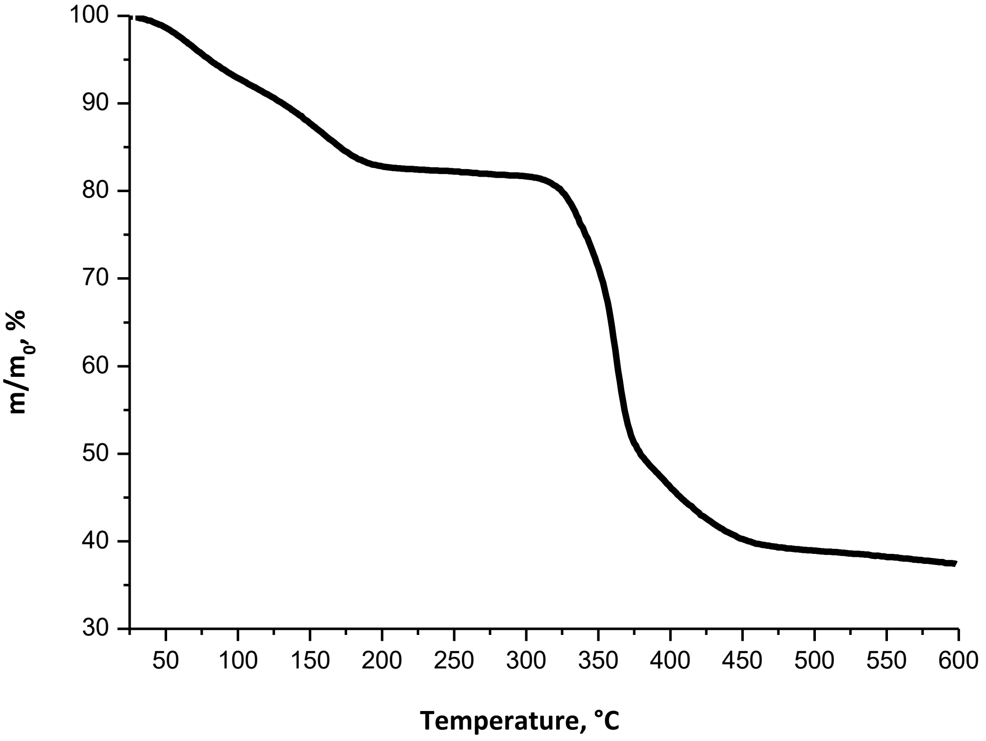

Figure S8. TG curve of **1**.

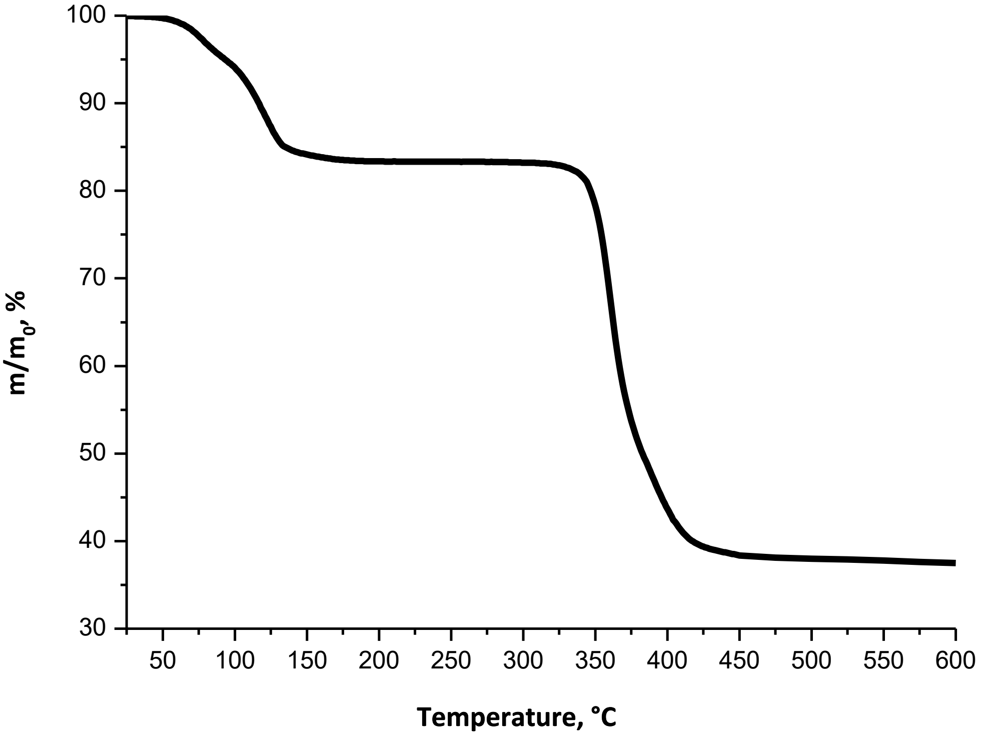

Figure S9. TG curve of **2**.

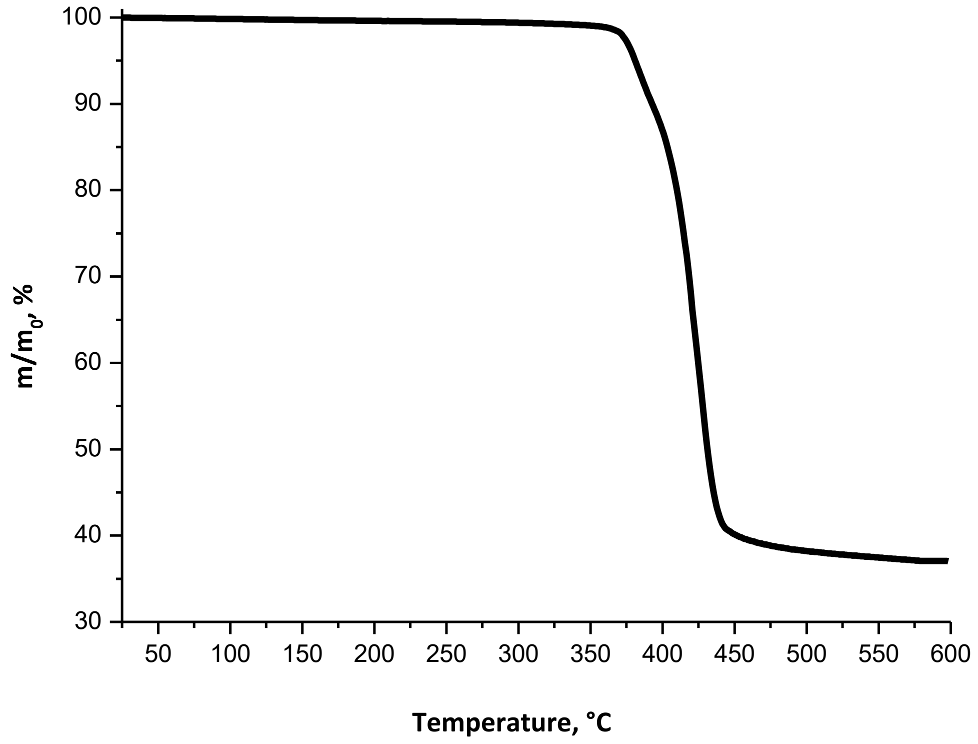

Figure S10. TG curve of **3**.

Table S3. The textural parameters of the porous structure of **1**.

**Specific surface area / $m^2 \cdot g^{-1}$**			**V_{pore} / $cm^3 \cdot g^{-1}$**		**$V_{ads}(N_2)^a$/ $cm^3(STP) \cdot g^{-1}$**
**Langmuir**	**BET**	**DFT**	**Total[a]**	**DFT**	
805.6	706.5	917.5	0.288	0.284	186

[a] measured at P/P_0 = 0.95.

Henry constants and adsorption heats

Gas adsorption isotherms at 273 K and 298 K were fitted by virial equation (S1) in order to calculate Henry constants and isosteric heats of adsorption:

$$lnp = lnn + \frac{1}{T}\sum_{i} A_i \cdot n^i + \sum_{j} B_j \cdot n^j \quad \text{(S1)}$$

Virial coefficients are summarized in Table S4, whereas fit plots are shown in Figure S11.

Table S4. Virial coefficients A_i and B_j for gas adsorption isotherms at 273 K and 298 K on **1**.

Gas	Coefficients	R^2
C_2H_2	$A_0 = -3357$, $A_1 = -162.3$, $A_2 = -57.39$, $A_3 = 33.6$, $B_0 = 8.804$, $B_1 = 0.9259$	0.99992
C_2H_4	$A_0 = -3387$, $A_1 = -291.4$, $A_2 = 431.8$, $A_3 = -409.1$, $A_4 = 167$, $A_5 = -42.25$, $A_6 = -6.634$, $B_0 = 8.758$, $B_1 = 1.316$, $B_2 = -1.156$, $B_3 = 0.9555$, $B_4 = -0.1868$	1.00000
C_2H_6	$A_0 = -3704$, $A_1 = 782.3$, $A_2 = -2002$, $A_3 = 1904$, $A_4 = -837.1$, $A_5 = 136.4$, $B_0 = 9.317$, $B_1 = -2.338$, $B_2 = 7.545$, $B_3 = -7.517$, $B_4 = 3.702$, $B_5 = -0.7726$, $B_6 = 0.04396$	1.00000
CH_4	$A_0 = -2332$, $A_1 = 124.3$, $B_0 = 8.097$	0.99999
CO_2	$A_0 = -3045$, $B_0 = 9.281$, $B_1 = 0.2631$, $B_2 = 0.05353$	0.99996
CO	$A_0 = -1456$, $B_0 = 5.552$, $B_1 = 10.15$, $B_2 = -42.35$, $B_3 = 58.74$	0.99630
N_2	$A_0 = -1801$, $B_0 = 7.891$, $B_1 = 0.5999$	0.99993
O_2	$A_0 = -1634$, $B_0 = 7.382$	0.99087

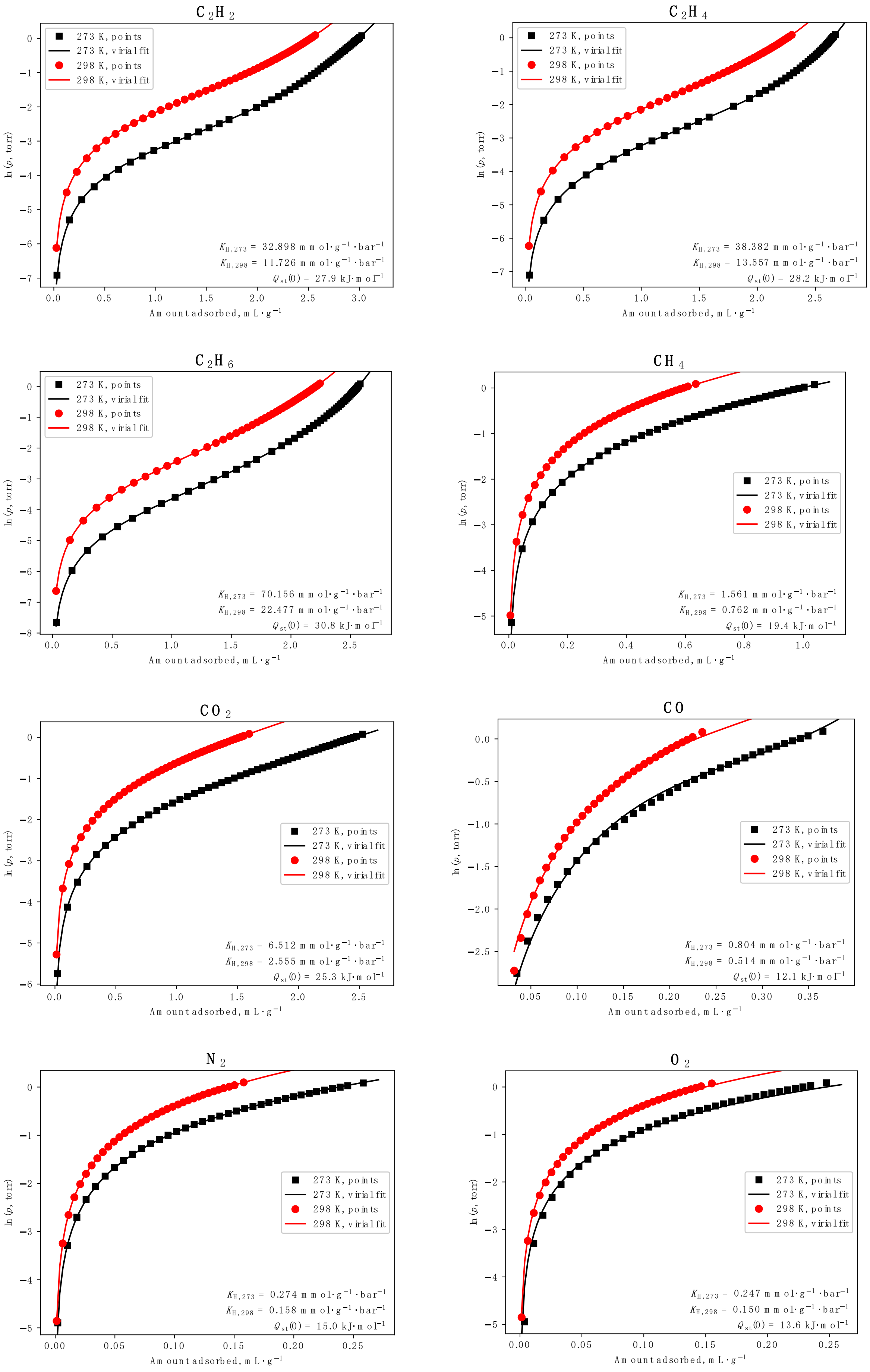

Figure S11. Fits of isotherms by virial equation.

Henry constants were calculated using virial coefficients by equation (S2):

$$K_h = exp\left[\frac{-A_0}{T} - B_0\right] \quad \text{(S2)}$$

They are summarized in Table S5.

Table S5. Henry constants for gas adsorption on **1** in mmol·g^{-1}·bar^{-1} at 273 K and 298 K obtained by virial approach.

**Gas\Temperature**	**273 K**	**298 K**
C_2H_2	32.898	11.726
C_2H_4	38.382	13.557
C_2H_6	70.156	22.477
CH_4	1.561	0.762
CO_2	6.512	2.555
CO	0.804	0.514
N_2	0.274	0.158
O_2	0.247	0.150

Isosteric heats of adsorption were calculated using virial coefficients by equation (S3):

$$Q_{\text{st}} = -R \cdot \sum_i A_i \cdot n^i \quad \text{(S3)}$$

The corresponding values are in Table S6, the corresponding graphs are shown in Figure S12.

Table S6. Zero coverage heats of adsorption in kJ/mol.

**Gas**	**Q_{st}(0), kJ/mol**
C_2H_2	27.9
C_2H_4	28.2
C_2H_6	30.8
CH_4	19.4
CO_2	25.3
CO	12.1
N_2	15.0
O_2	13.6

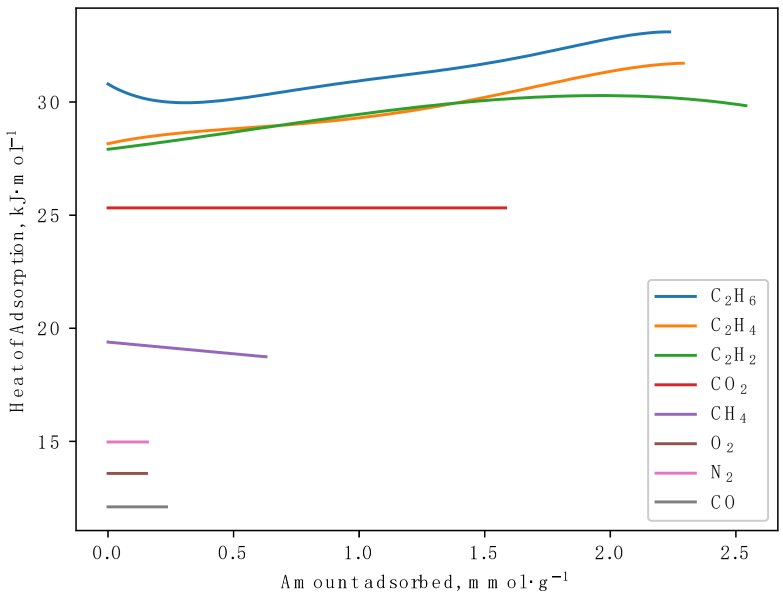

Figure S12. Isosteric heats of gas adsorption on **1** calculated by a virial approach.

IAST calculations

Adsorption isotherms were fitted by the most appropriate model for IAST calculations. Fittings were performed for isotherms in mL/g–torr units, so, the parameters are in the corresponding units. The models used and the corresponding fitted parameters are summarized in Table S7. The fitted isotherms are shown in Figure S13. The choices of the models have been made based on the fitting results including R^2, a residual sum of squares, the parameters errors and a good coincidence by eye. As a result, the adsorption isotherms for all gases can be satisfactory described by the Langmuir or the Dual-Site Langmuir equations:

Langmuir model: $n[\mathrm{mL/g}] = \frac{wbp[\mathrm{torr}]}{1+bp[\mathrm{torr}]}$

Dual-Site Langmuir model: $n[\mathrm{mL/g}] = \frac{w_1 b_1 p[\mathrm{torr}]}{1+b_1 p[\mathrm{torr}]} + \frac{w_2 b_2 p[\mathrm{torr}]}{1+b_2 p[\mathrm{torr}]}$.

The consistency of isotherms fitting by an appropriate model is also confirmed by comparison of the Henry constants calculated both by a virial approach and from the fitted parameters obtained. By definition, a Henry constant is a limit at $p \to 0$ of the amount adsorbed with respect to pressure:

$$K_H = \lim_{p \to 0} \left(\frac{dn(p)}{dp} \right)$$

For a multi-site Langmuir equation K_H can be easily calculated:

$$K_H = \lim_{p \to 0} \left(\frac{d \sum_i \left(\frac{w_i b_i p}{1 + b_i p} \right)}{dp} \right) = \sum_i w_i \, b_i$$

Table S7. Fitted parameters for the adsorption isotherms on **1** at 273 K and 298 K and the corresponding Henry constants for comparison obtained by fitting and calculations done using a virial approach, in brackets the deviations from a virial Henry constant are given.

Gas	Final set of parameters		Henry constant, mmol·g^{-1}·bar^{-1}	
	273 K	298 K	273 K	298 K
C_2H_2	Langmuir<br>$w = 71.68$, $b = 0.01582$,<br>$R^2 = 0.99912$	Langmuir<br>$w = 70.45$, $b = 0.005477$,<br>$R^2 = 0.99971$	37.94 (+15.3%)	12.91 (+10.1%)
C_2H_4	Langmuir<br>$w_1 = 63.15$, $b_1 = 0.01811$,<br>$R^2 = 0.99973$	Langmuir<br>$w_1 = 60.6$, $b_1 = 0.006585$,<br>$R^2 = 0.99992$	38.27 (–0.3%)	13.35 (–1.5%)
C_2H_6	Dual-Site Langmuir<br>$w_1 = 16.61$, $b_1 = 0.003248$,<br>$w_2 = 47.09$, $b_2 = 0.04256$<br>$R^2 = 1.00000$	Dual-Site Langmuir<br>$w_1 = 19.36$, $b_1 = 0.001045$,<br>$w_2 = 44.74$, $b_2 = 0.01476$<br>$R^2 = 1.00000$	68.87 (–1.8%)	22.77 (+1.3%)
CH_4	Langmuir<br>$w = 64.01$, $b = 0.0007063$<br>$R^2 = 0.99999$	Langmuir<br>$w = 60.17$, $b = 0.0003772$<br>$R^2 = 1.00000$	1.513 (–3.1%)	0.759 (–0.3%)
CO_2	Langmuir<br>$w = 89.93$, $b = 0.002081$,<br>$R^2 = 0.99997$	Langmuir<br>$w = 84.78$, $b = 0.0008919$,<br>$R^2 = 0.99999$	6.262 (–3.8%)	2.530 (–1.0%)
CO	Langmuir<br>$w = 33.96$, $b = 0.0003819$<br>$R^2 = 0.99918$	Langmuir<br>$w_1 = 16.37$, $b_1 = 0.0005653$<br>$R^2 = 0.99483$	0.434 (–46.0%)	0.310 (–39.7%)
N_2	Langmuir<br>$w = 48.61$, $b = 0.0001652$<br>$R^2 = 0.99999$	Langmuir<br>$w = 42.08$, $b = 0.0001111$<br>$R^2 = 0.99997$	0.269 (–1.9%)	0.156 (–1.0%)
O_2	Langmuir<br>$w = 48.59$, $b = 0.0001567$<br>$R^2 = 0.99999$	Langmuir<br>$w = 48.57$, $b = 0.00009501$<br>$R^2 = 0.99999$	0.255 (+3.1%)	0.154 (+2.9%)

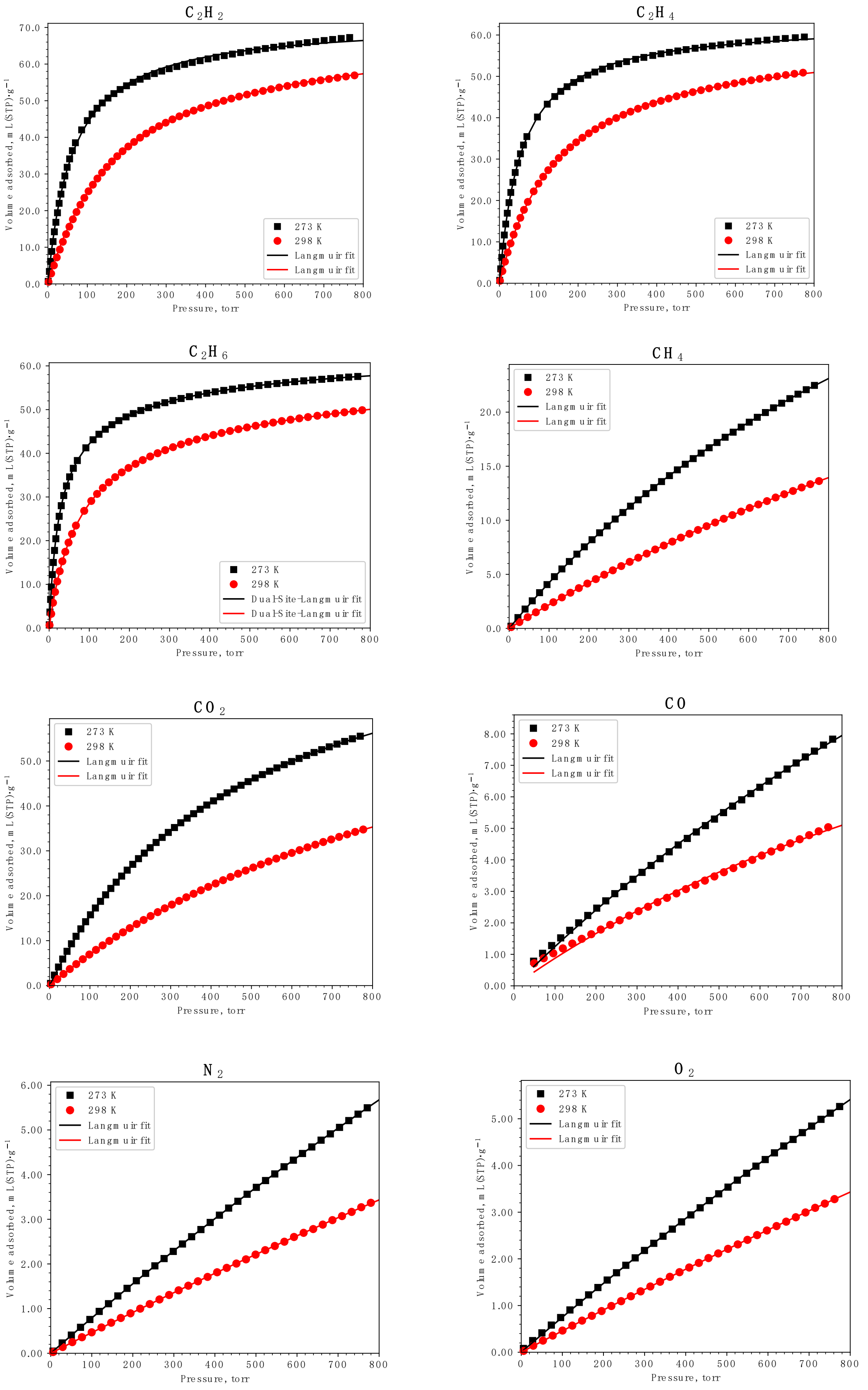

Figure S13. Fits of isotherms by an appropriate model.

Selectivity factors were evaluated by three commonly used methods:

(i) As the molar ratio of the adsorption quantities at the relevant partial pressures of the gases:

$$S = \frac{n_1/n_2}{p_1/p_2}, \tag{S4}$$

where S is the selectivity factor, n_i represents the adsorbed amount of the component i, and p_i represents the partial pressure of the component i.

(ii) As a ratio of Henry constants which corresponds to the slope of the adsorption isotherm at very low partial pressures:

$$S = \frac{K_{H1}}{K_{H2}} \tag{S5}$$

(iii) By ideal adsorbed solution theory (IAST). The relationship between P, y_i and x_i (P — the total pressure of the gas phase, y_i — mole fraction of the i-component in a gas phase, x_i — mole fraction of the i-component in an absorbed state) is defined according to the IAST theory [41]:

$$\int_{p=0}^{p=\frac{Py_1}{x_1}} n_1\,(p) dlnp = \int_{p=0}^{p=\frac{Py_2}{x_2}} n_2\,(p) dlnp \tag{S6}$$

In this case the selectivity factors are determined as:

$$S = \frac{y_2 x_2}{y_1 x_1} = \frac{x_1(1-y_1)}{y_1(1-x_1)} \tag{S7}$$

For CO_2/N_2, CO_2/CO, CO_2/CH_4, C_2H_6/CH_4, C_2H_4/CH_4, C_2H_2/CH_4 mixtures IAST calculations performed allow us to plot an equilibrium graph as well as a dependence of selectivity factors on a gas mixture composition and a total pressure. All plots are shown in Figure S14 below.

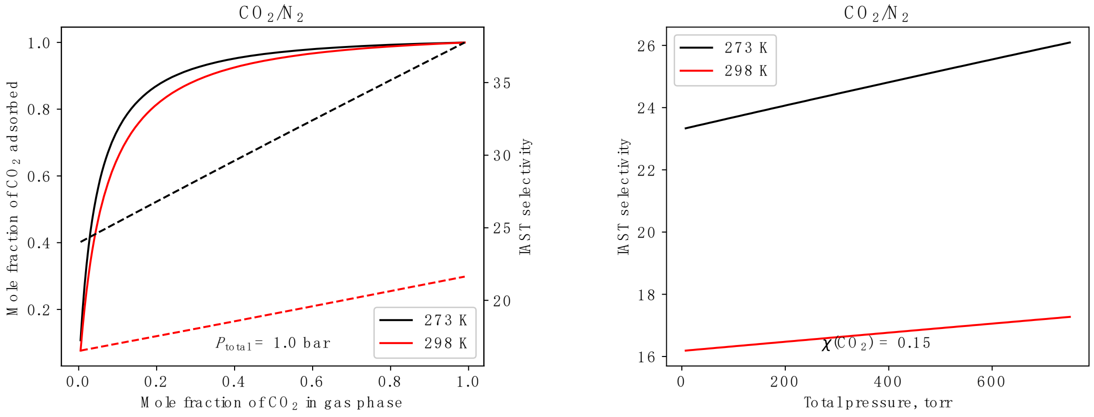

(a)

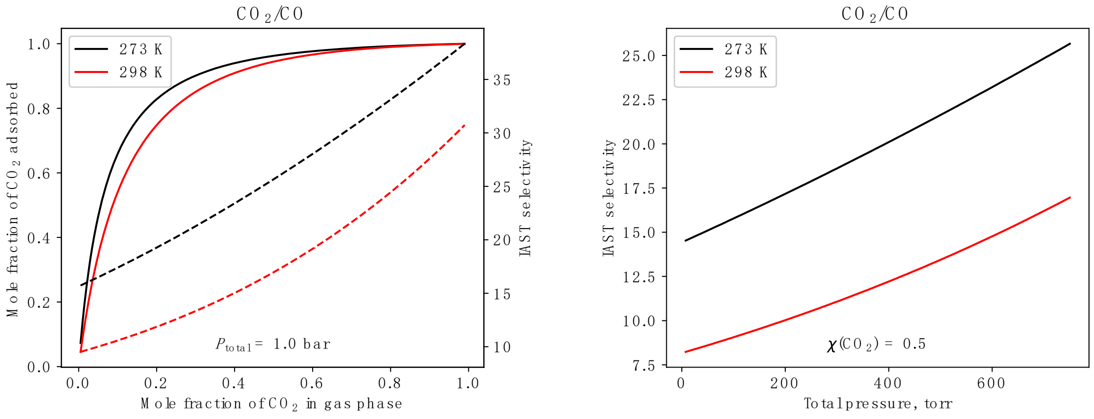
CO2/CO
273 K
298 K
Mole fraction of CO2 adsorbed
IAST selectivity
P_total = 1.0 bar
Mole fraction of CO2 in gas phase
χ(CO2) = 0.5
Total pressure, torr

(b)

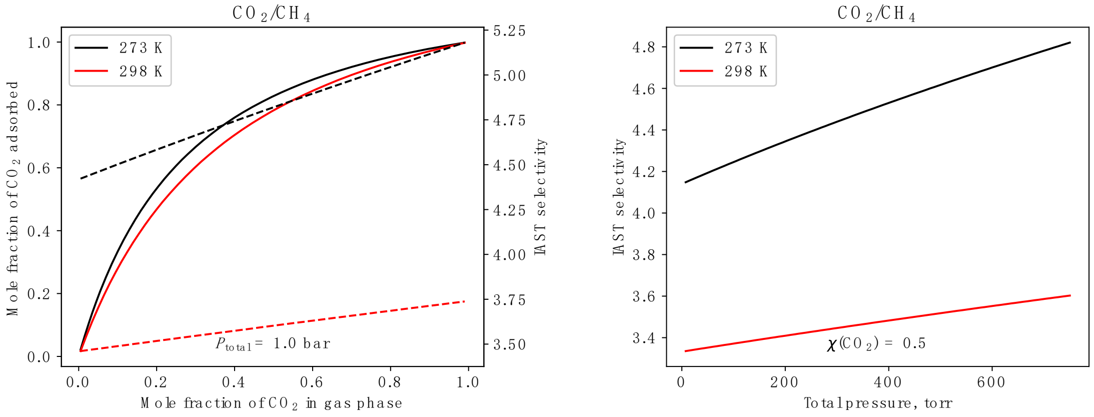
CO2/CH4
273 K
298 K
Mole fraction of CO2 adsorbed
IAST selectivity
P_total = 1.0 bar
Mole fraction of CO2 in gas phase
χ(CO2) = 0.5
Total pressure, torr

(c)

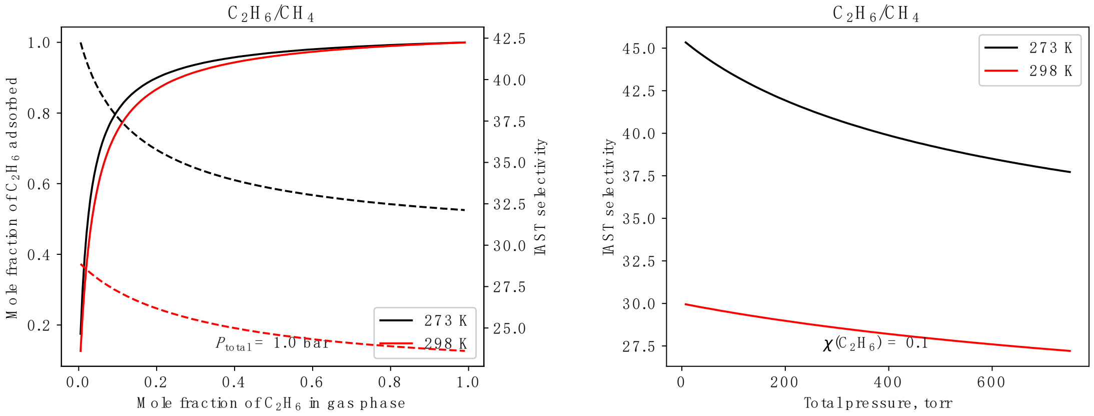
C2H6/CH4
273 K
298 K
Mole fraction of C2H6 adsorbed
IAST selectivity
P_total = 1.0 bar
Mole fraction of C2H6 in gas phase
χ(C2H6) = 0.1
Total pressure, torr

(d)

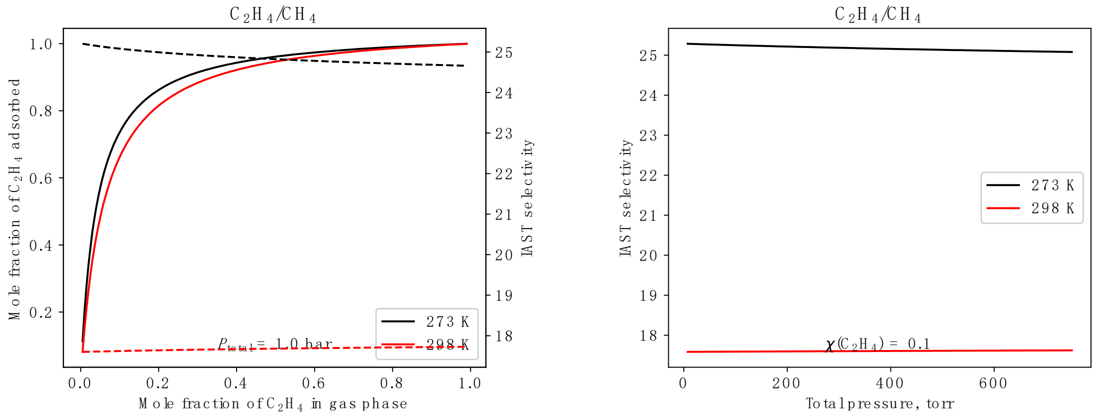

(e)

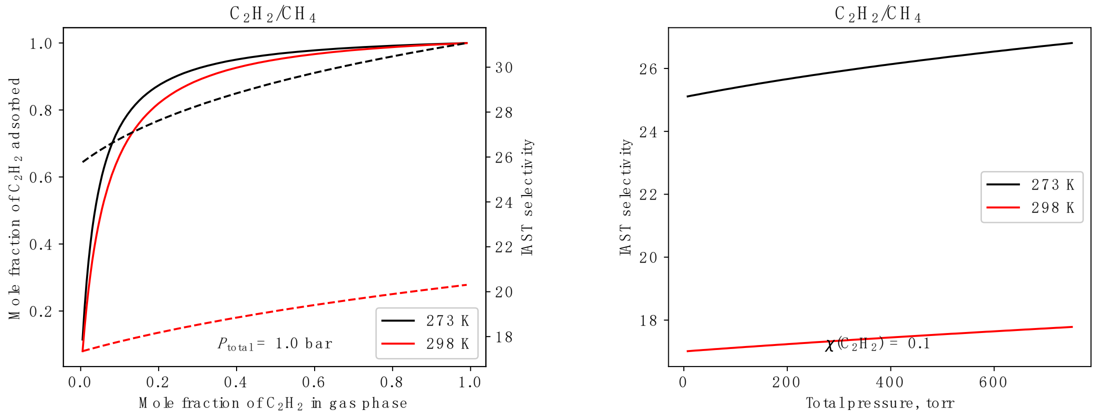

(f)

Figure S14. The prediction of an adsorption equilibrium by IAST (solid lines) and dependence of selectivity factors on a gas phase composition (dashed lines) as well as their pressure dependence for binary gas mixtures: a) CO_2/N_2; b) CO_2/CO; c) CO_2/CH_4; d) C_2H_6/CH_4; e) C_2H_4/CH_4; f) C_2H_2/CH_4.

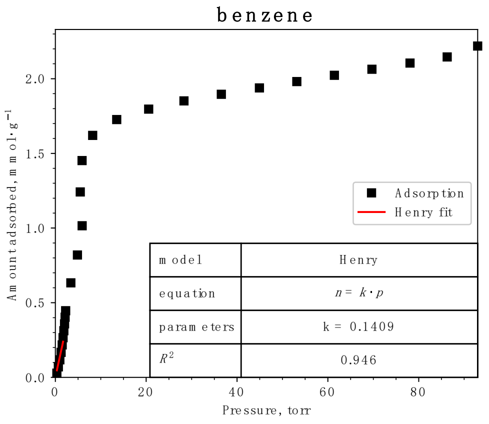

(a)

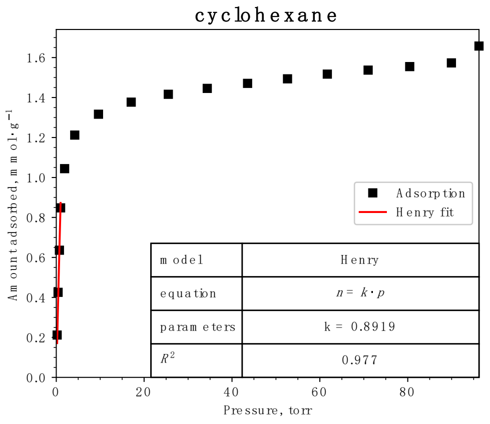

(b)

Figure S15. Fit of the initial (linear) parts of vapor adsorption isotherms by the Henry law, [k] = mmol·g^{-1}·torr^{-1}.

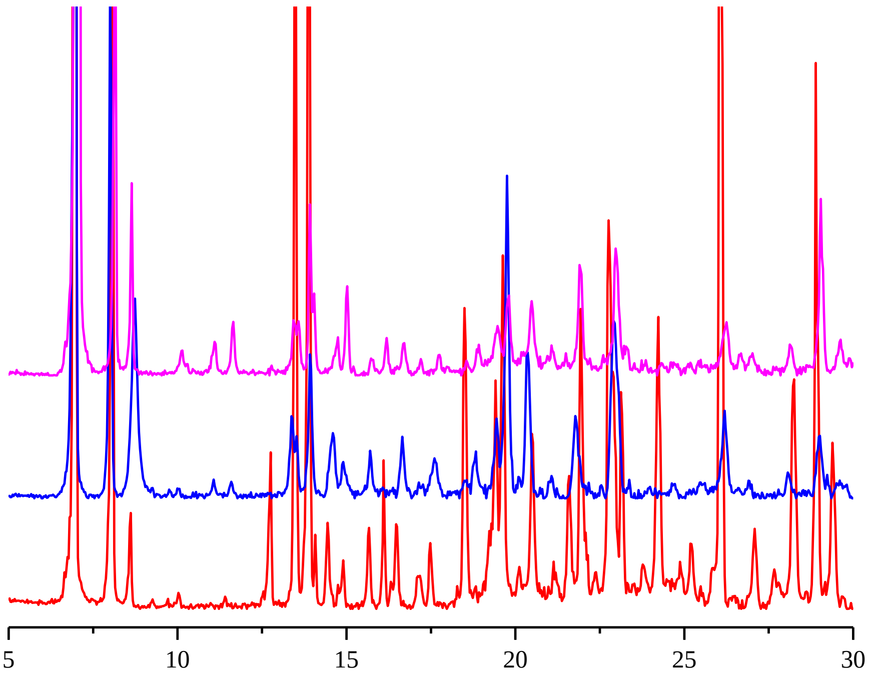

Figure S16. PXRD patterns of **1**: as-synthesized (red), activated (blue), and after adsorption (purple).

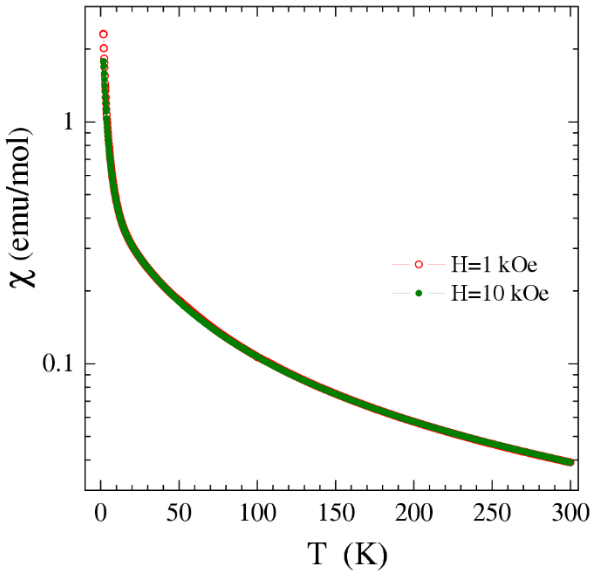

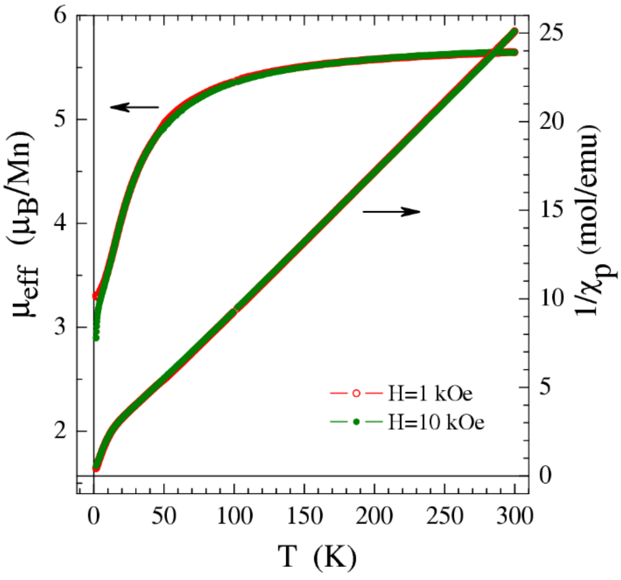

(a) (b)

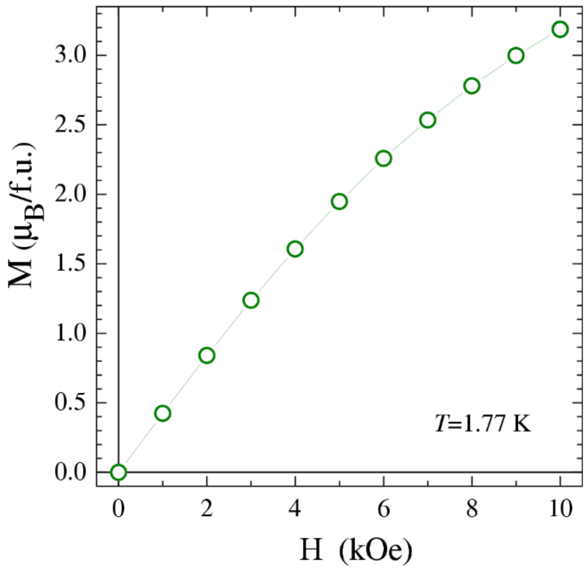

(c)

Figure S17. (a) Temperature dependences of the magnetic susceptibility χ of **1** measured at magnetic fields H = 1; 10 kOe. (b) Temperature dependences of the effective magnetic moment μ_{eff} and the reversed magnetic susceptibility $1/\chi_p$ for **1**. (c) Magnetic-field dependence of the magnetization M measured for **1** at T = 1.77 K.

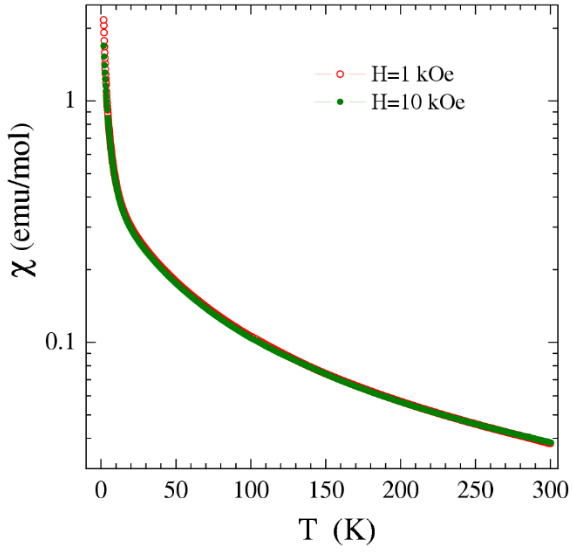

(a)

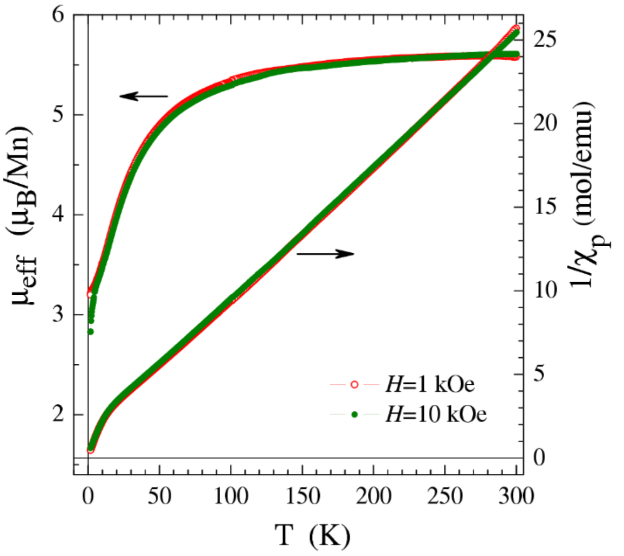

(b)

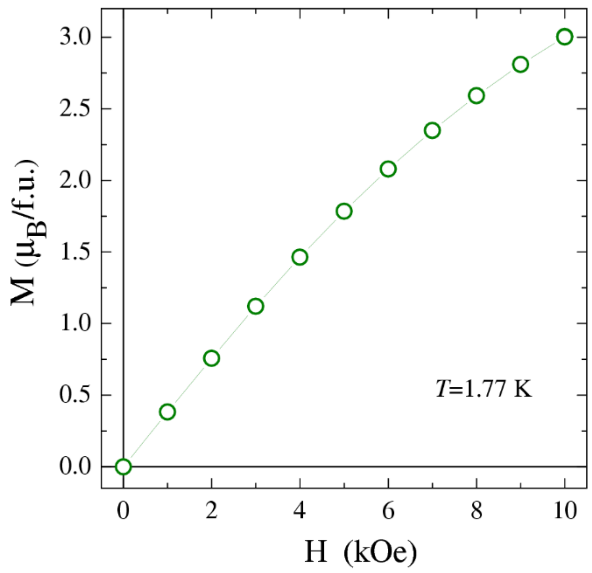

(c)

Figure S18. (a) Temperature dependences of the magnetic susceptibility χ of **2** measured at magnetic fields H = 1; 10 kOe. (b) Temperature dependences of the effective magnetic moment μ_{eff} and the reversed magnetic susceptibility $1/\chi_p$ for **3**. (c) Magnetic-field dependence of the magnetization M measured for **2** at T = 1.77 K.

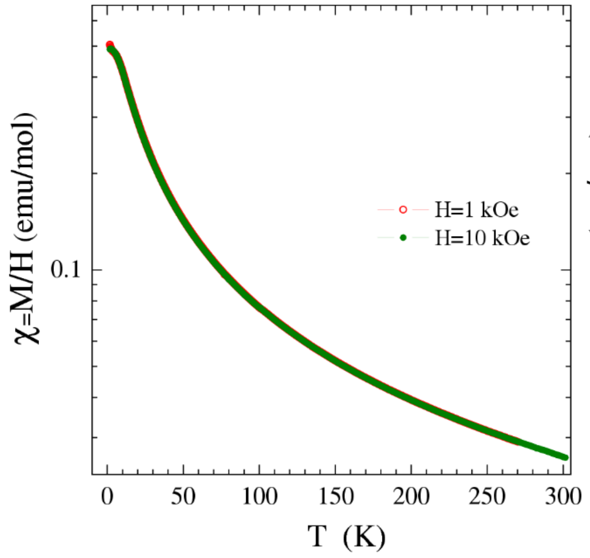

(a)

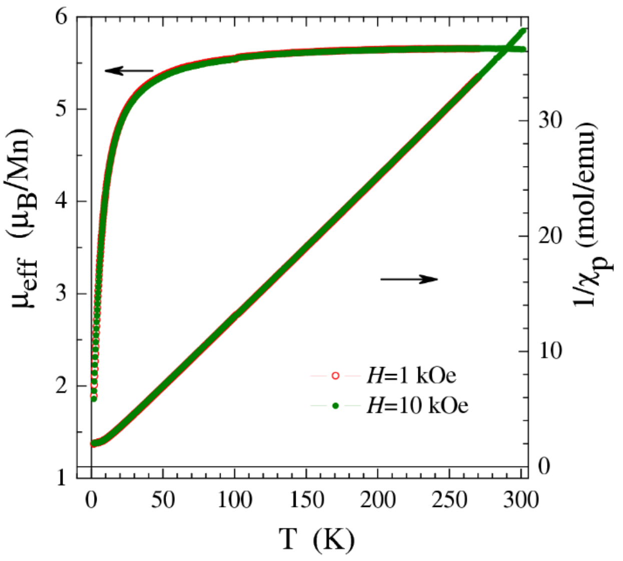

(b)

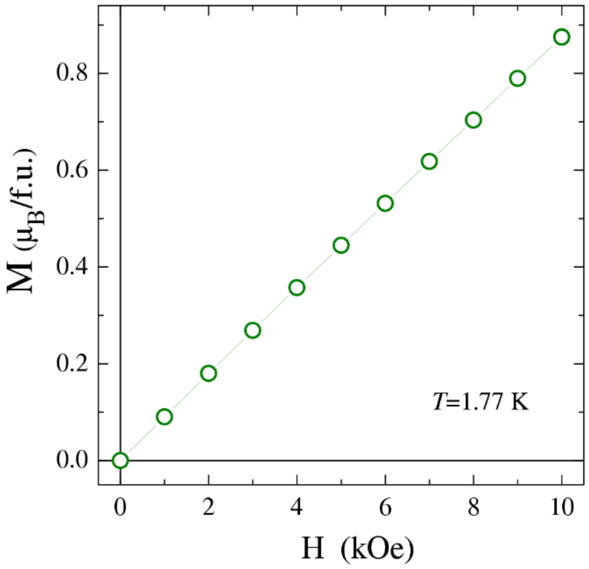

(c)

Figure S19. (a) Temperature dependences of the magnetic susceptibility χ of **3** measured at magnetic fields H = 1; 10 kOe. (b) Temperature dependences of the effective magnetic moment μ_{eff} and the reversed magnetic susceptibility $1/\chi_p$ for **3**. (c) Magnetic-field dependence of the magnetization M measured for **3** at T = 1.77 K.
